# Cytotoxic Activity of Bisphosphonic Derivatives Obtained by the Michaelis–Arbuzov or the Pudovik Reaction

**DOI:** 10.3390/ph18010091

**Published:** 2025-01-13

**Authors:** Zsuzsanna Szalai, Janka Bednárik, Boldizsár Szigfrid Tóth, Angéla Takács, Szilárd Tekula, László Kőhidai, Konstantin Karaghiosoff, László Drahos, György Keglevich

**Affiliations:** 1 Department of Organic Chemistry and Technology, Faculty of Chemical Technology and Biotechnology, Budapest University of Technology and Economics, Műegyetem rkp. 3, 1111 Budapest, Hungary; szalai.zsuzsanna@edu.bme.hu (Z.S.); janka.bednarik@edu.bme.hu (J.B.); tothszigfridboldizsar@edu.bme.hu (B.S.T.); 2 Department of Genetics, Cell and Immunobiology, Semmelweis University, Nagyvárad tér 4, 1089 Budapest, Hungary; takacs.angela@semmelweis.hu (A.T.); kohidai.laszlo@semmelweis.hu (L.K.); 3 Department Chemie, Ludwig-Maximilians-Universität München, Butenandtstr. 5-13, D-81377 München, Germany; klk@cup.uni-muenchen.de; 4 MS Proteomics Research Group, Research Centre for Natural Sciences, 1117 Budapest, Hungary; drahos.laszlo@ttk.hu

**Keywords:** bisphosphonates, dronates, Arbuzov reaction, Pudovik reaction, X-ray structure, biological activity, ethyl diphenylphosphinite, trialkyl phosphite, dialkyl phosphite, secondary phosphine oxide

## Abstract

**Background:** Methylenebisphosphonic derivatives including hydroxy-methylenebisphosphonic species may be of potential biological activity, and a part of them is used in the treatment of bone diseases. **Methods:** Methylenebisphosphonates may be obtained by the Michaelis–Arbuzov reaction of suitably α-substituted methylphosphonates and trialkyl phosphites or phosphinous esters, while the hydroxy-methylene variations are prepared by the Pudovik reaction of α-oxophosphonates and different >P(O)H reagents, such as diethyl phosphite and diarylphosphine oxides. **Results:** After converting α-hydroxy-benzylphosphonates and -phosphine oxides to the α-halogeno- and α-sulfonyloxy derivatives, they were utilized in the Michaelis–Arbuzov reaction with trialkyl phosphites and ethyl diphenylphosphinite to afford the corresponding bisphosphonate, bis(phosphine oxide) and phosphonate–phosphine oxide derivatives. The Pudovik approach led to α-hydroxy-methylenebisphosphonic species and to their rearranged products. A part of the derivatives revealed a significant cytotoxic effect on pancreatic adenocarcinoma or multiple myeloma cells. **Conclusions:** The new families of compounds synthesized by our novel approaches may be of practical importance due to the significant cytotoxic activity on the cell cultures investigated. Compounds lacking hydroxy groups showed anti-myeloma activity or limited effect on pancreatic cancer (PANC-1) cells unless substituted with para-trifluoromethyl group. Hydroxy-containing bisphosphonates and their rearranged derivatives demonstrated varying effects depending on structural modifications. While myeloma (U266) cells indicated greater sensitivity overall, the most significant reductions in cell viability were observed in PANC-1 cancer cells, raising potential therapeutic applications of bisphosphonates beyond myeloma-associated bone disease, particularly for malignancies like pancreatic ductal adenocarcinoma.

## 1. Introduction

α-Substituted hydroxy-methylenebisphosphonic (dronic) derivatives are used to treat bone diseases, such as osteoporosis, Paget’s disease and tumor-induced hypercalcemia, but they also show antitumor and antiparasitic effects [1,2,3,4,5,6,7,8]. The two phosphonic groups may form a complex with the calcium ion, so their absorption may be prevented. The most often used method for their synthesis involves the reaction of amino acids or heteroaryl carboxylic acids with phosphorus trichloride and phosphorous acid in methanesulfonic acid or sulfolane as the solvent [9].

The other possibility for the preparation of hydroxy-bisphosphonates involves the addition of dialkyl phosphites to α-oxophosphonates, which is the Pudovik reaction [10,11,12]. In these cases, depending on the conditions, rearrangement may have accompanied the addition [13]. We also investigated the Pudovik reaction of α-oxophosphonates and dialkyl phosphites or diarylphosphine oxides. It was found that the rearrangement mainly depended on the amount of the base catalyst and the substituent of the oxophosphonate [14].

The bisphosphonate derivatives that do not contain a hydroxy group on the α-carbon atom may be prepared by the reaction of diiodomethane and two equivalents of trialkyl phosphite. The double Arbuzov reaction leading to tetraalkylbisphosphonates may be followed, for example, by alkylation [15] or acylation to afford α-substituted methylenebisphosphonates. A special method for the preparation of tetraalkyl bisphosphonates involves the reaction of hydroxyphosphonates—formed from aldehydes and dialkyl phosphites—with mesyl chloride. Researchers have claimed that this intermediate reacts easily with another unit of dialkyl phosphite to furnish the target bisphosphonate in a one-pot reaction [16].

We found that α-methanesulfonyloxy-benzylphosphonates may react with triethyl phosphite in the Michaelis−Arbuzov reaction. This is a better method for the preparation of bisphosphonic derivatives than the reaction of α-halogeno-benzylphosphonates with triethyl phosphite [17].

The adducts of the α-oxophosphonates and >P(O)H reagents and their rearranged species were subjected to in vitro cytostatic activity study on four different cell cultures (breast (MDA-MB 231), epidermoid (A431), prostate (PC-3), and lung carcinoma (Ebc-1)). This structure-activity study revealed cytostatic effects for the rearranged derivatives containing aromatic units [14].

In this article, we aimed at the synthesis of new bisphosphonic derivatives in a broader sense by the Michaelis–Arbuzov reaction of sulfonyloxyphosphonates. The other purpose was to prepare a few new symmetric and asymmetric hydroxy-methylenebisphosphonic derivatives. The final goal was to test the cytotoxic activity of our products synthesized.

## 2. Results and Discussion

### 2.1. Synthesis

**Scheme 1:**
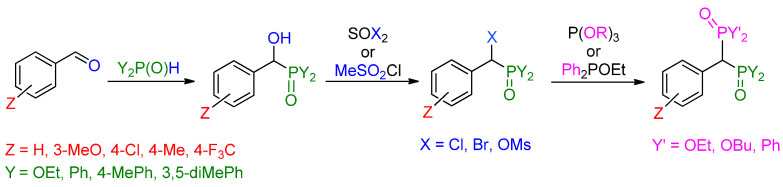
Our general process for preparing bisphosphonate type compounds.

During our synthetic work, we followed the reaction sequences outlined in Scheme 1. This involves the preparation of the starting hydroxyphosphonates, their conversion to the α-halogeno and α-methanesulfonyloxy intermediates, and their involvement in Michaelis–Arbuzov reactions.

#### 2.1.1. Preparation of the Starting α-Hydroxy-, α-Halogeno- and α-Methanesulfonyloxy-benzylphosphonate Derivatives

**Scheme 2:**
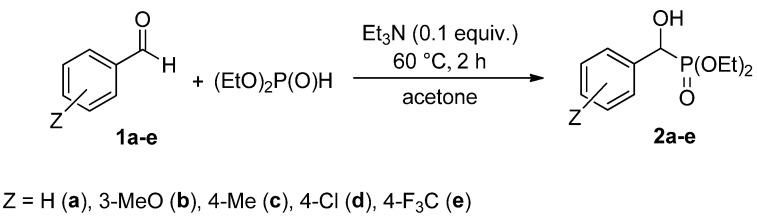
Preparation of diethyl α-hydroxy-benzylphosphonates (**2a**–**e**).

The starting α-hydroxyphosphonates (**2a**–**e**) were prepared by the method developed in our research group earlier [18]. The mixture of substituted benzaldehyde (**1a**–**e**) and a stochiometric amount of diethyl phosphite was refluxed in the presence of 0.1 equiv. of triethylamine for 2 h in acetone as the solvent, then n-pentane was added, and the mixture cooled to 5 °C, whereupon α-hydroxy-benzylphosphonates **2a** and **2c**–**e** crystallized out. The crystals were filtered off and washed with pentane. Product **2b** was obtained as an oil, and it was purified by column chromatography (Scheme 2).

**Scheme 3:**
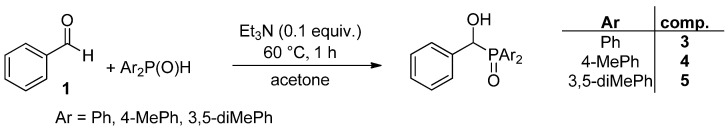
Synthesis of diaryl α-hydroxy-benzylphosphine oxides (**3**–**5**).

The procedure described above was extended to the synthesis of α-hydroxy-benzylphosphine oxides (**3**–**5**). In this case, 1 h of reflux was sufficient. Diaryl α-hydroxy-benzylphosphine oxides **3**–**5** were obtained as white crystalline products (Scheme 3).

The identity of hydroxyphosphonates (**2a**–**e**) and hydroxyphosphine oxides (**3**–**5**) isolated in 71–89% and 76–83% yields, respectively, was supported by their ^31^P NMR shifts and by LC-MS analysis.

**Scheme 4:**
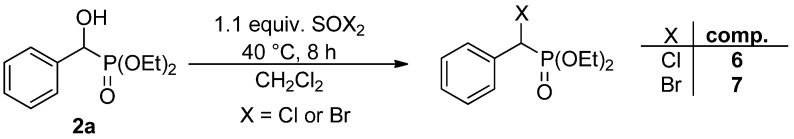
Preparation of diethyl α-chloro- and α-bromo-benzylphosphonates (**6** and **7**).

The α-halogenophosphonates were prepared by the method described by Keglevich and Grün et al. [19] earlier. Diethyl α-hydroxy-benzylphosphonate **2a** was reacted with 1.1 equiv. of thionyl chloride or thionyl bromide at 40 °C in dichloromethane for 8 h. After purification by chromatography, the expected α-chloro-benzylphosphonate **6** and α-bromo-benzylphosphonate **7** were isolated in 81/83% yields, and they were identified by ^31^P NMR shifts, as well as HPLC-MS (Scheme 4).

**Scheme 5:**
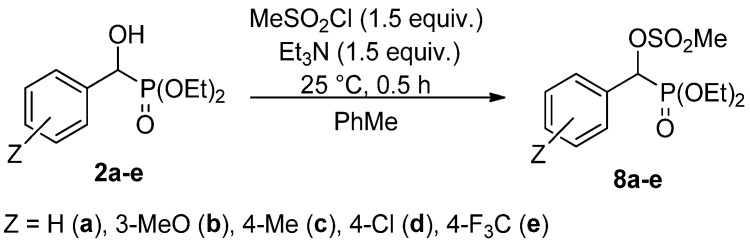
Synthesis of diethyl α-methanesulfonyloxy-benzylphosphonates (**8a**–**e**).

α-Hydroxy-benzylphosphonates **2a**–**e** were reacted with 1.5 equiv. of methanesulfonyl chloride at 25 °C in the presence of 1.5 equiv. of triethylamine in toluene for 0.5 h to afford products **8a**–**e** in 76–84% yields after purification by chromatography (Scheme 5). The mesyloxyphosphonates **8a**–**d** were described by us earlier [17] and were then identified by δ_P_ their shifts and HPLC. Diethyl α-methanesulfonyloxy-4-trifluoromethylbenzylphosphonates (**8e**) is a new compound and was fully characterized.

**Scheme 6:**
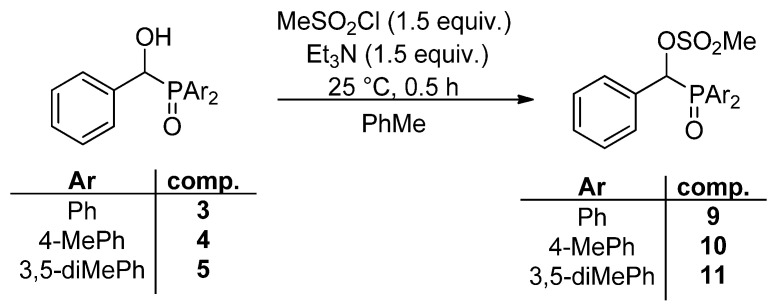
Synthesis of diaryl α-methanesulfonyloxy-benzylphosphine oxides (**9**–**11**).

Hydroxy-benzylphosphine oxides **3**–**5** were also reacted with methanesulfonyl chloride under similar conditions to afford products **9**–**11** in 78–98% yields after purification by chromatography (Scheme 6). Diaryl α-methanesulfonyloxy-benzylphosphine oxides **9**–**11** are new compounds, and were fully characterized by ^31^P, ^13^C, ^1^H NMR spectroscopy and HRMS.

#### 2.1.2. X-Ray Diffraction Studies of α-Methanesulfonyloxy-benzylphosphonate Derivatives

Mesyloxy-benzylphosphonate **8e** and mesyloxy-benzylphosphine oxide **11** were subjected to single crystal X-ray diffraction analysis.

**Figure 1:**
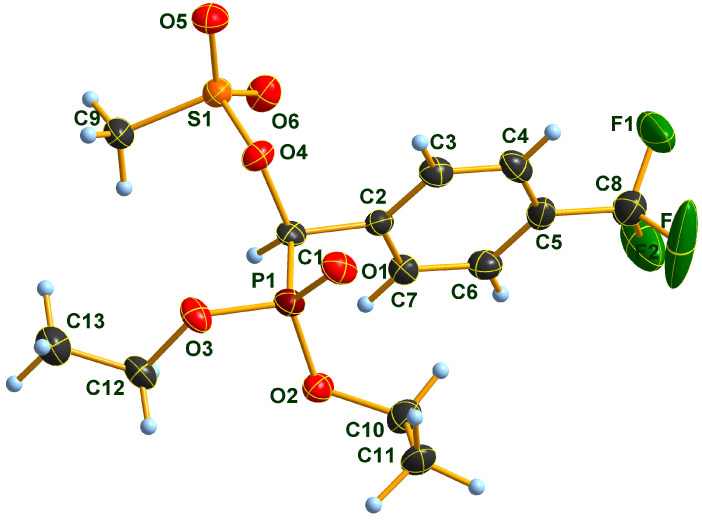
Molecular structure of sulfonyloxyphosphonate **8e** in the crystal. DIAMOND [20] representation; thermal ellipsoids are drawn at 50% probability level.

Sulfonyloxyphosphonate **8e** crystallizes in the monoclinic space group *P*21/*c* with four molecules in the unit cell. The asymmetric unit comprises one molecule of **8e**. A view of the molecule in the crystal is shown in Figure 1. Atom distances and bond angles are in the expected range. The phosphorus and sulfur atoms are part of distorted tetrahedral environments with angles ranging from 104.0(2)° to 113.4(2)° for the phosphorus atom and from 103.6(2)° to 120.1(2)° for the sulfur atom. The molecules adopt a staggered conformation along the P-C bond (1.827(5) Å) corresponding to a minimum repulsion between the oxygen atoms at phosphorus and O4 at the sulfur atom.

**Figure 2:**
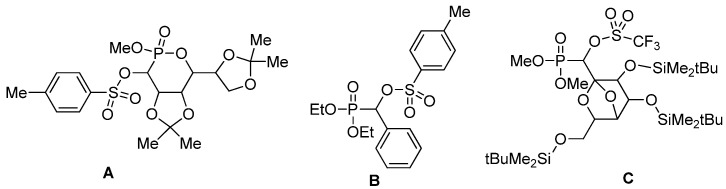
Compounds (**A**–**C**) for comparison described in the literature.

The molecular and crystal structures of only three more or less analogous compounds (**A**–**C**) [21,22,23] have been described in the literature (Figure 2). Compound **B** [22] is the best for comparison purposes. The structures of sulfonyloxyphosphonates **8e** and **B**, in fact, fit well with each other.

**Figure 3:**
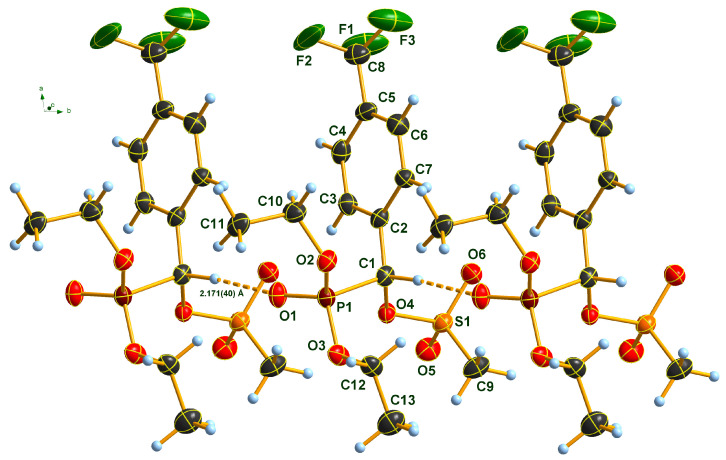
Hydrogen bonding in the crystal structure of sulfonyloxyphosphonate **8e**; view of the hydrogen bonded chains along the *b*–axis. DIAMOND [20] representation; thermal ellipsoids are drawn at 50% probability level. Symmetry code for the left non-labeled molecule: x, −1 + y, z; symmetry code for the right non-labeled molecule: x, 1 + y, z.

In the crystal structure of phosphonate **8e**, non-classical hydrogen bonds between the hydrogen atom at C1 and oxygen atom at phosphorus O1 result in the formation of chains along the *b–axis* (Figure 3). The structural parameters of the hydrogen bonds are: D–H: 0.99(4) Å, A···H: 2.17(4) Å, D···A: 3.157(5) Å, D–H···A: 170.6(30)°. Remarkably, the oxygen atoms at sulfur do not participate in significant hydrogen bonding interactions.

**Figure 4:**
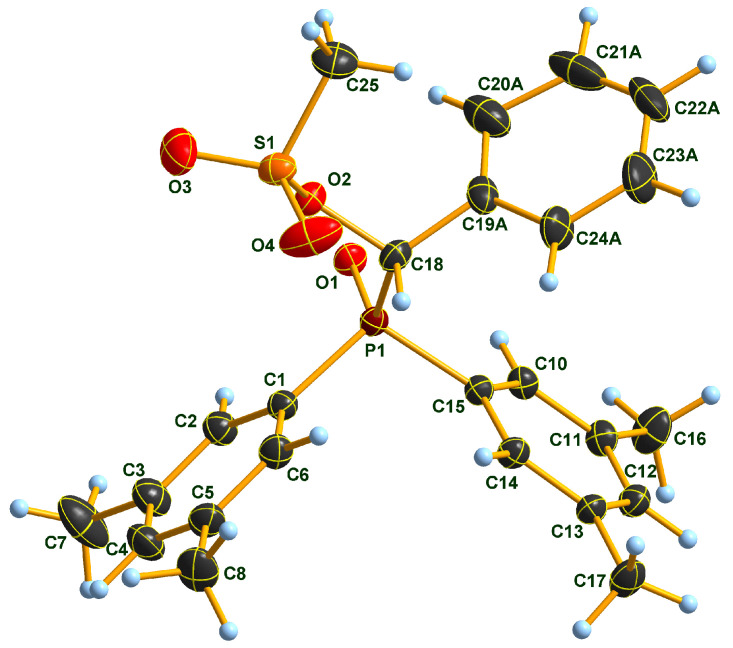
Molecular structure of sulfonyloxyphosphine oxide **11** in the crystal. DIAMOND [20] representation; thermal ellipsoids are drawn at 50% probability level. The C-bonded phenyl ring is disordered over two positions. Only the stronger populated position (60%) has been shown for clarity.

Sulfonyloxyphosphine oxide **11** crystallizes in the orthorhombic space group *Pna*21 with four formula units in the unit cell. The asymmetric unit comprises one molecule of **11**. The molecular structure is shown in Figure 4. The phenyl ring bonded to C18 is disordered over two positions. Only one of these positions is shown for clarity. All atom distances and bond angles are in the expected range. The also molecule adopts in this case a staggered conformation along the P-C bond to C18 in order to minimize steric repulsion.

**Figure 5:**
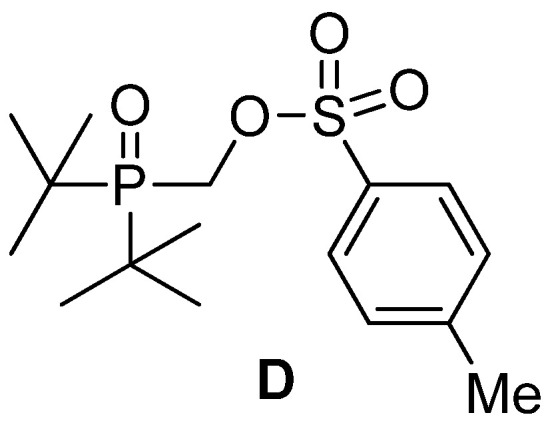
Compound **D** for comparison described in the literature.

There is only one compound (**D**) [24] reported in the literature showing a similar structural motif (Figure 5). Compound **D** has *t*Bu-substituents attached at phosphorus instead of the aryl substituents in analogue **11,** and there is no further substituent at the carbon atom between the phosphorus atom and the tosyl group. The structural features of the two compounds fit well with each other.

**Figure 6:**
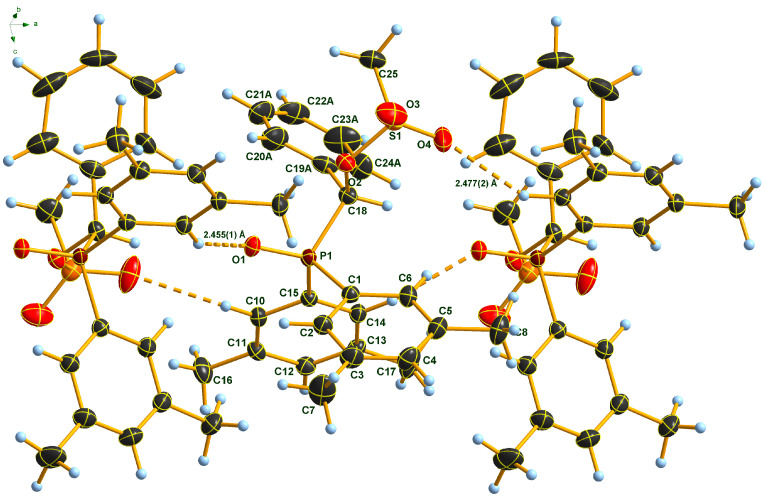
Hydrogen bonding in the crystal structure of sulfonyloxyphosphine oxide **11**; view of the hydrogen-bonded chains along the *a–axis*. DIAMOND [20] representation; thermal ellipsoids are drawn at 50% probability level. Symmetry code for the left non-labelled molecule: −0.5 + x, 1.5 − y, z; symmetry code for the right non-labelled molecule: 0.5 + x, 1.5 − y, z.

As compared to phosphonate **8e**, phosphine oxide **11** has aryl rings instead of the ethyl groups bonded to phosphorus. This has consequences for the intermolecular interactions in the crystal. One non-classical hydrogen bond between the oxygen atom O1 at phosphorus and the hydrogen atom at C14 of one of the aryl substituents results in the formation of chains along the *a*–axis (Figure 6). This interaction is weaker as compared to that observed for analogue **8e** (D–H: 0.950(2) Å, H···A: 2.455(1) Å, D···A: 3.327(2) Å, D–H···A: 152.6(1)°). An interaction of similar strength is observed between O4 bonded to sulfur and the hydrogen atom at C10 of the same *P*-bonded aryl substituent: D–H: 0.950(2) Å, H···A: 2.477(2) Å, D···A: 3.319(3) Å, D–H···A: 147.6(1)°. This hydrogen bonding interaction, involving the sulfonyl group, favors further within a co-operative effect the formation of the chain.

#### 2.1.3. Michaelis–Arbuzov Reaction of α-Halogeno-benzylphosphonates

**Scheme 7:**
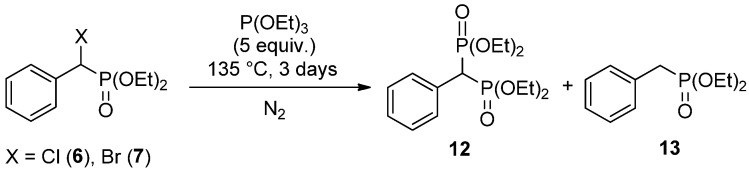
Michaelis–Arbuzov reaction of α-halogeno-benzylphosphonates (**6** and **7**) with triethyl phosphite.

Previously, we observed that the Arbuzov reaction of α-chloro- and even α-bromo-benzylphosphonates (**6** and **7**) with triethyl phosphite yielded the desired bisphosphonate derivative (**12**) in only low conversions together with by-products including diethyl benzylphosphonate (**13**), along with diethyl phosphite and diethyl ethylphosphonate (Scheme 7) [17].

Then, we investigated the Arbuzov reaction of α-halogeno-benzylphosphonates (**6** and **7**) with ethyl diphenylphosphinite to afford phosphonate–phosphine oxide **14a**. In these cases, the reactions took place with a higher conversion, but by-products diethyl benzylphosphonate **13**, ethyldiphenylphosphine oxide and ethyl diphenylphosphinate were also present in the mixtures. Starting from α-chlorophosphonate (**6**), the conversion was 90%, and benzylphosphonate (**13**) was formed in 25% (Table 1, entry 1). Applying α-bromophosphonate (**7**), the conversion was 94%, but the proportion of by-product **13** in the mixture was even higher (Table 1, entry 2).

#### 2.1.4. Michaelis–Arbuzov Reaction of α-Methanesulfonyloxy-benzylphosphonate Derivatives

**Scheme 8:**
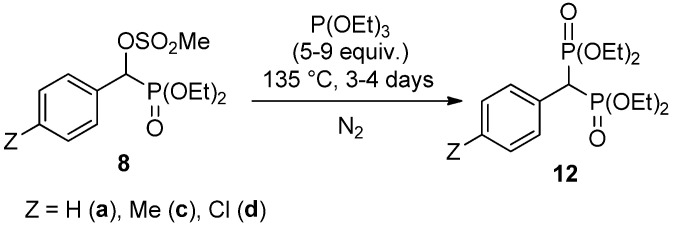
Michaelis–Arbuzov reaction of α-methanesulfonyloxy-benzylphosphonates (**8a**, **8c** and **8d**) with triethyl phosphite.

Earlier, we found that the diethyl α-mesyloxy-benzylphosphonates (**8**) may be efficient reagents in Michaelis–Arbuzov reaction with triethyl phosphite applied in an excess at 135 °C [17]. The corresponding bisphosphonates (**12**) were isolated in good (76–81%) yields after purification by chromatography (Scheme 8). Bisphosphonate **12a** is a known compound that was identified by its δ_P_ shift and MS, while products **12c** and **12d** were described first by us, and, therefore, were characterized by the routine spectral methods [17].

The mesylates (**8a**–**d**) were also reacted with ethyl diphenylphosphinite, leading to variously substituted phosphonate–phosphine oxide derivatives (**14a**–**d**) in a clear-out reaction at 135 °C. In these cases, a 1-day heating proved to be sufficient. By-products ethyldiphenylphosphine oxide and ethyl diphenylphosphinate formed from the excess of the reagent were also present in the crude mixture. Phosphonate–phosphine oxides **14a**–**d** were obtained in yields of 70–86% after chromatography (Table 2), and their structure was confirmed by ^31^P, ^13^C, and ^1^H NMR spectroscopy. Products **14a**–**d** are white crystalline compounds.

The Michaelis–Arbuzov reactions were also extended to mesyloxy-benzylphosphine oxides (**9**–**11**). After heating the mesyloxy derivatives (**9**–**11**) with ethyl diphenylphosphinite at 135 °C for 3 days, the crude product was purified by chromatography. The bis(phosphine oxides) (**15**–**17**) were isolated in yields of 78–98% (Table 3), and were characterized by the usual NMR methods and MS. Products **15**–**17** are white crystalline compounds.

To prepare asymmetric bisphosphonates (**18a**, **18c** and **18e**), α-mesyloxy-benzylphosphonates (**8a**, **8c** and **8e**) were reacted with tributyl phosphite under similar conditions. Three diethyl dibutyl bisphosphonate derivatives (**18a**, **18c** and **18e**) were obtained as oils in yields of 66–91%, after purification by chromatography (Table 4). The structure of the new compounds was proved by the usual NMR methods and MS.

**Scheme 9:**
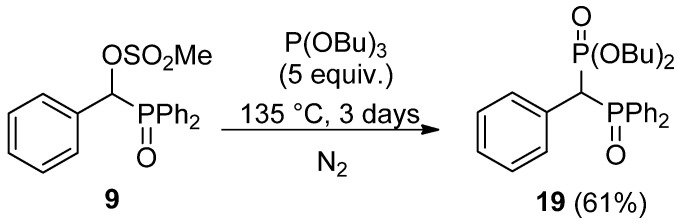
Michaelis–Arbuzov reaction of α-mesyloxy-benzylphosphine oxide **9** with tributyl phosphite.

Finally, mesyloxyphosphine oxide **9** was subjected to the Michaelis–Arbuzov reaction with tributyl phosphite under the conditions applied above (Scheme 9). After purification by chromatography, dibutyl phosphonate–diphenylphosphine oxide **19** was obtained in a yield of 61% as white crystals, and was fully characterized as above.

#### 2.1.5. Pudovik Reaction of α-Oxophosphonates with Dialkyl Phosphites and Diarylphosphine Oxides

**Scheme 10:**
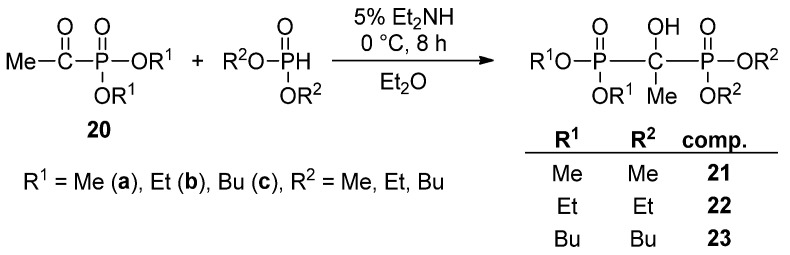
Pudovik reaction of dialkyl α-oxoethylphosphonates **20a**–**c** with dialkyl phosphites.

Based on our previous experiments, α-oxoethylphosphonates (**20**) were reacted with dialkyl phosphites in the presence of 5% diethylamine at 0 °C in diethyl ether for 8 h (Scheme 10). After purification by chromatography, symmetric hydroxy-methylenebisphosphonates (**21**–**23**) were obtained in yields of 68–86% [14].

**Scheme 11:**
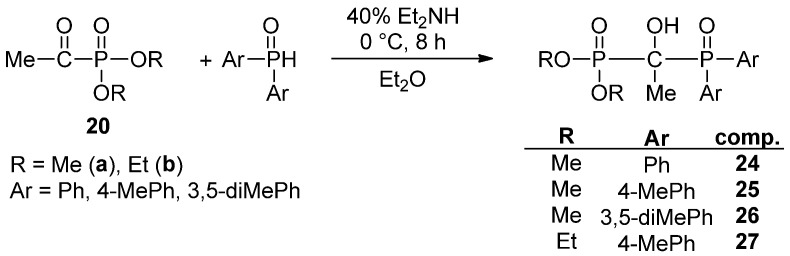
Pudovik reaction of dimethyl and diethyl α-oxoethylphosphonates **20a** and **20b** with diarylphosphine oxides.

We also investigated the Pudovik reaction of dimethyl and diethyl α-oxoethylphosphonates (**20a** and **20b**) with secondary phosphine oxides, such as diphenylphosphine oxide, bis(4-methylphenyl)phosphine oxide and bis(3,5-dimethylphenyl)phosphine oxide. In these cases, the reactions were carried out at 0 °C for 8 h using 40% of diethylamine as the catalyst (Scheme 11). Products **24**–**27** crystallized out from the mixture, and they were purified by recrystallization to give asymmetric phosphonate–phosphine oxide derivatives (**24**–**27**) in yields of 62–78% [14].

**Scheme 12:**
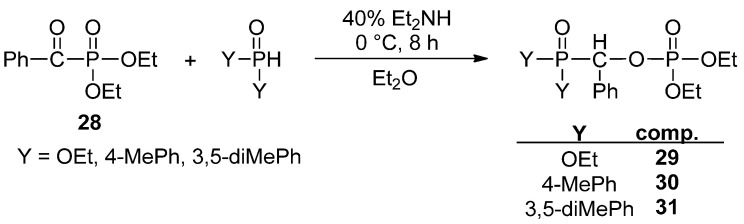
Synthesis of the rearranged derivatives **29**–**31**.

Finally, diethyl α-oxobenzylphosphonate (**28**) was reacted with diethyl phosphite, bis(4-methylphenyl)phosphine oxide and bis(3,5-dimethylphenyl)phosphine oxide in the presence of 40% diethylamine at 0 °C for 8 h (Scheme 12). The rearranged products (**29**–**31**) were obtained in yields of 65–74% after purification by chromatography [14].

Bisphosphonic derivatives **21**, **22**, **24**–**26**, **29**, **30** and **31** were described earlier [14], and now confirmed by their δ_P_ shifts and LC-MS analysis, while species **23** and **27** are new, and were characterized by ^31^P, ^13^C and ^1^H NMR spectroscopy, as well as by HRMS.

### 2.2. Bioactivity Study

**Figure 7:**
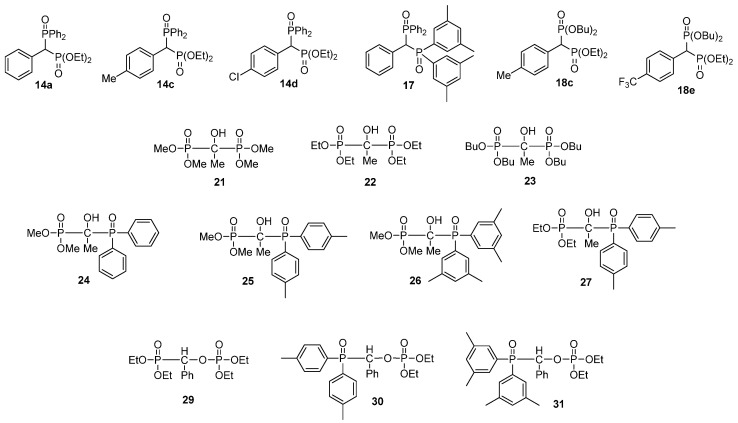
The library of bisphosphonic derivatives subjected to a bioactivity study.

Bisphosphonic products coming from the Michaelis–Arbuzov reaction **14a**, **14c**, **14d**, **17, 18c** and **18e**, along with hydroxy-methylenebisphosphonic derivatives (**21**–**27**) obtained in the Pudovik reaction, as well as related rearranged species **29**, **30** and **31**, were subjected to a bioactivity study (Figure 7).

Viability assays were performed on human cancer cell lines PANC-1 (pancreatic ductal adenocarcinoma) and U266 (multiple myeloma) at concentrations of 1, 10 and 100 µM. This aimed to evaluate the structure–activity relationship and aid in selecting compounds for further investigations. The screening results are summarized in Table 5.

First, clodronic acid disodium salt, used as a reference in our studies, was tested. This compound belongs to the first generation of the non-aminobisphosphonates [25] and has been used in the treatment of bone-related diseases [26], e.g., against multiple myeloma-related bone loss. It was also found that bisphosphonates may also have antiproliferative activity [27,28]. This effect further enhances their significance in the treatment of different malignant diseases. Interestingly, it had no antiproliferative effect on any of the cells in our long-term experiments. Under the same experimental conditions in our prior long-term experiments, bortezomib, a clinically significant compound used in the treatment of multiple myeloma, reduced U266 cell viability to 50% at a concentration of just 1.5 nM [29]. Tamoxifen reduced PANC-1 cell viability to 20% at 100 µM [30].

Among the 16 investigated, newly synthesized molecules, there were three compounds (**18e**, **23** and **31**) that could reduce the cell viability of PANC-1 cells to 80% or less. However, on U266 cells, six compounds (**14d**, **23**, **25**, **27**, **30** and **31**) had similar effects. It is important to mention that, in all cases, the highest investigated concentration of the compounds (100 µM) had remarkable effects on these two cell lines.

Our findings indicate that the U266 myeloma cell line demonstrates greater sensitivity to a larger number of the bisphosphonates tested as compared to the PANC-1 pancreatic ductal adenocarcinoma cell line. However, the lowest cell viability values were observed in PANC-1 cells, specifically with the compounds **18e** and **31**, as suggested by the viability of 0.31 and 0.43, respectively, after a 100 µM treatment. Historically, bisphosphonates have been used to treat myeloma-associated bone disease, with some limited anti-myeloma activity [25]. Unfortunately, their clinical application has its limitations due to potential adverse effects, including upper gastrointestinal side effects, acute phase reaction, or hypocalcemia [31], so their use is only recommended for 2 years according to the guidelines [32].

Our results suggest that bisphosphonates may have therapeutic potential beyond myeloma-associated bone disease, potentially serving as treatments for other malignancies, such as pancreatic ductal adenocarcinoma. This highlights the need for continued research for novel bisphosphonates that offer an improved benefit–risk profile for broader therapeutic applications.

#### Structure–Activity Relationships

Bisphosphonate derivatives lacking hydroxyl groups exhibit varying effects on cell viability depending on two main factors: (i) the nature of the phosphonate substitution (e.g., diethyl dibutyl or diethyl diphosphinoyl pairs) and (ii) the para-substitution of the benzene ring (e.g., methyl, chloro, or trifluoromethyl groups). The phosphonate–phosphine oxide compound **14a**, lacking a substituent in the benzene ring, had a minimal effect on cell viability. Among the phosphonate–phosphine oxide derivatives, **14c**, with a para-methyl substituent, reduced U266 myeloma cell viability to 0.83 ± 0.03 at a concentration of 100 μM. Replacing the methyl group with a chloro substituent (as in **14d**) enhanced the anti-myeloma activity of the compound, lowering cell viability to 0.56 ± 0.11. Interestingly, these phosphonate–phosphine oxide derivatives showed limited effectiveness on PANC-1 cells. A significant reduction in cell viability (0.31 ± 0.11) was only observed upon treatment with **18e** that is (a diethyl dibutyl bisphosphonate with a para-trifluoromethyl substitution) at 100 μM. The change of the trifluoromethyl group for a Me substituent, as in compound **18c**, resulted in negligible effects on PANC-1 cells.

Hydroxyl-containing derivatives were categorized into two main subgroups: (i) derivatives lacking an aromatic ring attached to the phosphonate group (e.g., **21**, **22** and **23**), and (ii) derivatives with two aromatic rings linked to the phosphonate group (e.g., **24**, **25**, **26** and **27**). Species **23**, a tetrabutyl bisphosphonate, exhibited similar antiproliferative effects on both cell lines at 100 μM (PANC-1: 0.73 ± 0.09 and U266: 0.75 ± 0.02). Further modifications involving benzene ring substitution on the phosphonate group did not enhance effectiveness against PANC-1 cells, however, significant effects were observed in U266 cells, with **25** and **26** reducing the cell viability to 0.76 ± 0.01 and 0.61 ± 0.01, respectively.

Additional bisphosphonate derivatives obtained by rearrangement (e.g., **29**, **30** and **31**) were also evaluated. Derivatives lacking aromatic units on the *P* atom (**29**) demonstrated no significant effects either on PANC-1 or U266 cells. Conversely, the benzene-containing derivatives **30** and **31** affected U266 cells, reducing cell viability to 0.80 ± 0.07 and 0.76 ± 0.02, respectively. Notably, compound **31** showed a substantial antiproliferative effect on PANC-1 cells, reducing viability to 0.43 ± 0.01.

## 3. Experimental

### 3.1. General Information

The ^31^P, ^13^C, ^1^H NMR spectra were taken on a Bruker DRX-500 spectrometer operating at 202, 126 and 500 MHz, respectively (Bruker, Billerica, MA, USA). The copies of the ^31^P, ^13^C and ^1^H NMR spectra for compounds **8e**, **9-11**, **14a-d**, **15-17**, **18a**, **18c**, **18e**, **19**, **23**, and **27** prepared can be seen in the Supplementary Materials. The couplings were given in Hz. LC–MS measurements were performed with an Agilent 1200 liquid chromatography system, coupled with a 6130 quadrupole mass spectrometer equipped with an ESI ion source (Agilent Technologies, Santa Clara, CA, USA). High-resolution mass spectrometric measurements were performed using a Thermo Velos Pro Orbitrap Elite hybrid mass spectrometer in positive electrospray mode (Thermo Fisher Scientific, Waltham, MA, USA).

### 3.2. General Procedure for the Synthesis of Diethyl α-Hydroxy-benzylphosphonates (**2a**–**e**)

A mixture of 11.0 mmol of aromatic aldehyde (**1a**: 1.2 g, **1b**: 1.5 g, **1c**: 1.3 g, **1d**: 1.5 g, **1e**: 1.9 g), 11.0 mmol of diethyl phosphite (1.4 mL) and 1.1 mmol (0.15 mL) of triethylamine were stirred in of 1 mL acetone at reflux. After 2 h, 6 mL of pentane was added to the reaction mixture, and it was cooled to 5 °C. Compounds **2a** and **2c**–**e** crystallized out from the reaction mixture. The crystals were filtered off and washed with pentane. Product **2b** was obtained as oil, and it was purified by column chromatography on silica gel applying CH_2_Cl_2_:MeOH 97:3 as the eluent. Products **2a** and **2c**–**e** are white crystalline compounds, and hydroxyphosphonate **2b** is a colorless oil. The data for identifying the products (**2a**–**e**) are listed in Table 6.

### 3.3. General Procedure for the Synthesis of Diaryl α-Hydroxy-benzylphosphine Oxides (**3**–**5**)

A mixture of 11.0 mmol of benzaldehyde (1.2 g) and 11.0 mmol of diarylphosphine oxide (diphenylphosphine oxide 1.1 g, bis(4-methylphenyl)phosphine oxide 1.3 g, bis(3,5-dimethylphenyl)phosphine oxide 14 g) and 1.1 mmol (0.15 mL) of triethylamine were stirred in 1 mL of acetone at reflux. After 1 h, 6 mL of pentane was added to the reaction mixture, and it was cooled to 5 °C. Compounds **3**, **4** and **5** crystallized out from the reaction mixture. The crystals were filtered off and washed with pentane. Hydroxyphosphine oxides **3**–**5** are white crystalline compounds. The data for identifying the products (**3**–**5**) are listed in Table 7.

### 3.4. General Procedure for the Synthesis of Dialkyl α-Halogeno-benzylphosphonates (**6** and **7**)

A mixture of 4.4 mmol of SOX_2_ (thionyl chloride: 0.32 mL, thionyl bromide: 0.38 mL) and dichloromethane (5 mL) was added to a mixture of 4.0 mmol (0.98 g) of diethyl α-hydroxy-benzylphosphonate (**2a**) and dichloromethane (5 mL). Then, the contents of the flask were stirred at 40 °C for 8 h. Finally, the volatile components were evaporated to give the corresponding crude product as oils that were purified by column chromatography (using DCM–MeOH 97:3 as the eluent on silica gel). Products **6** and **7** are pale yellow oils.

#### 3.4.1. Diethyl α-Chloro-benzylphosphonate (**6**)

Yield: 0.87 g (83%); pale yellow oil; ^31^P {^1^H} NMR (202 MHz, CDCl_3_) δ 17.2; δ_P,lit._ [37] 18.3; [M + H]^+^ = 263.

#### 3.4.2. Diethyl α-Bromo-benzylphosphonate (**7**)

Yield: 0.99 g (81%); pale yellow oil; ^31^P {^1^H} NMR (202 MHz, CDCl_3_) δ 17.1; δ_P,lit._ [38] 17.5; [M + H]^+^ = 307.

### 3.5. General Procedure for the Synthesis of Diethyl α-Methanesulfonyloxy-benzylphosphonates (**8a**–**e**)

A mixture of 1.0 mmol of diethyl α-hydroxy-benzylphosphonate (**2a**: 0.24 g; **2b**: 0.27 g, **2c**: 0.26 g, **2d**: 0.28 g, **2e**: 0.31 g), 1.5 mmol (0.12 mL) of methanesulfonyl chloride and 1.5 mmol (0.21 mL) of triethylamine in 5 mL of toluene were stirred at room temperature for half an hour. The precipitated triethylamine hydrochloride salt was filtered off, the filtrate was concentrated under vacuum, and the crude product so obtained was purified by column chromatography (using DCM–MeOH 95:5 as the eluent on silica gel). Product **8a**, **8d** and **8e** are white crystalline compounds, species **8b** and **8c** are pale yellow or colorless oils.

#### 3.5.1. Diethyl α-Methanesulfonyloxy-benzylphosphonate (**8a**)

Yield: 0.26 g (80%); white crystals; mp = 72–73 °C; ^31^P {^1^H} NMR (202 MHz, CDCl_3_) δ 14.5; δ_P,lit._ [17] 14.5; [M + H]^+^ = 323; HRMS *m*/*z*: [M + Na]^+^ calculated for C_12_H_19_O_6_PSNa 345.0538; found 345.0536.

#### 3.5.2. Diethyl α-Methanesulfonyloxy-3-methoxybenzylphosphonate (**8b**)

Yield: 0.27 g (79%), pale yellow oil; ^31^P {^1^H} NMR (202 MHz, CDCl_3_) δ 14.5; δ_P,lit._ [17] 14.5; [M + H]^+^ = 353; HRMS *m*/*z*: [M + Na]^+^ calculated for C_13_H_21_O_7_PSNa 375.0643; found 375.0642.

#### 3.5.3. Diethyl α-Methanesulfonyloxy-4-methylbenzylphosphonate (**8c**)

Yield: 0.26 g (76%), colorless oil; ^31^P {^1^H} NMR (202 MHz, CDCl_3_) δ 14.6; δ_P,lit._ [17] 14.7; [M + H]^+^ = 337; HRMS *m*/*z* [M + Na]^+^ calculated for C_13_H_21_O_6_PSNa 359.0694; found 359.0694.

#### 3.5.4. Diethyl α-Methanesulfonyloxy-4-chlorobenzylphosphonate (**8d**)

Yield: 0.29 g (82%); white crystals; mp = 76–77 °C; ^31^P {^1^H} NMR (202 MHz, CDCl_3_) δ 14.1; δ_P,lit._ [17] 14.2; [M + H]^+^ = 357; HRMS *m*/*z* [M + Na]^+^ calculated for C_12_H_18_ClO_6_PSNa 379.0148; found 379.0143.

#### 3.5.5. Diethyl α-Methanesulfonyloxy-4-trifluoromethylbenzylphosphonate (**8e**)

Yield: 0.33 g (84%); white crystals; mp = 88–89 °C; ^31^P {^1^H} NMR (202 MHz, CDCl_3_) δ 13.8; ^13^C {^1^H} NMR (126 MHz, CDCl_3_) δ 16.2 and 16.3 (d, *J* = 5.7 Hz, CH_2_*C*H_3_), 39.5 (s, SCH_3_), 64.0 and 64.3 (d, *J* = 6.9 Hz, OCH_2_), 76.3 (d, *J* = 169.6 Hz, CH), 123.7 (q, *J* = 272.4 Hz, CF_3_), 125.7–125.8 (d, C_γ_), 128.2 (d, *J* = 5.4 Hz, C_β_), 131.5 (q, *J* = 32.7 Hz, C_δ_), 136.4 (bs, C_α_); ^1^H NMR (500 MHz, CDCl_3_) δ 1.29 and 1.34 (t, *J* = 7.1 Hz, 6H, CH_2_C*H*_3_), 3.01 (s, 3H, SCH_3_), 4.05–4.22 (m, 4H, OCH_2_), 5.82 (d, *J* = 15.4 Hz, 1H, CH), 7.66–7.72 (m, 4H, ArH); [M + H]^+^ = 391; HRMS *m*/*z* [M + H]^+^ calculated for C_13_H_19_F_3_O_6_PS 391.0587; found 391.0586.

### 3.6. General Procedure for the Synthesis of Diaryl α-Methanesulfonyloxy-benzylphosphine Oxides (**9**–**11**)

A mixture of 1.0 mmol of diaryl α-hydroxy-benzylphosphine oxide (**3**: 0.31 g, **4**: 0.34 g, **5**: 0.36 g), 1.5 mmol (0.12 mL) of methanesulfonyl chloride and 1.5 mmol (0.21 mL) of triethylamine in 5 mL of toluene were stirred at room temperature for half an hour. The precipitated triethylamine hydrochloride salt was filtered off, the filtrate was concentrated under vacuum, and the crude product so obtained was purified by column chromatography (using DCM–MeOH 95:5 as the eluent on silica gel). Hydroxyphosphine oxides **9**–**11** are white crystalline compounds.

#### 3.6.1. Diphenyl α-Methanesulfonyloxy-benzylphosphine Oxide (**9**)

Yield: 0.30 g (78%); white crystals; mp = 170–171 °C; ^31^P {^1^H} NMR (202 MHz, CDCl_3_) δ 27.5; ^13^C {^1^H} NMR (126 MHz, CDCl_3_) δ 39.7 (s, SCH_3_), 80.3 (d, *J* = 81.6 Hz, CH), 127.5 and 129.3 (d, *J* = 101.5 Hz, C_α_′), 128.5 and 128.7 (d, *J* = 4.1 Hz, C_γ_′), 128.62 (d, *J* = 1.5 Hz, C_γ_), 128.63 (d, *J* = 7.3 Hz, C_β_), 129.6 (d, *J* = 2.2 Hz, C_δ_), 131.1 (d, *J* = 1.4 Hz, C_α_), 131.7 and 132.4 (d, *J* = 9.2 Hz, C_β_′), 132.7 and 132.9 (d, *J* = 2.9 Hz, C_δ_′); ^1^H NMR (500 MHz, CDCl_3_) δ 2.62 (s, 3H, SCH_3_), 6.27 (d, *J* = 7.0 Hz, 1H, CH), 7.27–7.94 (m, 15H, ArH); [M + H]^+^ = 387; HRMS *m*/*z* [M + H]^+^ calculated for C_20_H_20_O_4_PS 387.0815; found 387.0805.

#### 3.6.2. Bis(4-Methylphenyl α-Methanesulfonyloxy-benzyl)phosphine Oxide (**10**)

Yield: 0.33 g (79%); white crystals; mp = 192–193 °C; ^31^P {^1^H} NMR (202 MHz, CDCl_3_) δ 28.0; ^13^C {^1^H} NMR (126 MHz, CDCl_3_) δ 21.7 (d, *J* = 11.3 Hz, ArCH_3_), 39.6 (s, SCH_3_), 80.5 (d, *J* = 81.4 Hz, CH), 124.4 and 126.2 (d, *J* = 104.0 Hz, C_α_′), 128.52 (s, C_γ_), 128.53 (d, *J* = 6.2 Hz, C_β_), 129.3 and 129.4 (s, C_γ_′), 129.5 (d, *J* = 2.2 Hz, C_δ_), 131.4 (d, *J* = 1.4 Hz, C_α_), 131.8 and 132.4 (d, *J* = 9.6 Hz, C_β_′), 143.2 and 143.4 (d, *J* = 2.8 Hz, C_δ_′); ^1^H NMR (500 MHz, CDCl_3_) δ 2.40 and 2.46 (s, 6H, ArCH_3_), 2.61 (s, 3H, SCH_3_), 6.23 (d, *J* = 7.1 Hz, 1H, CH), 7.25–7.79 (m, 13H, ArH); [M + H]^+^ = 415; HRMS *m*/*z* [M + H]^+^ calculated for C_22_H_24_O_4_PS 415.1128; found 415.1125.

#### 3.6.3. Bis(3,5-Dimethylphenyl α-Methanesulfonyloxy-benzyl)phosphine Oxide (**11**)

Yield: 0.43 g (98%); white crystals; mp = 230–231 °C; ^31^P {^1^H} NMR (202 MHz, CDCl_3_) δ 28.2; ^13^C {^1^H} NMR (126 MHz, CDCl_3_) δ 21.3 (d, *J* = 12.9 Hz, ArCH_3_), 39.6 (s, SCH_3_), 80.4 (d, *J* = 80.4 Hz, CH), 127.6 and 129.0 (d, *J* = 100.5 Hz, C_α_′), 128.4 (d, *J* = 1.7 Hz, C_γ_), 128.7 (d, *J* = 4.4 Hz, C_β_) 129.4 (d, *J* = 2.3 Hz, C_δ_), 131.4 (bs, C_α_), 131.8 and 132.4 (d, *J* = 9.6 Hz, C_β_′), 134.3 and 134.4 (d, *J* = 3.0 Hz, C_δ_′), 138.3 and 138.4 (d, *J* = 6.4 Hz, C_γ_′); ^1^H NMR (500 MHz, CDCl_3_) δ 2.30 and 2.39 (s, 12H, ArCH_3_), 2.62 (s, 3H, SCH_3_), 6.21 (d, *J* = 6.1 Hz, 1H, CH), 7.25–7.51 (m, 11H, ArH); [M + H]^+^ = 443; HRMS *m*/*z* [M + H]^+^ calculated for C_24_H_28_O_4_PS 443.1441; found 443.1430.

### 3.7. General Procedure for the Synthesis of Diethyl α-(Diphenylphosphinoyl)-benzylphosphonates (**14a**–**d**)

The mixture of 1.0 mmol of diethyl α-methanesulfonyloxy-benzylphosphonates (**8a**: 0.32 g, **8b**: 0.35 g, **8c**: 0.34 g, **8d**: 0.36 g) and 5.0 mmol (1.1 mL) of ethyl diphenylphosphinite was heated at 135 °C in a sealed tube under N_2_ atmosphere. After 1 day, the crude product was purified by column chromatography (using DCM–MeOH 97:3 as the eluent on silica gel). Products **14a**, **14c** and **14d** are white crystalline compounds, while species **14b** is colorless oils.

#### 3.7.1. Diethyl α-(Diphenylphosphinoyl)-benzylphosphonate (**14a**)

Yield: 0.37 g (86%); white crystals; mp = 158–159 °C; ^31^P {^1^H} NMR (202 MHz, CDCl_3_) δ_P1_ 18.7 and δ_P2_ 27.2 (d, *J* = 5.1 Hz); ^13^C {^1^H} NMR (126 MHz, CDCl_3_) δ 16.02 and 16.06 (d, *J* = 6.2 Hz, CH_2_*C*H_3_), 48.2 (dd, *J*_1_ = 131.2 Hz, *J*_2_ = 58.0 Hz, CH), 62.8 and 63.2 (d, *J* = 7.1 Hz, OCH_2_), 127.5 (bs, C_δ_), 128.1 and 128.3 (d, *J* = 12.3 Hz, C_β_), 128.4 (d, *J* = 3.1 Hz, C_γ_), 130.0 (bs, C_α_), 131.09 and 131.6 (d, *J* = 9.0 Hz, C_β_′), 131.14 (d, *J* = 3.9 Hz, C_γ_′), 131.3 and 131.7 (d, *J* = 2.8 Hz, C_δ_′), 132.6 (d, *J* = 101.7 Hz, C_α_′); ^1^H NMR (500 MHz, CDCl_3_) δ 1.02 and 1.06 (t, *J* = 7.1 Hz, 6H, CH_2_C*H_3_*), 3.79–4.02 (m, 4H, OCH_2_), 4.25 (dd, *J*_1_ = 24.9 Hz, *J*_2_ = 12.1 Hz, 1H, CH), 7.15–7.34, 7.45–7.60 and 8.01–8.05 (m, 15H, ArH), [M + H]^+^ = 429; HRMS *m*/*z* [M + Na]^+^ calculated for C_23_H_26_O_4_P_2_Na 451.1204; found 451.1204.

#### 3.7.2. Diethyl α-(Diphenylphosphinoyl)-3-methoxybenzylphosphonate (**14b**)

Yield: 0.39 g (79%); white crystals; mp = 130–131 °C; ^31^P {^1^H} NMR (202 MHz, CDCl_3_) δ_P1_ 18.6 and δ_P2_ 27.1 (d, *J* = 4.8 Hz); ^13^C {^1^H} NMR (126 MHz, CDCl_3_) δ 16.01 and 16.07 (d, *J* = 6.5 Hz, CH_2_*C*H_3_), 48.1 (dd, *J*_1_ = 131.0 Hz, *J*_2_ = 57.8 Hz, CH), 55.2 (s, OCH_3_), 62.8 and 63.2 (d, *J* = 7.0 Hz, OCH_2_), 114.0 (bs, C_γ_), 116.1 (bs, C_δ_), 123.7 (t, *J* = 6.0 Hz, C_α_), 128.0 and 128.3 (d, *J* = 12.1 Hz, C_β_), 129.2 (t, *J* = 2.1 Hz, C_γ_′), 131.1 and 131.6 (d, *J* = 9.0 Hz, C_β_′), 131.3 and 131.7 (d, *J* = 2.8 Hz, C_δ_′), 132.4 (d, *J* = 102.3 Hz, C_α_′), 159.3 (t, *J* = 1.7 Hz, C_γ_(OMe)); ^1^H NMR (500 MHz, CDCl_3_) δ 1.03 and 1.07 (t, *J* = 7.1 Hz, 6H, CH_2_C*H*_3_), 3.72 (s, 3H, OCH_3_), 3.82–4.07 (m, 4H, OCH_2_), 4.28 (dd, *J*_1_= 24.9 Hz, *J*_2_ = 12.7 Hz, 1H, CH), 6.72–6.74, 7.09–7.13, 7.29–7.39, 7.51–7.65 and 8.01–8.05 (m, 14H, ArH); [M + H]^+^ = 459; HRMS *m*/*z* [M + H]^+^ calculated for C_24_H_29_O_5_P_2_ 459.1485; found 459.1485.

#### 3.7.3. Diethyl α-(Diphenylphosphinoyl)-4-methylbenzylphosphonate (**14c**)

Yield: 0.32 g (72%); white crystals; mp = 182–183 °C; ^31^P {^1^H} NMR (202 MHz, CDCl_3_) δ_P1_ 19.0 and δ_P2_ 27.0 (d, *J* = 3.9 Hz); ^13^C {^1^H} NMR (126 MHz, CDCl_3_) δ 15.99 and 16.05 (d, *J* = 6.8 Hz, CH_2_*C*H_3_), 21.1 (s, ArCH_3_), 47.6 (dd, *J*_1_ = 131.5 Hz, *J*_2_ = 58.5 Hz, CH), 62.7 and 63.2 (d, *J* = 7.0 Hz, OCH_2_), 126.5 (bs, C_α_), 128.0 and 128.3 (d, *J* = 12.2 Hz, C_β_), 129.2 (t, *J* = 2.1 Hz, C_γ_), 130.9 (t, *J* = 5.8 Hz, C_β_′), 131.1 and 131.6 (d, *J* = 9.1 Hz, C_γ_′), 131.3 and 131.7 (d, *J* = 2.8 Hz, C_δ_′), 132.6 (d, *J* = 101.6 Hz, C_α_′), 137.2 (bs, C_δ_); ^1^H NMR (500 MHz, CDCl_3_) δ 1.01 and 1.06 (t, *J* = 7.1 Hz, 6H, CH_2_C*H_3_*), 2.26 (s, 3H, ArCH_3_), 3.77–3.98 (m, 4H, OCH_2_), 4.27 (dd, *J*_1_ = 25.1 Hz, *J*_2_ = 12.7 Hz, 1H, CH), 7.00–7.02, 7.30–7.38, 7.51–7.56, 7.60–7.63 and 8.00–8.04 (m, 14H, ArH), [M + H]^+^ = 443; HRMS *m*/*z* [M + H]^+^ calculated for C_24_H_29_O_4_P_2_ 443.1536; found 443.1548.

#### 3.7.4. Diethyl α-(Diphenylphosphinoyl)-4-chlorobenzylphosphonate (**14d**)

Yield: 0.32 g (70%); white crystals; mp = 174–175 °C; ^31^P {^1^H} NMR (202 MHz, CDCl_3_) δ_P1_ 18.3 and δ_P2_ 26.5 (d, *J* = 5.1 Hz); ^13^C {^1^H} NMR (126 MHz, CDCl_3_) δ 16.0 (d, *J* = 6.3 Hz, CH_2_*C*H_3_), 47.6 (dd, *J*_1_ = 131.4 Hz, *J*_2_ = 57.5 Hz, CH), 62.8 and 63.3 (d, *J* = 7.1 Hz, OCH_2_), 128.2 and 128.3 (d, *J* = 12.2 Hz, C_β_), 128.6 (t, *J* = 1.9 Hz, C_γ_), 130.9 (t, *J* = 8.6 Hz, C_β_′), 131.5 (d, *J* = 9.1 Hz, C_γ_′), 131.8 and 131.9 (d, *J* = 2.8 Hz, C_δ_′), 132.3 (t, *J* = 5.7 Hz, C_α_), 132.5 (d, *J* = 98.3 Hz, C_α_′), 133.7 (bs, C_δ_); ^1^H NMR (500 MHz, CDCl_3_) δ 1.05 and 1.07 (t, *J* = 6.1 Hz, 6H, CH_2_C*H_3_*), 3.81–4.03 (m, 4H, OCH_2_), 4.24 (dd, *J*_1_ = 24.8 Hz, *J*_2_ = 12.0 Hz, 1H, CH), 7.17–7.19, 7.29–7.32, 7.36–7.41, 7.51–7.61, 7.76–7.80 and 8.00–8.04 (m, 14H, ArH), [M + H]^+^ = 463; HRMS *m*/*z* [M + H]^+^ calculated for C_23_H_26_ClO_4_P_2_ 463.0990; found 463.0992.

### 3.8. General Procedure for the Synthesis of Diaryl α-(Diphenylphosphinoyl)-benzylphosphine Oxides (**15**–**17**)

The mixture of 1.0 mmol of diaryl α-methanesulfonyloxy-benzylphosphine oxides (**7**: 0.39 g, **8**: 0.41 g, **9**: 0.44 g) and 5.0 mmol (1.1 mL) of ethyl diphenylphosphinite was heated at 135 °C in a sealed tube under N_2_ atmosphere. After 3 days, the crude product was purified by column chromatography (using DCM–MeOH 97:3 as the eluent on silica gel). Products **15**–**17** are white crystalline compounds.

#### 3.8.1. Diphenyl-α-(Diphenylphosphinoyl)-benzylphosphine Oxide (**15**)

Yield: 0.38 g (78%); white crystals; mp = 325–326 °C; ^31^P {^1^H} NMR (202 MHz, CDCl_3_) δ 29.5 (s); ^13^C {^1^H} NMR (126 MHz, CDCl_3_) δ 51.0 (t, *J* = 55.9 Hz, CH), 127.4 (bs, C_δ_), 128.0 and 128.3 (d, *J* = 12.5 Hz, C_β_′), 128.04 (d, *J* = 5.2 Hz, C_β_), 129.4 (bs, C_α_), 130.7 (d, *J* = 101.4 Hz, C_α_′), 130.8 (bs, C_γ_), 131.2 and 131.3 (d, *J* = 9.4 Hz, C_γ_′), 131.6 and 131.7 (d, *J* = 2.5 Hz, C_δ_′); ^1^H NMR (500 MHz, CDCl_3_) δ 4.93 (t, *J* = 15.1 Hz, 1H, CH), 6.88–7.84 (m, 25H, ArH), [M + H]^+^ = 493; HRMS *m*/*z* [M + H]^+^ calculated for C_31_H_27_O_2_P_2_ 493.1481; found 493.1490.

#### 3.8.2. Bis(4-Methylphenyl-α-(Diphenylphosphinoyl)-benzyl)phosphine Oxide (**16**)

Yield: 0.47 g (91%); white crystals; mp = 268–269 °C; ^31^P {^1^H} NMR (202 MHz, CDCl_3_) δ_P1_ 28.0 and δ_P2_ 28.8 (bs); ^13^C {^1^H} NMR (126 MHz, CDCl_3_) δ 21.5 (d, *J* = 11.9 Hz, ArCH_3_), 52.3 (t, *J* = 55.1 Hz, CH), The aromatic range was rather complex between δ 127.0–129.4 and 130.4–132.8, 141.7 and 141.8 (d, *J* = 3.0 Hz, C_δ_(Me)); ^1^H NMR (500 MHz, CDCl_3_) δ 2.24 and 2.35 (s, 6H, ArCH_3_), 5.53 (t, *J* = 15.8 Hz, 1H, CH), 6.96–7.92 (m, 23H, ArH), [M + H]^+^ = 521; HRMS *m*/*z* [M + H]^+^ calculated for C_33_H_31_O_2_P_2_ 521.1794; found 521.1819.

#### 3.8.3. Bis(3,5-Dimethylphenyl-α-(Diphenylphosphinoyl)-benzyl)phosphine Oxide (**17**)

Yield: 0.54 g (98%); white crystals; mp = 274–275 °C; ^31^P {^1^H} NMR (202 MHz, CDCl_3_) δ_P1_ 28.3 and δ_P2_ 28.5 (bs); ^13^C {^1^H} NMR (126 MHz, CDCl_3_) δ 21.2 (d, *J* = 14.6 Hz, ArCH_3_), 52.1 (t, *J* = 53.9 Hz, CH), The aromatic range was rather complex between δ 127.1–129.1 and 130.3–133.3, 137.5 and 137.7 (d, *J* = 12.8 Hz, C_γ_(Me)); ^1^H NMR (500 MHz, CDCl_3_) δ 2.17 and 2.28 (s, 12H, ArCH_3_), 5.55 (t, *J* = 17.4 Hz, 1H, CH), 6.88–8.04 (m, 21H, ArH), [M + H]^+^ = 549; HRMS *m*/*z* [M + H]^+^ calculated for C_35_H_35_O_2_P_2_ 549.2107; found 549.2131.

### 3.9. General Procedure for the Synthesis of Diethyl-Dibutyl-(phenylmethylene)-bisphosphonates (**18a**, **18c** and **18e**)

The mixture of 1.0 mmol of diethyl α-methanesulfonyloxy-benzylphosphonates (**6a**: 0.32 g, **6c**: 0.34 g, **6e**: 0.39 g) and 5.0 mmol (1.4 mL) of tributyl phosphite was heated at 135 °C in a sealed tube under N_2_ atmosphere for 3 days. The crude product was purified by column chromatography (using DCM–MeOH 97:3 as the eluent on silica gel). Products **18a**, **18c** and **18e** are colorless oils.

#### 3.9.1. Diethyl-Dibutyl-(phenylmethylene)-bisphosphonate (**18a**)

Yield: 0.38 g (91%); colorless oil; ^31^P {^1^H} NMR (202 MHz, CDCl_3_) δ_P1_ 18.5 and δ_P2_ 18.7 (d, *J* = 3.9 Hz); ^13^C {^1^H} NMR (126 MHz, CDCl_3_) δ 13.6 (d, *J* = 4.8 Hz, CH_2_*C*H_3_), 16.2 and 16.3 (d, *J* = 6.1 Hz, CH_2_*C*H_3_), 18.6 (d, *J* = 9.7 Hz, *C*H_2_CH_3_), 32.4 and 32.5 (d, *J* = 6.1 Hz, OCH_2_*C*H_2_), 45.7 (t, *J* = 132.9 Hz, CH), 62.9 and 63.4 (d, *J* = 6.9 Hz, OCH_2_), 66.5 and 67.1 (d, *J* = 7.1 Hz, OCH_2_), 127.6 (t, *J* = 2.6 Hz, C_δ_), 128.5 (t, *J* = 2.1 Hz, C_γ_), 130.2 (t, *J* = 7.6 Hz, C_α_), 130.4 (t, *J* = 6.4 Hz, C_β_); ^1^H NMR (500 MHz, CDCl_3_) δ 0.86 and 0.91 (t, J = 7.4 Hz, 6H, CH_2_C*H*_3_), 1.18 and 1.30 (t, *J* = 7.1 Hz, 6H, CH_2_C*H*_3_), 1.25–1.30 and 1.32–1.39 (m, 4H, C*H*_2_CH_3_), 1.47–1.53 and 1.60–1.63 (m, 4H, OCH_2_C*H*_2_), 3.76 (t, *J* = 25.1 Hz, 1H, CH), 3.87–4.17 (m, 8H, OCH_2_), 7.30–7.50 (m, 5H, ArH), [M + H]^+^ = 421; HRMS *m*/*z* [M + H]^+^ calculated for C_19_H_35_O_6_P_2_ 421.1904; found 421.1909.

#### 3.9.2. Diethyl-Dibutyl-(4-methylphenylmethylene)-bisphosphonate (**18c**)

Yield: 0.29 g (66%); colorless oil; ^31^P {^1^H} NMR (202 MHz, CDCl_3_) δ_P1_ 18.7 and δ_P2_ 18.9 (d, *J* = 5.1 Hz); ^13^C {^1^H} NMR (126 MHz, CDCl_3_) δ 13.6 (d, *J* = 5.4 Hz, CH_2_*C*H_3_), 16.2 and 16.3 (d, *J* = 6.1 Hz, CH_2_*C*H_3_), 18.6 (d, *J* = 9.6 Hz, *C*H_2_CH_3_), 21.1 (s, ArCH_3_), 32.4 and 32.5 (d, *J* = 6.1 Hz, OCH_2_*C*H_2_), 45.2 (t, *J* = 133.1 Hz, CH), 62.8 and 63.4 (d, *J* = 6.8 Hz, OCH_2_), 66.5 and 67.0 (d, *J* = 7.1 Hz, OCH_2_), 126.9 (t, *J* = 7.9 Hz, C_α_), 129.2 (t, *J* = 2.2 Hz, C_γ_), 130.3 (t, *J* = 6.4 Hz, C_β_), 137.3 (t, *J* = 2.8 Hz, C_δ_); ^1^H NMR (500 MHz, CDCl_3_) δ 0.86 and 0.91 (t, *J* = 7.4 Hz, 6H, CH_2_C*H*_3_), 1.19 and 1.30 (t, *J* = 7.0 Hz, 6H, CH_2_C*H*_3_), 1.25–1.31 and 1.32–1.39 (m, 4H, C*H*_2_CH_3_), 1.47–1.53 and 1.59–1.64 (m, 4H, OCH_2_C*H*_2_), 2.35 (s, 3H, ArCH_3_), 3.72 (t, *J* = 25.1 Hz, 1H, CH), 3.86–4.17 (m, 8H, OCH_2_), 7.14–7.35 (m, 4H, ArH), [M + H]^+^ = 435; HRMS *m*/*z* [M + H]^+^ calculated for C_20_H_37_O_6_P_2_ 435.2060; found 435.2069.

#### 3.9.3. Diethyl-Dibutyl-(4-trifluoromethylphenylmethylene)-bisphosphonate (**18e**)

Yield: 0.39 g (80%); colorless oil; ^31^P {^1^H} NMR (202 MHz, CDCl_3_) δ_P1_ 17.6 and δ_P2_ 17.8 (bs); ^13^C {^1^H} NMR (126 MHz, CDCl_3_) δ 13.5 (d, *J* = 7.9 Hz, CH_2_*C*H_3_), 16.2 and 16.3 (d, *J* = 6.1 Hz, CH_2_*C*H_3_), 18.6 (d, *J* = 10.9 Hz, *C*H_2_CH_3_), 32.3 and 32.4 (d, *J* = 6.2 Hz, OCH_2_*C*H_2_), 45.7 (t, *J* = 132.6 Hz, CH), 63.1 and 63.5 (d, *J* = 6.8 Hz, OCH_2_), 66.7 and 67.1 (d, *J* = 7.0 Hz, OCH_2_), 124.0 (d, *J* = 271.8 Hz, CF_3_), 125.2–125.4 (m, C_γ_), 129.8 (q, *J*_1_ = 33.8 Hz, *J*_2_ = 33.0 Hz, C_δ_), 130.7 (t, *J* = 6.3 Hz, C_β_), 134.9 (t, *J* = 7.7 Hz, C_α_); ^1^H NMR (500 MHz, CDCl_3_) δ 0.86 and 0.91 (t, *J* = 7.4 Hz, 6H, CH_2_C*H*_3_), 1.21 and 1.31 (t, *J* = 7.1 Hz, 6H, CH_2_C*H*_3_), 1.23–1.28 and 1.32–1.41 (m, 4H, C*H*_2_CH_3_), 1.41–1.53 and 1.60–1.64 (m, 4H, OCH_2_C*H*_2_), 3.82 (t, *J* = 24.8 Hz, 1H, CH), 3.98–4.20 (m, 8H, OCH_2_), 7.35 (s, 4H, ArH), [M + H]^+^ = 489; HRMS *m*/*z* [M + H]^+^ calculated for C_20_H_34_F_3_O_6_P_2_ 489.1778; found 489.1781.

### 3.10. Synthesis of Dibutyl α-(Diphenylphosphinoyl)-benzylphosphonate (**19**)

A mixture of 1.0 mmol (0.39 g) of diphenyl α-methanesulfonyloxy-benzylphosphine oxide (**9**) and 5.0 mmol (1.4 mL) of tributyl phosphite was heated at 135 °C in a sealed tube under N_2_ atmosphere for 3 days. The crude product was purified by column chromatography (using DCM–MeOH 97:3 as the eluent on silica gel). The product (**19**) was obtained as white crystals.

Yield: 0.30 g (61%); mp = 91–92 °C; ^31^P {^1^H} NMR (202 MHz, CDCl_3_) δ_P1_ 18.7 and δ_P2_ 26.7 (d, *J* = 4.4 Hz); ^13^C {^1^H} NMR (126 MHz, CDCl_3_) δ 13.6 (d, *J* = 2.3 Hz, CH_2_*C*H_3_), 18.5 (d, *J* = 2.6 Hz, *C*H_2_CH_3_), 32.2 (t, *J* = 5.8 Hz, OCH_2_*C*H_2_), 48.2 (dd, *J*_1_ = 131.3 Hz, *J*_2_ = 58.0 Hz, CH), 66.4 and 67.0 (d, *J* = 7.2 Hz, OCH_2_), 127.5 (bs, C_δ_), 128.1 and 128.30 (d, *J* = 12.2 Hz, C_β_), 128.33 (d, *J* = 3.1 Hz, C_γ_), 129.9 (bs, C_α_), 131.07 and 131.6 (d, *J* = 8.9 Hz, C_β_′), 131.12 (d, *J* = 4.5 Hz, C_γ_′), 131.3 and 131.7 (d, *J* = 2.8 Hz, C_δ_′), 132.4 (d, *J* = 102.1 Hz, C_α_′); ^1^H NMR (500 MHz, CDCl_3_) δ 0.81 (t, *J* = 7.4 Hz, 6H, CH_2_C*H_3_*); 1.13–1.22 (m, 4H, C*H*_2_CH_3_), 1.32–1.38 (m, 4H, OCH_2_C*H*_2_), 3.68–3.97 (m, 4H, OCH_2_), 4.25 (dd, *J*_1_ = 25.0 Hz, *J*_2_ = 12.3 Hz, 1H, CH), 7.14–7.33, 7.40–7.59 and 8.00–8.04 (m, 15H, ArH), [M + H]^+^ = 485; HRMS *m*/*z* [M + H]^+^ calculated for C_27_H_35_O_4_P_2_ 485.2006; found 485.2012.

### 3.11. General Procedure for the Synthesis of Tetraalkyl α-Hydroxy-ethylidenebisphosphonates (**21**–**23**)

Dialkyl α-oxoethylphosphonate (2.2 mmol; **20a**: 0.33 g, **20b**: 0.39 g, **20c**: 0.52 g) was added dropwise to a mixture of dialkyl phosphite (2.2 mmol; dimethyl phosphite: 0.20 mL, diethyl phosphite: 0.30 mL, dibutyl phosphite: 0.43 mL) and 0.11 mmol (10 µL) of diethylamine in diethyl ether (10 mL) at 0 °C on stirring. After an 8-h reaction time, the solvent was evaporated, and the crude product so obtained was purified by column chromatography (using DCM–MeOH 97:3 as the eluent on silica gel).

#### 3.11.1. Tetramethyl α-Hydroxy-ethylidenebisphosphonate (**21**)

Yield: 0.40 g (68%); ^31^P {^1^H} NMR (202 MHz, CDCl_3_) δ 22.3; δ_P,lit._ [11] 22.0; [M + H]^+^ = 263; HRMS *m*/*z* [M + Na]^+^ calculated for C_6_H_16_O_7_P_2_Na 285.0269; found 285.0272.

#### 3.11.2. Tetraethyl α-Hydroxy-ethylidenebisphosphonate (**22**)

Yield: 0.57 g (82%); ^31^P {^1^H} NMR (202 MHz, CDCl_3_) δ 20.3; δ_P,lit._ [12] 20.8; [M + H]^+^ = 319; HRMS *m*/*z* [M + Na]^+^ calculated for C_10_H_24_O_7_P_2_Na 341.0895; found 341.0891.

#### 3.11.3. Tetrabutyl α-Hydroxy-ethylidenebisphosphonate (**23**)

Yield: 0.76 g (80%); ^31^P {^1^H} NMR (202 MHz, CDCl_3_) δ 20.3; ^13^C {^1^H} NMR (126 MHz, CDCl_3_) δ 13.6 (s, CH_2_*C*H_3_), 18.6 (d, *J* = 1.86 Hz, *C*H_2_CH_3_), 20.0 (t, *J* = 2.2 Hz, C*C*H_3_), 32.6 (s, OCH_2_*C*H_2_), 67.2–67.3 (m, OCH_2_), 71.6 (t, *J* = 154.9 Hz, CP_2_); ^1^H NMR (500 MHz, CDCl_3_) δ 0.96 (t, *J* = 7.4 Hz, 12H, CH_2_C*H*_3_), 1.40–1.47 (m, 8H, C*H*_2_CH_3_), 1.63–1.73 (m, 11H, OCH_2_C*H*_2_ + CCH_3_), 2.54 (bs, 1H, OH), 4.14–4.24 (m, 8H, OCH_2_); [M + H]^+^ = 431; HRMS *m*/*z* [M + H]^+^ calculated for C_18_H_41_O_7_P_2_ 431.2323; found 431.2322.

### 3.12. General Procedure for the Synthesis of Dialkyl 1-Diarylphosphinoyl-1-hydroxy-ethylphosphonate (**24**–**27**)

Dialkyl α-oxoethylphosphonate (2.2 mmol; **20a**: 0.33 g, **20b**: 0.39 g) was added dropwise to a mixture of diarylphosphine oxide (2.2 mmol; diphenylphosphine oxide: 0.44 g, bis(4-methylphenyl)phosphine oxide: 0.50 g, bis(3,5-dimethylphenyl)phosphine oxide: 0.56 g) and diethylamine (0.88 mmol; 0.09 mL) in diethyl ether (13 mL) at 0 °C on stirring. After an 8-h reaction time, the precipitated material was removed by filtration, washed with diethyl ether, and the residue recrystallized from acetone. The products are white crystalline compounds.

#### 3.12.1. Dimethyl 1-Diphenylphosphinoyl-1-hydroxy-ethylphosphonate (**24**)

Yield: 0.50 g (64%); mp = 131–132 °C; ^31^P {^1^H} NMR (202 MHz, CDCl_3_) [14] δ_P1_ 23.9 and δ_P2_ 29.0 (d, *J* = 25.4 Hz); [M + H]^+^ = 355; HRMS *m*/*z* [M + Na]^+^ calculated for C_16_H_20_O_5_P_2_Na 377.0684; found 377.0681.

#### 3.12.2. Dimethyl 1-Bis(4-methylphenyl)phosphinoyl-1-hydroxy-ethylphosphonate (**25**)

Yield: 0.52 g (62%); mp = 153–154 °C; ^31^P {^1^H} NMR (202 MHz, CDCl_3_) [14] δ_P1_ 24.0 and δ_P2_ 30.2 (d, *J* = 29.0 Hz); [M + H]^+^ = 383; HRMS *m*/*z* [M + Na]^+^ calculated for C_18_H_24_O_5_P_2_Na 405.0997; found 405.1003.

#### 3.12.3. Dimethyl 1-Bis(3,5-dimethylphenyl)phosphinoyl-1-hydroxy-ethylphosphonate (**26**)

Yield: 0.62 g (69%); mp = 161–162 °C; ^31^P {^1^H} NMR (202 MHz, CDCl_3_) [14] δ_P1_ 24.3 and δ_P2_ 30.0 (d, *J* = 29.0 Hz); [M + H]^+^ = 411; HRMS *m*/*z* [M + Na]^+^ calculated for C_20_H_28_O_5_P_2_Na 433.1310; found 433.1312.

#### 3.12.4. Diethyl 1-Bis(4-methylphenyl)phosphinoyl-1-hydroxy-ethylphosphonate (**27**)

Yield: 0.70 g (78%); mp = 146–147 °C; ^31^P {^1^H} NMR (202 MHz, CDCl_3_) δ_P1_ 21.7 and δ_P2_ 30.7 (d, *J* = 22.9 Hz); ^13^C {^1^H} NMR (126 MHz, CDCl_3_) δ 16.1 and 16.3 (d, *J* = 5.6 Hz, CH_2_*C*H_3_), 20.7 (s, C*C*H_3_), 21.6 (s, ArCH_3_), 63.5 and 63.6 (d, *J* = 7.4 Hz, OCH_2_), 74.2 (dd, *J*_1_ = 153.4 Hz, *J*_2_ = 74.9 Hz, CP_2_), 127.3 (d, *J* = 100.6 Hz, C_α_), 128.8 and 128.9 (d, *J* = 12.2 Hz, C_γ_), 132.4 and 132.7 (d, *J* = 9.1 Hz, C_β_), 142.1 and 142.3 (d, *J* = 3.0 Hz, C_δ_); ^1^H NMR (500 MHz, CDCl_3_) δ 1.14 and 1.23 (t, *J* = 7.1 Hz, 6H, CH_2_C*H*_3_), 1.62 (dd, *J*_1_ = 15.9 Hz, *J*_2_ = 14.7 Hz, 3H, CCH_3_), 1.83 (bs, 1H, OH), 2.40 (s, 6H, ArCH_3_), 3.86–4.28 (m, 4H, OCH_2_), 7.28–7.33 (m, 4H, ArH), 7.94 and 8.05 (dd, *J*_1_ = 11.0 Hz, *J*_2_ = 8.0 Hz, 4H, ArH_β_); [M + H]^+^ = 411; HRMS *m*/*z* [M + H]^+^ calculated for C_20_H_29_O_5_P_2_ 411.1485; found 411.1487.

### 3.13. Synthesis of Diethyl (Diethylphosphonoylbenzyl)phosphate (**29**)

Diethyl α-oxobenzylphosphonate (**28**) (1.7 mmol; 0.40 g) was added slowly to a mixture of diethyl phosphite (1.7 mmol; 0.20 mL) and diethylamine (0.09 mmol; 8 µL) in diethyl ether (13 mL) at 0 °C whilst being stirred. After an 8-h reaction time, the solvent was evaporated, and the product was obtained as colorless oil by purification via column chromatography (using silica gel and 3% MeOH in DCM as the eluent).

Yield: 0.48 g (74%); ^31^P {^1^H} NMR (202 MHz, CDCl_3_) [14] δ_P1_ −1.1 and δ_P2_ 16.7 (d, *J* = 34.9 Hz); [M + H]^+^ = 381; HRMS *m*/*z* [M + Na]^+^ calculated for C_15_H_26_O_7_P_2_Na 403.1051; found 403.1057.

### 3.14. General Procedure for Diethyl (Diarylphosphinoylbenzyl)phosphates (**30** and **31**)

Diethyl α-oxobenzylphosphonate (**28**) (1.5 mmol; 0.36 g) was added slowly to a mixture of diarylphosphine oxide (1.5 mmol; bis(4-methylphenyl)phosphine oxide: 0.35 g, bis(3,5-dimethylphenyl)phosphine oxide: 0.40 g) and diethylamine (0.60 mmol; 60 µL) in diethyl ether (13 mL) at 0 °C on stirring. After an 8-h reaction time, the solvent was evaporated, and the crude product so obtained was purified with column chromatography (using ethyl acetate as the eluent on silica gel).

#### 3.14.1. Diethyl 1-Bis((4-methylphenyl)phosphinoylbenzyl)phosphate (**30**)

Yield: 0.40 g (65%); ^31^P NMR (202 MHz, CDCl_3_) [14] δ_P1_ −1.3 and δ_P2_ 29.0 (*J* = 31.4 Hz); [M + H]^+^ = 473; HRMS *m*/*z* [M + Na]^+^ calculated for C_25_H_30_O_5_P_2_Na 495.1466; found 495.1467.

#### 3.14.2. Diethyl 1-Bis((3,5-dimethylphenyl)phosphinoylbenzyl)phosphate (**31**)

Yield: 0.42 g (72%); ^31^P NMR (202 MHz, CDCl_3_) [14] δ_P1_ −1.2 and δ_P2_ 29.1 (*J* = 30.9 Hz); [M + H]^+^ = 501; HRMS *m*/*z* [M + Na]^+^ calculated for C_27_H_34_O_5_P_2_Na 523.1779; found 523.1771.

### 3.15. Single X-Ray Experimental

Single crystals of sulfonyloxyphosphonate **8e** and sulfonyloxyphosphine oxide **11**, suitable for X-ray diffraction, were obtained by slow evaporation of acetone solution. The crystals were introduced into perfluorinated oil and a suitable single crystal was carefully mounted on the top of a thin glass wire. Data collection was performed with an Oxford Xcalibur 3 diffractometer equipped with a Spellman generator (50 kV, 40 mA) and a Kappa CCD detector, operating with Mo-K_α_ radiation (λ = 0.71071 Ǻ).

Data collection and data reduction were performed with the CrysAlisPro version 1.171.40.82a software [39]. Absorption correction using the multiscan method [39] was applied. The structures were solved with SHELXS-97 (1997 edition) [40], refined with SHELXL-97 [41], and finally checked using PLATON version 2023.1 [42]. The structure was depicted with the DIAMOND version 3.2i evaluation program [20]. Details of the data collection and structure refinement are summarized in Table 8.

CCDC-2410672 and CCDC-2410673 contain supplementary crystallographic data for these compounds. These data can be obtained free of charge from The Cambridge Crystallographic Data Centre via www.ccdc.cam.ac.uk/data_request/cif (accessed on 16 December 2024).

The selected bond lengths (Å), bond angles (°) and torsion angles (°) of mesyloxyphosphonate **8e** are listed in Tables S1–S3, while the data for mesyloxyphosphine oxide **11** are listed in Tables S4–S6 in the Supplementary Materials.

### 3.16. Bioactivity Experimental

#### 3.16.1. Cell Culturing

The two cell lines examined in this study were human myeloma (U266) and human pancreatic ductal adenocarcinoma (PANC-1), both obtained from the European Collection of Authenticated Cell Cultures (ECACC, Salisbury, UK). The PANC-1 cell line exhibits adherent growth properties (87092802 ECACC), whereas U266 cells (85051003 ECACC) grow in suspension. PANC-1 cells were maintained in a DMEM medium (Sigma Ltd., St. Louis, MO, USA), while U266 cells were cultured in RPMI 1640 (Sigma Ltd., St. Louis, MO, USA). In both media, supplements included 10% fetal bovine serum (number), 1% L-glutamine (Invitrogen Corporation, New York, NY, USA), and 1% penicillin/streptomycin (Invitrogen Corporation, New York, NY, USA).

#### 3.16.2. Cell Viability Assays

The tested compounds were dissolved in dimethyl sulfoxide (DMSO); AppliChem GmbH, Darmstadt, Germany) with a stock concentration of 10^−1^ M, keeping the DMSO concentration below 1% (*v*/*v*). Stock solutions were stored at −80 °C, with fresh solutions prepared for each experiment.

An xCELLigence system, a non-invasive impedimetric method, was utilized to assess the viability of PANC-1 cells following treatment. Adherent PANC-1 cells were seeded at a density of 10^5^ cells/mL in 96-well plates (E-Plate 96 PET; ACEA Biosciences, San Diego, CA, USA) with gold microelectrodes on the well bottom to detect impedance changes correlated with cell proliferation. A baseline reading was taken in cell-free medium before seeding. After an overnight incubation, cells were exposed to the compounds at final concentrations of 1, 10, and 100 μM, along with respective controls (medium and DMSO). Data collection was conducted using RTCA 2.0 software (Real-Time Cell Analyzer; ACEA Biosciences, San Diego, CA, USA).

Due to U266 cells’ suspension growth, the xCELLigence system could not be used, so the CellTiter-Glo Luminescent Cell Viability Assay (Promega, Madison, WI, USA) was applied instead. U266 cells were seeded in white-walled 96-well plates (Thermo Scientific, Waltham, MA, USA) at a density of 10^5^ cells/mL. Following overnight incubation, cells were treated with the test molecules at 1, 10 and 100 μM concentrations, as well as appropriate controls (medium and DMSO). After a 72-h incubation period, CellTiter-Glo Reagent was added to each well, and luminescence was measured using a Fluoroskan FL Microplate Fluorometer and Luminometer (Thermo Scientific, Waltham, MA, USA).

All experiments were conducted in triplicate, with results normalized to the untreated medium control and presented as mean ± SD.

## 4. Conclusions

A series of α-hydroxy-benzylphosphonates and -benzylphosphine oxides obtained by the Pudovik reaction of substituted benzaldehydes and >P(O)H reagents (diethyl phosphite and diarylphosphine oxides) was converted to the α-chloro-, α-bromo- and α-methanesulfonyloxy derivatives. One sulfonyloxyphosphonate and a sulfonyloxyphosphine oxide, as suggested by single-crystal X-ray measurements, formed chains in the crystalline solid phase. Then, a molecule library comprising bisphosphonates, bis(phosphine oxides) and phosphonate–phosphine oxides obtained in the Michaelis–Arbuzov reaction, and hydroxy-methylenebisphosphonates, along with their rearranged derivatives prepared by the Pudovik reaction of α-oxophosphonates and >P(O)H reagents, was made available. From among the 16 organophosphorus compounds with two P-functions, three resulted in a viability of less than 0.73, and two viabilities were as low as 0.43 and 0.31 on pancreatic adenocarcinoma cells. On multiple myeloma cells, six agents induced a viability lower than 0.80, the best value was 0.56. Tetrabutyl α-hydroxy-ethylidenebisphosphonate and bis(3,5-dimethylphenyl-phosphinoylbenzyl)-diehtylphosphate displayed a significant cytotoxic effect on both cell lines. In conclusion, the antiproliferative activity of bisphosphonic derivatives may be influenced by their structural features. Substitution in the benzene ring and the appearance of a hydroxy group on the α-carbon resulted in cell line-dependent effects.

## Figures and Tables

**Table 1:**
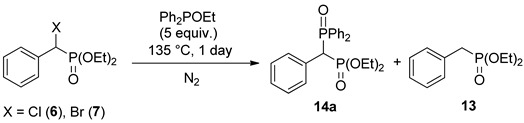
Michaelis–Arbuzov reaction of α-halogeno-benzylphosphonates (**6** and **7**) with ethyl diphenylphosphinite.

**Entry**	**Starting Material**		**Conversion (%) ^1^**	**Composition of the Mixture (%) ^1^**		
		**X**		**14a**	**13**	**Other Components**
1	**6**	Cl	90	65	25	Ph_2_P(O)Et, Ph_2_P(O)OEt
2	**7**	Br	94	48	46	Ph_2_P(O)Et, Ph_2_P(O)OEt

^1^ On the basis ^31^P NMR relative intensities.

**Table 2:**
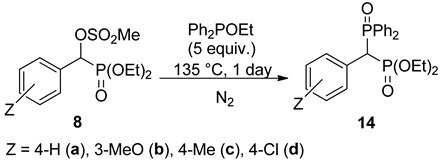
Michaelis–Arbuzov reaction of α-methanesulfonyloxy-benzylphosphonates (**8a**–**d**) with ethyl diphenylphosphinite.

**Entry**	**Starting Material**		**Product**	
		**Z**		**Yield (%)**
1	**8a**	4-H	**14a**	86
2	**8b**	3-MeO	**14b**	79
3	**8c**	4-Me	**14c**	72
4	**8d**	4-Cl	**14d**	70

**Table 3:**
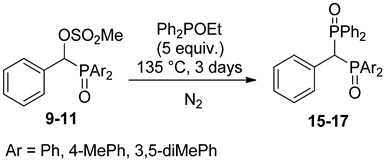
Michaelis–Arbuzov reaction of α-methanesulfonyloxy-benzylphosphine oxides (**9**–**11**) with ethyl diphenylphosphinite.

**Entry**	**Starting Material**		**Product**	
		**Ar**		**Yield (%)**
1	**9**	Ph	**15**	78
2	**10**	4-MePh	**16**	91
3	**11**	3,5-diMePh	**17**	98

**Table 4:**
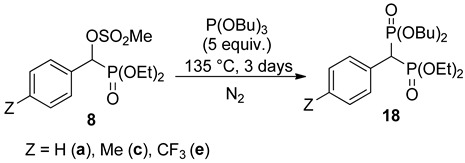
Michaelis–Arbuzov reaction of α-methanesulfonyloxy-benzylphosphonates (**8a**, **8c** and **8e**) with tributyl phosphite.

**Entry**	**Starting Material**		**Product**	
		**Z**		**Yield (%)**
1	**8a**	4-H	**18a**	91
2	**8c**	4-Me	**18c**	66
3	**8e**	4-CF_3_	**18e**	80

**Table 5:** In vitro antiproliferative effects of compounds (1, 10 and 100 µM) on PANC-1 and U266 cell lines after 72 h. Data represented as the mean ± SD ^1^; *n* = 3. The levels of significance are shown as follows: x: *p* < 0.05; y: *p* < 0.01; z: *p* < 0.001, determined by the one-way ANOVA test, followed by Fishers LSD post hoc test.

	PANC-1			U266		
Compound	1 µM	10 µM	100 µM	1 µM	10 µM	100 µM
Clodronate ^2^	1.02 ± 0.04	0.98 ± 0.03	0.98 ± 0.02	0.93 ± 0.05	0.89 ± 0.02 ^z^	0.88 ± 0.04 ^z^
**14a**	0.92 ± 0.05	0.88 ± 0.04	1.06 ± 0.08	1.02 ± 0.11	0.94 ± 0.03	0.90 ± 0.02
**14c**	0.94 ± 0.02	0.90 ± 0.12	1.04 ± 0.07	1.10 ± 0.07 ^y^	1.00 ± 0.01	0.83 ± 0.03 ^y^
**14d**	0.94 ± 0.01	0.92 ± 0.06	1.08 ± 0.07	1.02 ± 0.11	0.98 ± 0.02	0.56 ± 0.11 ^z^
**17**	0.95 ± 0.03	1.06 ± 0.11	1.00 ± 0.05	1.06 ± 0.06 ^x^	0.97 ± 0.03	0.96 ± 0.07
**18c**	0.96 ± 0.08	0.89 ± 0.07	1.06 ± 0.05	1.01 ± 0.12	0.97 ± 0.02	0.86 ± 0.06 ^x^
**18e**	1.04 ± 0.01	0.93 ± 0.24	0.31 ± 0.11 ^z^	1.12 ± 0.10 ^y^	0.94 ± 0.04	0.89 ± 0.03 ^x^
**21**	0.99 ± 0.05	0.91 ± 0.19	0.93 ± 0.06	0.93 ± 0.05	0.91 ± 0.02 ^y^	0.84 ± 0.02 ^z^
**22**	0.97 ± 0.04	1.00 ± 0.11	0.95 ± 0.09	0.90 ± 0.02 ^x^	0.87 ± 0.03 ^z^	0.84 ± 0.02 ^z^
**23**	0.83 ± 0.05	1.05 ± 0.04	0.73 ± 0.09 ^y^	0.92 ± 0.01	0.91 ± 0.03	0.75 ± 0.02 ^z^
**24**	1.21 ± 0.05	0.99 ± 0.05	1.01 ± 0.17	0.80 ± 0.19 ^z^	1.03 ± 0.03	0.94 ± 0.06
**25**	0.98 ± 0.25	1.19 ± 0.03	1.14 ± 0.14	0.94 ± 0.04	1.03 ± 0.04	0.76 ± 0.01 ^z^
**26**	1.04 ± 0.18	1.37 ± 0.31 ^x^	0.86 ± 0.11	0.90 ± 0.03	0.94 ± 0.07	0.61 ± 0.01 ^z^
**27**	1.22 ± 0.07 ^x^	1.22 ± 0.06	1.06 ± 0.15	0.89 ± 0.02 ^x^	0.99 ± 0.03	0.84 ± 0.01 ^y^
**29**	0.99 ± 0.04	0.94 ± 0.16	0.98 ± 0.10	0.95 ± 0.06	1.00 ± 0.03	0.91 ± 0.05 ^y^
**30**	0.94 ± 0.02	0.96 ± 0.19	0.99 ± 0.10	1.05 ± 0.03	0.94 ± 0.05	0.80 ± 0.07 ^z^
**31**	0.91 ± 0.04	0.99 ± 0.14	0.43 ± 0.01 ^z^	0.98 ± 0.04	0.93 ± 0.02 ^x^	0.76 ± 0.02 ^z^

^1^ SD: standard deviation; ^2^ reference compound. Pink: reduce cell viability of PANC-1 cells to 80% or less. Yellow: Reduce cell viability of U266 cells to 80% or less. Pale blue: Reduce cell viability of U266 cells to 85% or less.

**Table 6:** Preparation and identification of diethyl α-hydroxy-benzylphosphonates (**2a**–**e**).

Product	Z	Yield [%]	δ ^31^P (in CDCl_3_)	δ ^31^P_lit._ (in CDCl_3_)	[M + H]^+^	mp [°C]	Ref.
**2a**	H	87	21.4	21.7	245	83–84	[18]
**2b**	3-MeO	87	21.4	25.2	275	–	[33]
**2c**	4-Me	71	21.6	21.7	259	96–97	[34]
**2d**	4-Cl	74	20.9	21.0	279	74–75	[18]
**2e**	4-CF_3_	89	21.0	21.1	313	85–87	[35]

**Table 7:** Preparation and identification of diaryl α-hydroxy-benzylphosphine oxides (**3-5**).

Products	Ar	Yield [%]	δ ^31^P (in CDCl_3_)	δ ^31^P_lit._	[M + H]^+^	mp [°C]	Ref.
**3**	Ph	83	32.6	32.7 (in CDCl_3_)	309	74–75	[34]
**4**	4-MePh	79	34.3	28.1 (in DMSO-*d*_6_)	337	183–184	[36]
**5**	3,5-diMePh	76	34.1	28.1 (in DMSO-*d*_6_)	365	144–145	[36]

**Table 8:** Details for X-ray data collection and structure refinement for sulfonyloxyphosphonate **8e** and sulfonyloxyphosphine oxide **11**.

	8e	11
Empirical formula	C_13_H_18_F_3_O_6_PS	C_24_H_27_O_4_PS
Formula mass	390.30	442.48
T [K]	123(2)	123(2)
Crystal size [mm]	0.40 × 0.10 × 0.05	0.40 × 0.40 × 0.30
Crystal description	colorless rod	colorless block
Crystal system	monoclinic	orthorhombic
Space group	*P*21/*c*	*Pna*21
a [Ǻ]	19.4625(16)	12.1566(2)
b [Ǻ]	5.4101(4)	12.4348(2)
c [Ǻ]	16.4538(16)	14.9331(2)
α [°]	90.0	90.0
β [°]	99.465(9)	90.0
γ [°]	90.0	90.0
V [Ǻ^3^]	1708.9(3)	2257.36(6)
Z	4	4
ρ_calcd._ [g cm^−3^]	1.517	1.302
μ [mm^−1^]	0.340	0.242
*F*(000)	808	936
Θ range [°]	2.88–25.24	2.13–25.24
Index ranges	−24 ≤ *h* ≤ 24	−17 ≤ *h* ≤ 17
	−6 ≤ *k* ≤ 6	−17 ≤ *k* ≤ 17
	−20 ≤ *l* ≤ 20	−21 ≤ *l* ≤ 21
Reflns. collected	23,800	44,217
Reflns. obsd.	2074	6252
Reflns. unique	3369 (R_int_ = 0.1969)	6878 (R_int_ = 0.0346)
*R*_1_, *wR*_2_ (2σ data)	0.0753, 0.1564	0.0321, 0.0804
*R*_1_, *wR*_2_ (all data)	0.1278, 0.1857	0.0376, 0.0846
GOOF on *F*^2^	1.040	1.048
Peak/hole [e Ǻ^−3^]	0.525/−0.386	0.309/−0.201

## Data Availability

The data presented in this study are available on request from the corresponding author.

## Supplementary Materials

The following supporting information can be downloaded at: www.mdpi.com/article/10.3390/ph18010091/s1, X-ray data for sulfonyloxy derivatives **8e** and **11**; ^31^P, ^13^C and ^1^H NMR spectra for compounds **8e**, **9**–**11**, **14a**–**d**, **15**–**17**, **18a**, **18c**, **18e**, **19**, **23**, and **27** synthesized. Table S1: Selected bond lengths (Å) of mesyloxyphosphonate **8e**; Table S2: Selected bond angles (°) of mesyloxyphosphonate **8e**; Table S3: Selected torsion angles (°) of mesyloxyphosphonate **8e**; Table S4: Selected bond lengths (Å) of mesyloxyphosphine oxide **11**; Table S5: Selected bond angles (°) of mesyloxyphosphine oxide **11**; Table S6: Selected torsion angles (°) of mesyloxyphosphine oxide **11**.
