# Supplementary material for: Cytotoxic Activity of Bisphosphonic Derivatives Obtained by the Michaelis–Arbuzov or the Pudovik Reaction
Source: Pharmaceuticals (Basel). 2025 Jan 13;18(1):91. doi: 10.3390/ph18010091 (PMC11768586; doi:10.3390/ph18010091)

# Supplementary Materials

## Cytotoxic Activity of Bisphosphonic Derivatives Obtained by the Michaelis-Arbuzov or the Pudovik Reaction

**Zsuzsanna Szalai [1], Janka Bednárik [1], Boldizsár Szigfrid Tóth [1], Angéla Takács [2], Szilárd Tekula [2], László Kőhidai [2], Konstantin Karaghiosoff [3], László Drahos [4], György Keglevich [1]***

[1] *Department of Organic Chemistry and Technology, Faculty of Chemical Technology and Biotechnology, Budapest University of Technology and Economics, 1111 Budapest, Műegyetem rkp. 3., Hungary; szalai.zsuzsanna@edu.bme.hu (Z.S.); janka.bednarik@edu.bme.hu (J.B.); tothszigfridboldizsar@edu.bme.hu (B.S.T.); keglevich.gyorgy@vbk.bme.hu (G.K.)*

[2] *Department of Genetics, Cell-and Immunobiology, Semmelweis University, Nagyvárad tér 4, 1089 Budapest, Hungary; takacs.angela@semmelweis.hu (A.T.); tekula.szilard@stud.semmelweis.hu (S.T.); kohidai.laszlo@semmelweis.hu (L.K.)*

[3] *Department Chemie, Ludwig-Maximilians-Universität München, Butenandtstr. 5-13, D-81377 München, Germany, klk@cup.uni-muenchen.de (K.K.)*

[4] *MS Proteomics Research Group, Research Centre for Natural Sciences, 1117 Budapest, Hungary; drahos.laszlo@ttk.hu (L.D.)*

**Correspondence: keglevich.gyorgy@vbk.bme.hu (G.K.)*

## Table of Contents

1. Geometrical data for mesyloxyphosphonate **8e** and mesyloxyphosphine oxide **11** obtained from the X-ray measurements ........ S2

2. Spectra for the compounds **8e**, **9-11**, **14a-d**, **15-17**, **18a**, **18c**, **18e**, **19**, **23**, and **27** synthesized ........ S6

# 1. Geometrical data for mesyloxyphosphonate 8e and mesyloxyphosphine oxide 11 obtained from the X-ray measurements

**Table S1.** Selected bond lengths (Å) of mesyloxyphosphonate **8e**.

| | | | |
|---|---|---|---|
| P1 – O1 | 1.471(3) | C5 – C6 | 1.379(7) |
| P1 – O3 | 1.559(3) | C5 – C8 | 1.491(7) |
| P1 – O2 | 1.564(4) | C6 – C7 | 1.386(7) |
| P1 – C1 | 1.827(5) | C10 – C11 | 1.509(7) |
| F1 – C8 | 1.316(7) | C12 – C13 | 1.489(7) |
| C1 – O4 | 1.461(6) | F3 – C8 | 1.322(6) |
| C1 – C2 | 1.517(7) | O3 – C12 | 1.468(5) |
| F2 – C8 | 1.302(6) | C3 – C4 | 1.363(7) |
| S1 – O5 | 1.421(4) | C4 – C5 | 1.399(7) |
| S1 – O6 | 1.422(4) | C2 – C7 | 1.388(7) |
| S1 – O4 | 1.587(3) | C2 – C3 | 1.383(6) |
| S1 – C9 | 1.742(5) | O2 – C10 | 1.464(6) |

**Table S2.** Selected bond angles (°) of mesyloxyphosphonate **8e**.

| | | | |
|---|---|---|---|
| O1 – P1 – O3 | 113.4(2) | F2 – C8 – F1 | 106.5(5) |
| O1 – P1 – O2 | 116.6(2) | F2 – C8 – F3 | 105.5(6) |
| O3 – P1 – O2 | 104.9(2) | F1 – C8 – F3 | 103.7(5) |
| O1 – P1 – C1 | 110.6(2) | F2 – C8 – C5 | 113.2(5) |
| O3 – P1 – C1 | 106.4(2) | F1 – C8 – C5 | 113.4(5) |
| O2 – P1 – C1 | 104.0(2) | F3 – C8 – C5 | 113.7(5) |
| O4 – C1 – C2 | 111.4(4) | O2 – C10 – C11 | 111.0(4) |
| O4 – C1 – P1 | 103.9(3) | O3 – C12 – C13 | 107.8(4) |
| C2 – C1 – P1 | 111.3(3) | C7 – C2 – C1 | 118.5(4) |
| O5 – S1 – O6 | 120.1(2) | C12 – O3 – P1 | 123.8(3) |
| O5 – S1 – O4 | 103.6(2) | C4 – C3 – C2 | 121.1(5) |
| O6 – S1 – O4 | 108.9(2) | C1 – O4 – S1 | 118.7(3) |
| O5 – S1 – C9 | 109.8(2) | C3 – C4 – C5 | 119.5(5) |
| O6 – S1 – C9 | 109.0(2) | C6 – C5 – C4 | 119.9(5) |
| O4 – S1 – C9 | 104.1(2) | C6 – C5 – C8 | 121.3(5) |
| C10 – O2 – P1 | 122.1(3) | C4 – C5 – C8 | 118.8(5) |
| C3 – C2 – C7 | 119.7(5) | C5 – C6 – C7 | 120.2(5) |
| C3 – C2 – C1 | 121.7(4) | C6 – C7 – C2 | 119.6(5) |

**Table S3.** Selected torsion angles (°) of mesyloxyphosphonate **8e**.

| | | | |
|---|---|---|---|
| O1 – P1 – C1 – O4 | -58.5(3) | O5 – S1 – O4 – C1 | -166.7(3) |
| O3 – P1 – C1 – O4 | 65.1(3) | O6 – S1 – O4 – C1 | -37.7(4) |
| O2 – P1 – C1 – O4 | 175.6(3) | C9 – S1 – O4 – C1 | 78.5(4) |
| O1 – P1 – C1 – C2 | 61.5(4) | C2 – C3 – C4 – C5 | -1.0(8) |
| O3 – P1 – C1 – C2 | -174.9(3) | C3 – C4 – C5 – C6 | 0.5(8) |
| O2 – P1 – C1 – C2 | -64.4(4) | C3 – C4 – C5 – C8 | -178.4(5) |
| O1 – P1 – O2 – C10 | -24.3(4) | C4 – C5 – C6 – C7 | -0.1(8) |
| O3 – P1 – O2 – C10 | -150.7(3) | C8 – C5 – C6 – C7 | 178.8(5) |
| C1 – P1 – O2 – C10 | 97.7(4) | C5 – C6 – C7 – C2 | 0.1(8) |
| O4 – C1 – C2 – C3 | 38.5(6) | C3 – C2 – C7 – C6 | -0.5(7) |
| P1 – C1 – C2 – C3 | -76.9(5) | C1 – C2 – C7 – C6 | -176.1(5) |
| O4 – C1 – C2 – C7 | -146.0(4) | C6 – C5 – C8 – F2 | -107.7(7) |
| P1 – C1 – C2 – C7 | 98.5(5) | C4 – C5 – C8 – F2 | 71.2(7) |
| O1 – P1 – O3 – C12 | -158.1(4) | C6 – C5 – C8 – F1 | 130.9(6) |
| O2 – P1 – O3 – C12 | -29.8(4) | C4 – C5 – C8 – F1 | -50.2(7) |
| C1 – P1 – O3 – C12 | 80.1(4) | C6 – C5 – C8 – F3 | 12.7(8) |
| C7 – C2 – C3 – C4 | 1.0(8) | C4 – C5 – C8 – F3 | -168.4(5) |
| C1 – C2 – C3 – C4 | 176.4(5) | P1 – O2 – C10 – C11 | 85.9(5) |
| C2 – C1 – O4 – S1 | 83.7(4) | P1 – O3 – C12 – C13 | -159.0(4) |
| P1 – C1 – O4 – S1 | -156.4(2) | | |

**Table S4.** Selected bond lengths (Å) of mesyloxyphosphine oxide **11**.

| | | | |
|---|---|---|---|
| S1 – O4 | 1.421(2) | C18 – C19A | 1.502(3) |
| S1 – O3 | 1.424(2) | C19A – C20A | 1.381(4) |
| S1 – O2 | 1.587(2) | C19A – C24A | 1.456(10) |
| S1 – C25 | 1.759(3) | C20A – C21A | 1.443(12) |
| P1 – O1 | 1.488(1) | C21A – C22A | 1.356(13) |
| P1 – C15 | 1.796(2) | C22A – C23A | 1.359(10) |
| P1 – C1 | 1.800(2) | C23A – C24A | 1.371(11) |
| P1 – C18 | 1.846(2) | C12 – C13 | 1.391(3) |
| C1 – C2 | 1.394(3) | C13 – C14 | 1.393(3) |
| C1 – C6 | 1.402(3) | C13 – C17 | 1.505(3) |
| O2 – C18 | 1.464(2) | C14 – C15 | 1.397(3) |
| C2 – C3 | 1.388(3) | C10 – C11 | 1.393(3) |
| C3 – C4 | 1.395(3) | C10 – C15 | 1.399(2) |

| | | | |
|---|---|---|---|
| C3 – C7 | 1.508(3) | C11 – C12 | 1.395(3) |
| C4 – C5 | 1.389(3) | C11 – C16 | 1.502(3) |
| C5 – C6 | 1.394(3) | C5 – C8 | 1.514(3) |

**Table S5.** Selected bond angles (°) of mesyloxyphosphine oxide **11**.

| | | | |
|---|---|---|---|
| O4 – S1 – O3 | 120.3(1) | O2 – C18 – C19A | 112.0(2) |
| O4 – S1 – O2 | 109.2(1) | O2 – C18 – P1 | 103.5(1) |
| O3 – S1 – O2 | 104.6(1) | C19A – C18 – P1 | 112.9(1) |
| O4 – S1 – C25 | 109.0(1) | C20A – C19A – C24A | 125.9(5) |
| O3 – S1 – C25 | 108.7(1) | C20A – C19A – C18 | 121.3(2) |
| O2 – S1 – C25 | 103.7(1) | C24A – C19A – C18 | 112.8(5) |
| O1 – P1 – C15 | 113.0(1) | C19A – C20A – C21A | 110.6(7) |
| O1 – P1 – C1 | 113.4(1) | C22A – C21A – C20A | 125.5(7) |
| C15 – P1 – C1 | 105.9(1) | C21A – C22A – C23A | 120.6(7) |
| O1 – P1 – C18 | 113.8(1) | C22A – C23A – C24A | 120.6(7) |
| C15 – P1 – C18 | 105.0(1) | C23A – C24A – C19A | 116.8(6) |
| C1 – P1 – C18 | 105.03(9) | C12 – C13 – C14 | 118.7(2) |
| C2 – C1 – C6 | 119.9(2) | C12 – C13 – C17 | 121.4(2) |
| C2 – C1 – P1 | 116.5(2) | C14 – C13 – C17 | 119.9(2) |
| C6 – C1 – P1 | 123.5(2) | C13 – C14 – C15 | 120.0(2) |
| C18 – O2 – S1 | 119.5(1) | C14 – C15 – C10 | 120.5(2) |
| C3 – C2 – C1 | 120.6(2) | C14 – C15 – P1 | 122.0(1) |
| C2 – C3 – C4 | 118.7(2) | C10 – C15 – P1 | 117.4(1) |
| C2 – C3 – C7 | 120.7(2) | C11 – C10 – C15 | 120.1(2) |
| C4 – C3 – C7 | 120.6(2) | C10 – C11 – C12 | 118.4(2) |
| C5 – C4 – C3 | 122.0(2) | C10 – C11 – C16 | 121.2(2) |
| C4 – C5 – C6 | 118.7(2) | C12 – C11 – C16 | 120.4(2) |
| C4 – C5 – C8 | 120.7(2) | C13 – C12 – C11 | 122.4(2) |
| C6 – C5 – C8 | 120.6(2) | C5 – C6 – C1 | 120.1(2) |

**Table S6.** Selected torsion angles (°) of mesyloxyphosphine oxide **11**.

| | | | |
|---|---|---|---|
| O1 – P1 – C1 – C2 | 23.1(2) | C18 – P1 – C15 – C10 | -127.9(2) |
| C15 – P1 – C1 – C2 | -101.3(2) | S1 – O2 – C18 – C19A | -85.7(2) |
| C18 – P1 – C1 – C2 | 147.9(2) | S1 – O2 – C18 – P1 | 152.5(1) |
| O1 – P1 – C1 – C6 | -161.3(2) | O1 – P1 – C18 – O2 | 54.7(2) |
| C15 – P1 – C1 – C6 | 74.3(2) | C15 – P1 – C18 – O2 | 178.7(1) |
| C18 – P1 – C1 – C6 | -36.4(2) | C1 – P1 – C18 – O2 | -69.9(1) |

| | | | |
|---|---|---|---|
| O4 – S1 – O2 – C18 | -26.8(2) | O1 – P1 – C18 – C19A | -66.5(2) |
| O3 – S1 – O2 – C18 | -156.8(2) | C15 – P1 – C18 – C19A | 57.5(2) |
| C25 – S1 – O2 – C18 | 89.3(2) | C1 – P1 – C18 – C19A | 168.9(2) |
| C6 – C1 – C2 – C3 | -0.3(3) | O2 – C18 – C19A – C20A | -48.2(3) |
| P1 – C1 – C2 – C3 | 175.5(2) | P1 – C18 – C19A – C20A | 68.1(3) |
| C1 – C2 – C3 – C4 | 0.5(4) | O2 – C18 – C19A – C24A | 131.1(4) |
| C1 – C2 – C3 – C7 | 179.5(3) | P1 – C18 – C19A – C24A | -112.7(4) |
| C2 – C3 – C4 – C5 | -0.1(4) | C24A – C19A – C20A – C21A | 1.3(7) |
| C7 – C3 – C4 – C5 | -179.1(3) | C18 – C19A – C20A – C21A | -179.5(4) |
| C3 – C4 – C5 – C6 | -0.5(4) | C19A – C20A – C21A – C22A | -0.8(10) |
| C3 – C4 – C5 – C8 | 179.1(2) | C20A – C21A – C22A – C23A | 0.7(14) |
| C4 – C5 – C6 – C1 | 0.7(3) | C21A – C22A – C23A – C24A | -1.0(14) |
| C8 – C5 – C6 – C1 | -178.9(2) | C22A – C23A – C24A – C19A | 1.4(13) |
| C2 – C1 – C6 – C5 | -0.3(3) | C20A – C19A – C24A – C23A | -1.7(10) |
| P1 – C1 – C6 – C5 | -175.8(2) | C18 – C19A – C24A – C23A | 179.1(6) |
| C15 – C10 – C11 – C12 | 0.4(3) | C13 – C14 – C15 – P1 | 175.9(2) |
| C15 – C10 – C11 – C16 | -178.8(2) | C11 – C10 – C15 – C14 | 0.1(3) |
| C10 – C11 – C12 – C13 | -0.4(3) | C11 – C10 – C15 – P1 | -176.5(2) |
| C16 – C11 – C12 – C13 | 178.8(2) | O1 – P1 – C15 – C14 | -179.9(2) |
| C11 – C12 – C13 – C14 | -0.1(3) | C1 – P1 – C15 – C14 | -55.3(2) |
| C11 – C12 – C13 – C17 | 179.2(2) | C18 – P1 – C15 – C14 | 55.6(2) |
| C12 – C13 – C14 – C15 | 0.6(3) | O1 – P1 – C15 – C10 | -3.4(2) |
| C17 – C13 – C14 – C15 | -178.7(2) | C1 – P1 – C15 – C10 | 121.3(2) |
| C13 – C14 – C15 – C10 | -0.6(3) | | |

## 2. Spectra for the compounds 8e, 9-11, 14a-d, 15-17, 18a, 18c, 18e, 19, 23, and 27 synthesized

### $^{31}P$ {$^{1}H$} NMR (202 MHz, $CDCl_3$) spectra for 8e

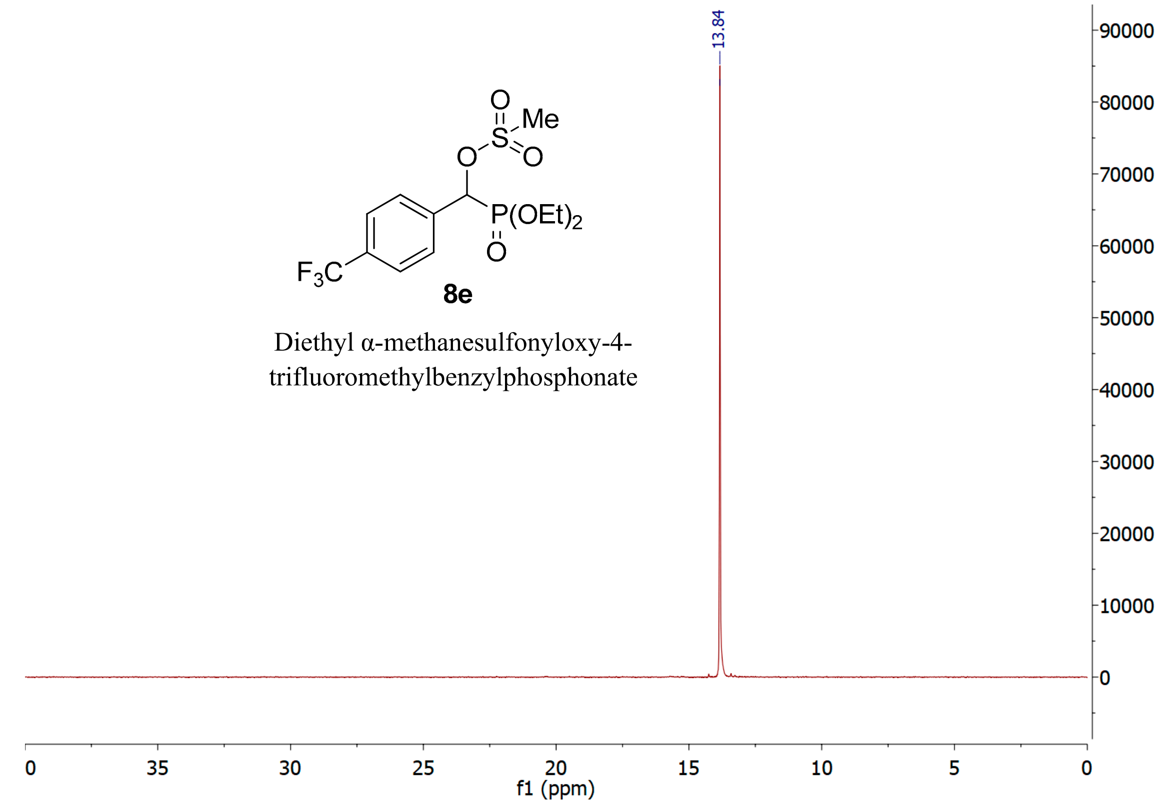


### $^{13}C$ {$^{1}H$} NMR (126 MHz, $CDCl_3$) spectra for 8e

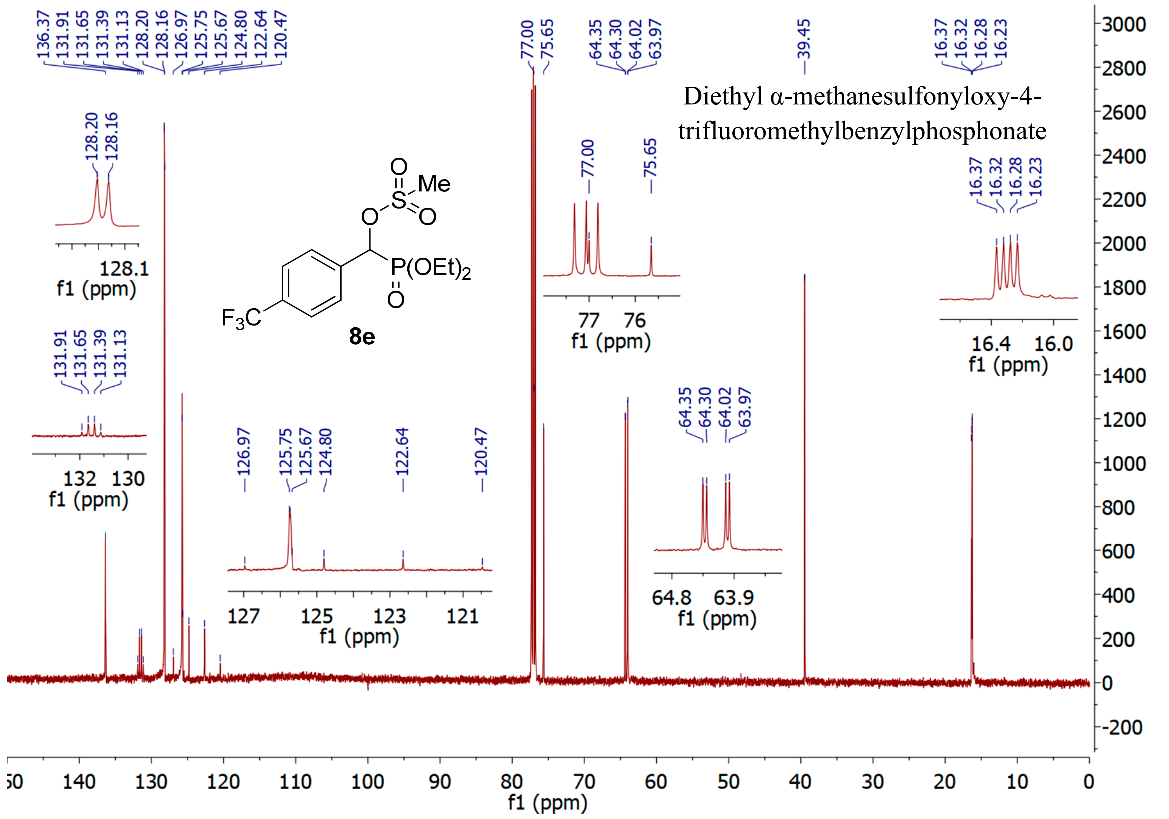

**$^{1}$H NMR (500 MHz, CDCl₃) spectra for 8e**

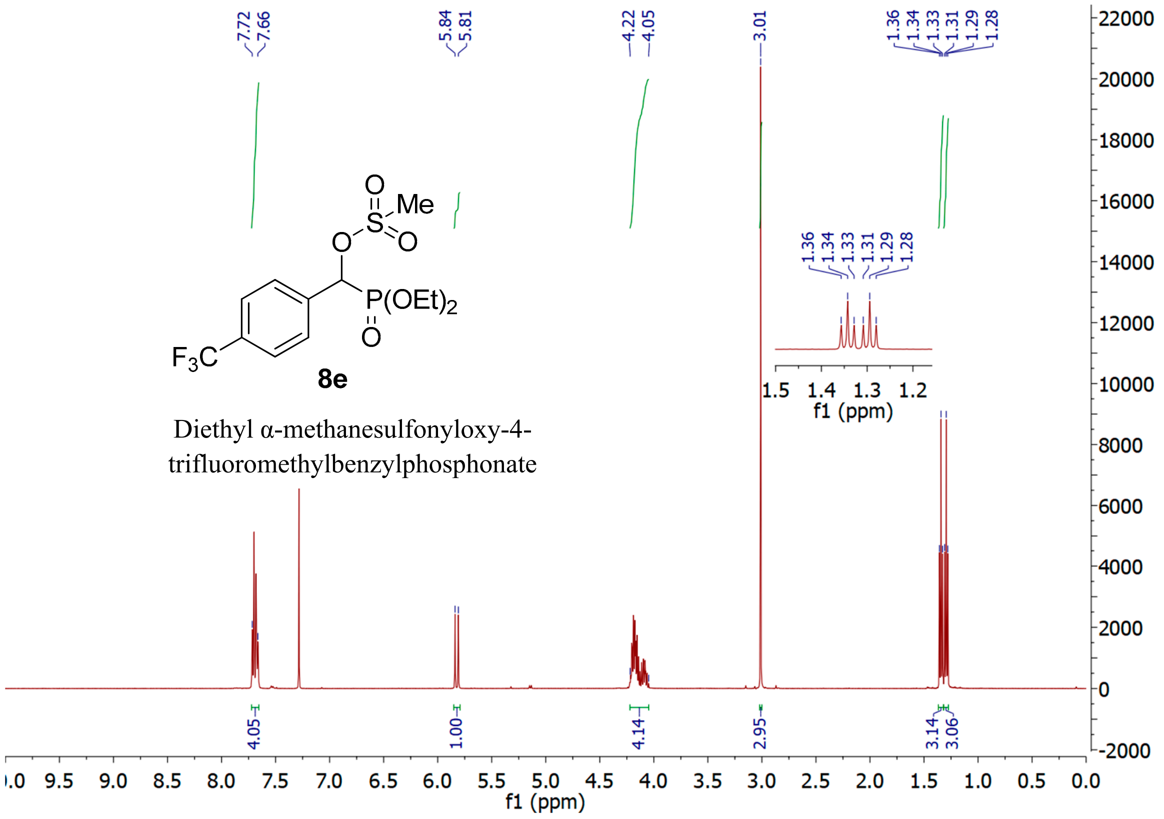


**$^{31}$P {$^{1}$H} NMR (202 MHz, CDCl₃) spectra for 9**

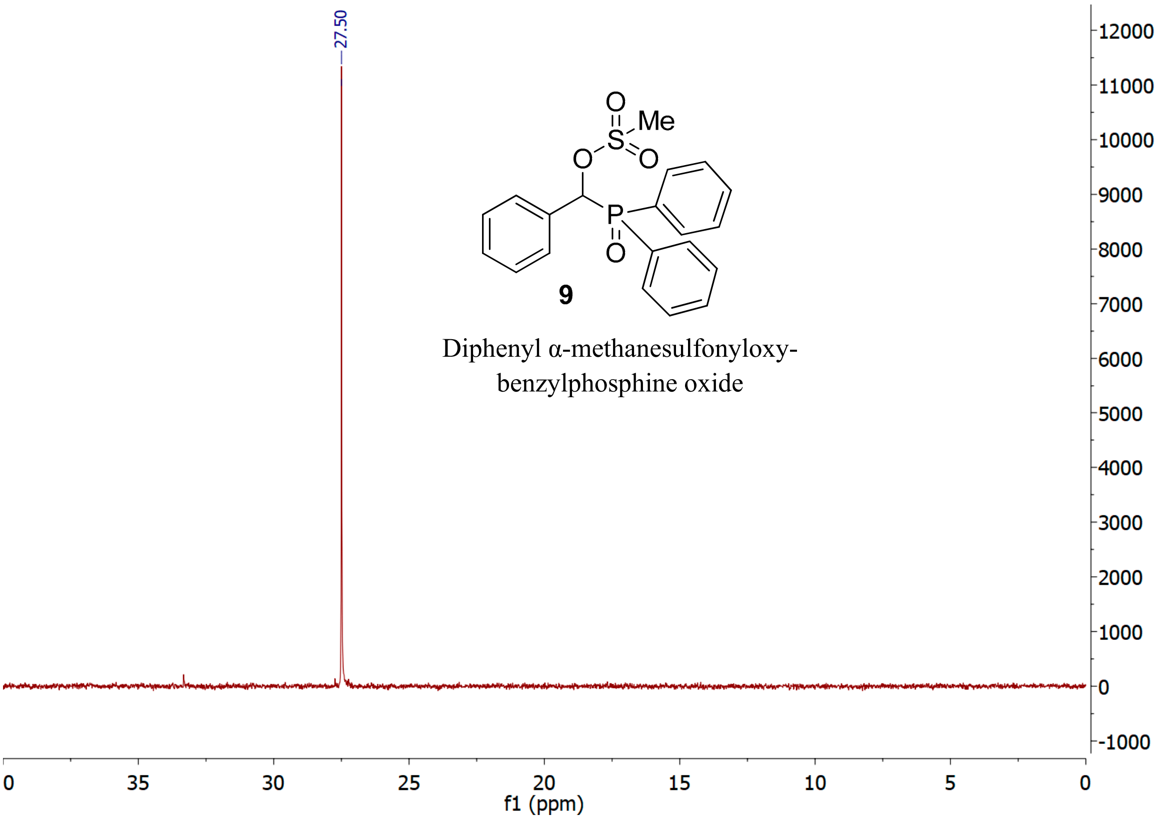

**$^{13}$C {$^{1}$H} NMR (126 MHz, $CDCl_3$) spectra for 9**

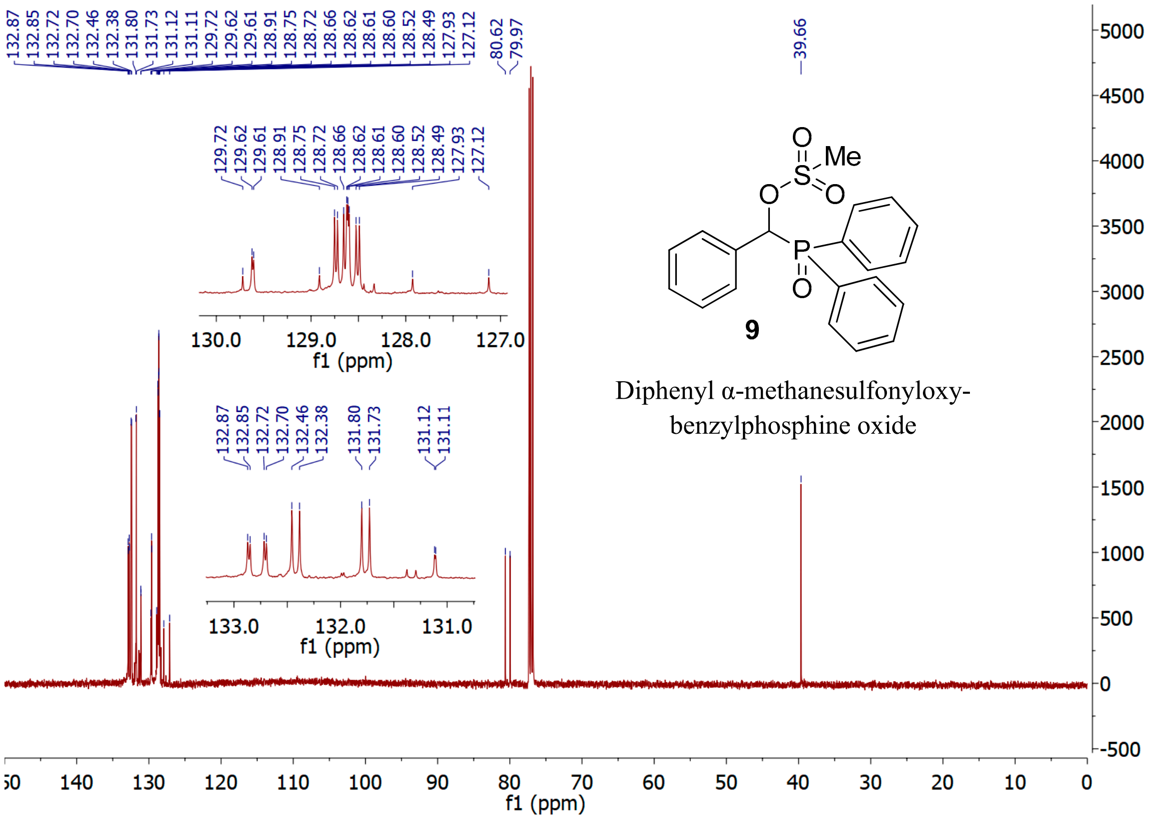


**$^{1}$H NMR (500 MHz, $CDCl_3$) spectra for 9**

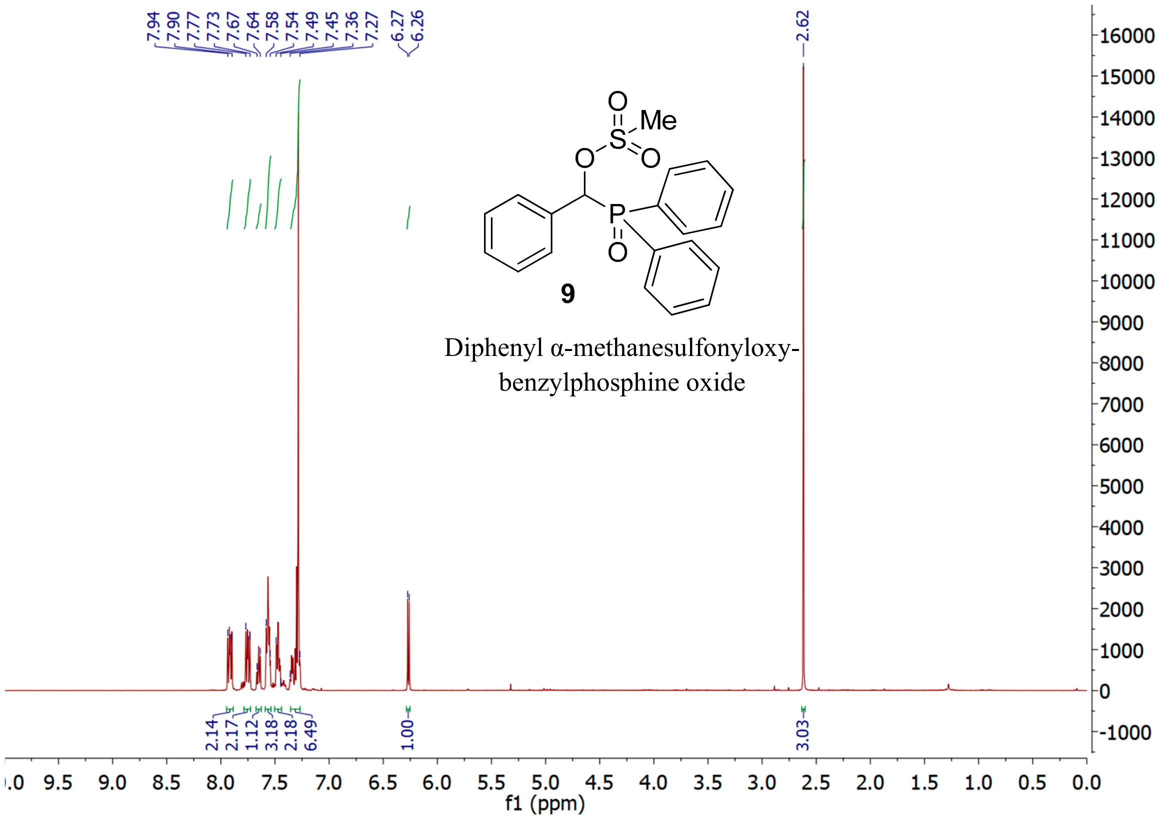

## $^{31}P\{^1H\}$ NMR (202 MHz, $CDCl_3$) spectra for 10

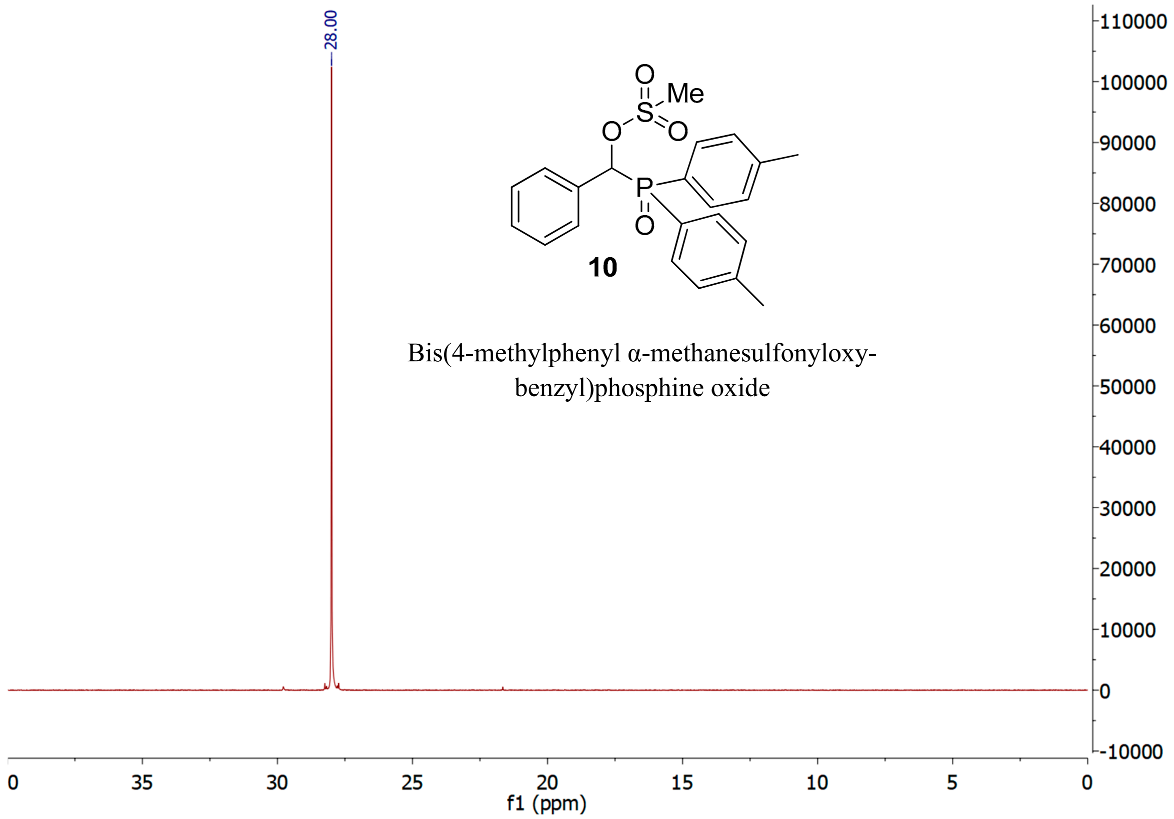


## $^{13}C\{^1H\}$ NMR (126 MHz, $CDCl_3$) spectra for 10

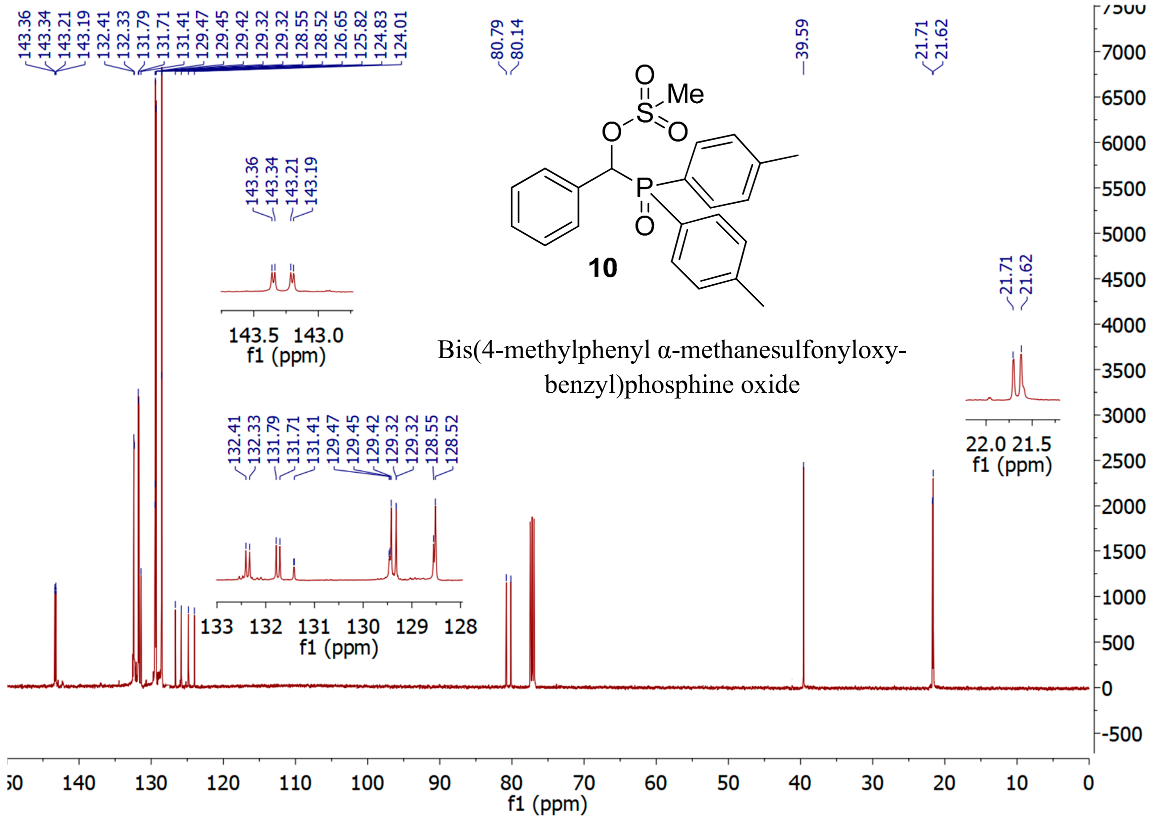

## $^{1}$H NMR (500 MHz, $CDCl_3$) spectra for 10

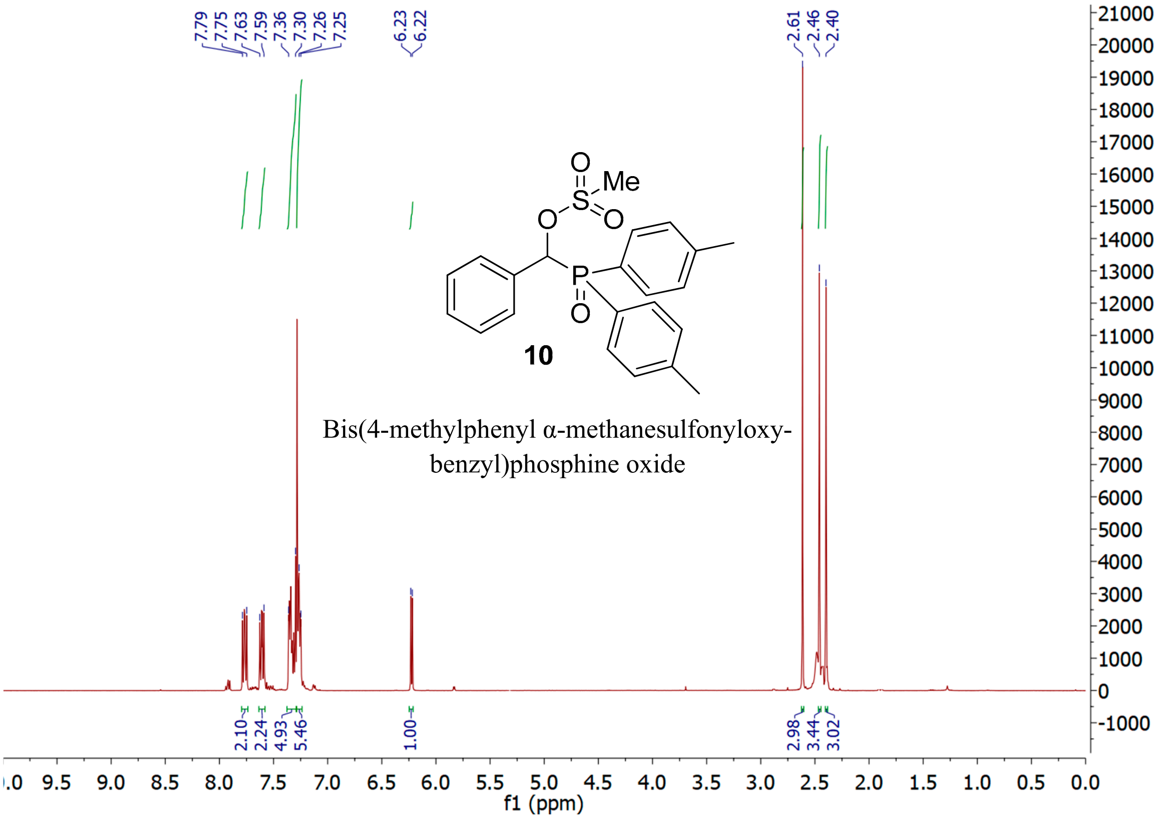


## $^{31}$P {$^{1}$H} NMR (202 MHz, $CDCl_3$) spectra for 11

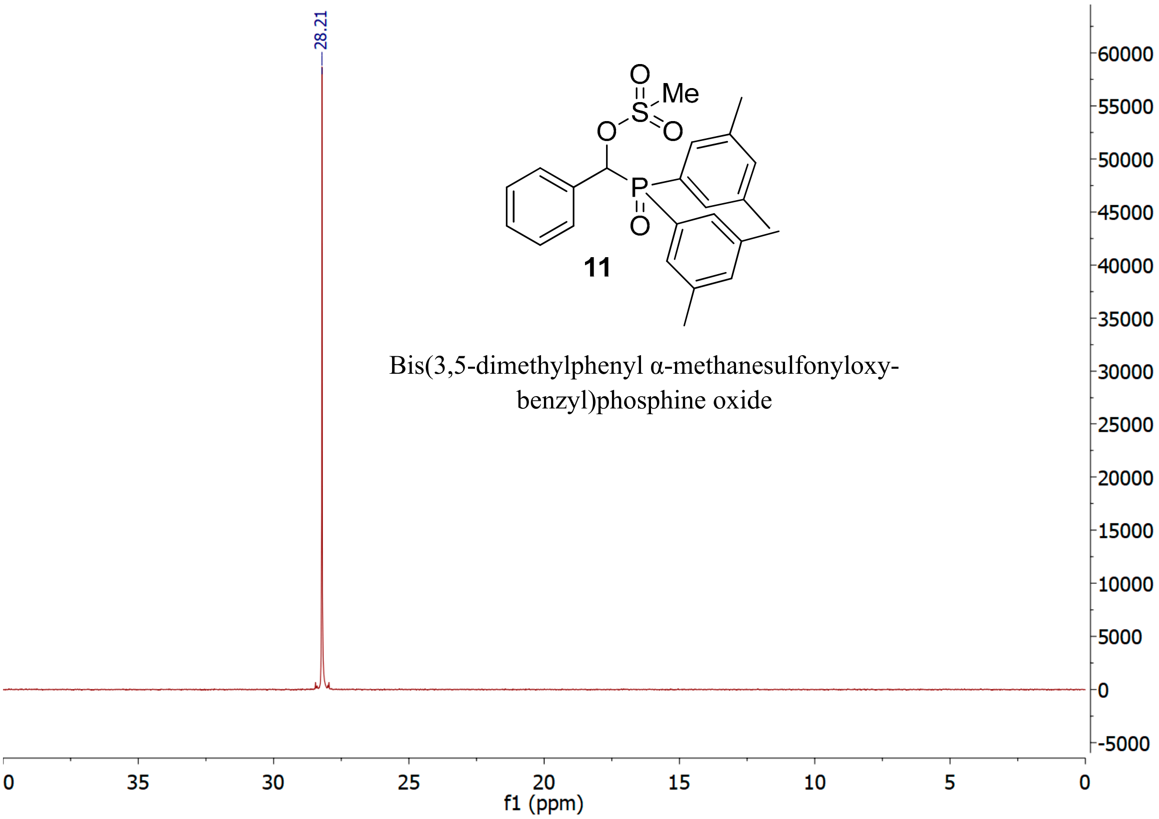

## $^{13}C$ {$^{1}H$} NMR (126 MHz, $CDCl_3$) spectra for 11

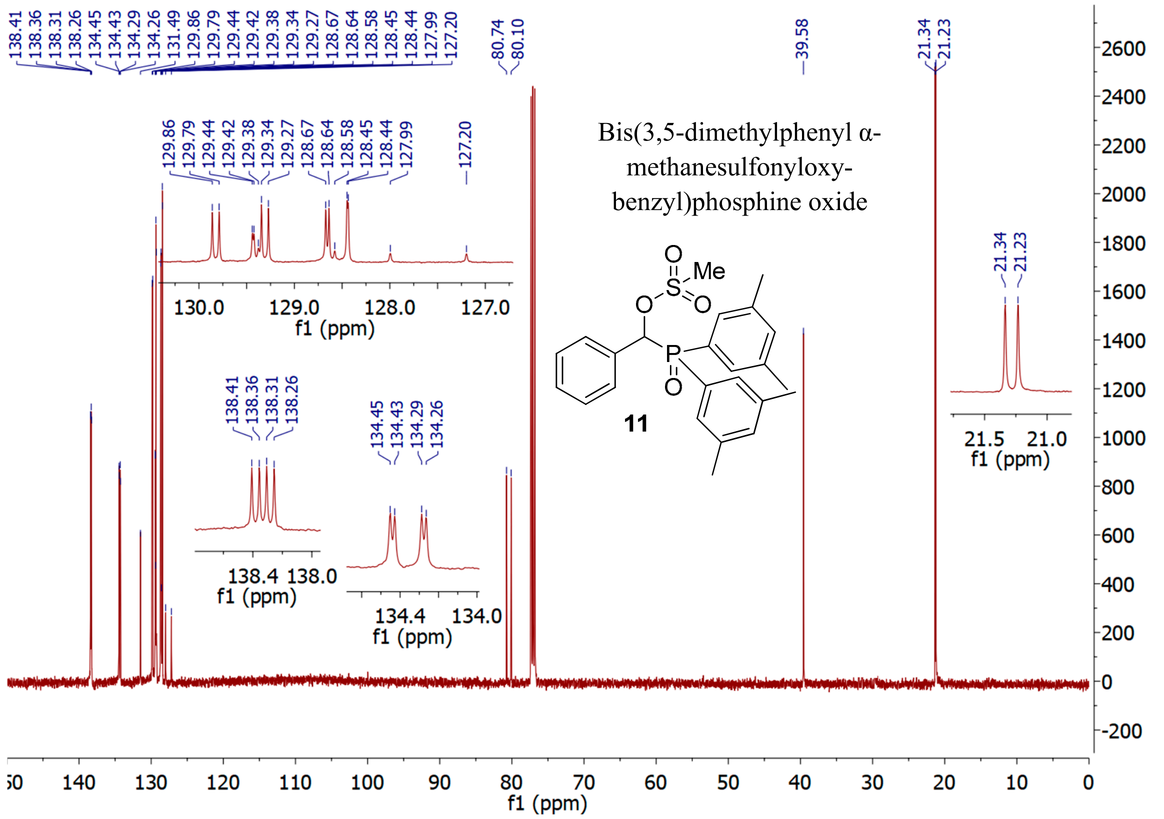


## $^{1}H$ NMR (500 MHz, $CDCl_3$) spectra for 11

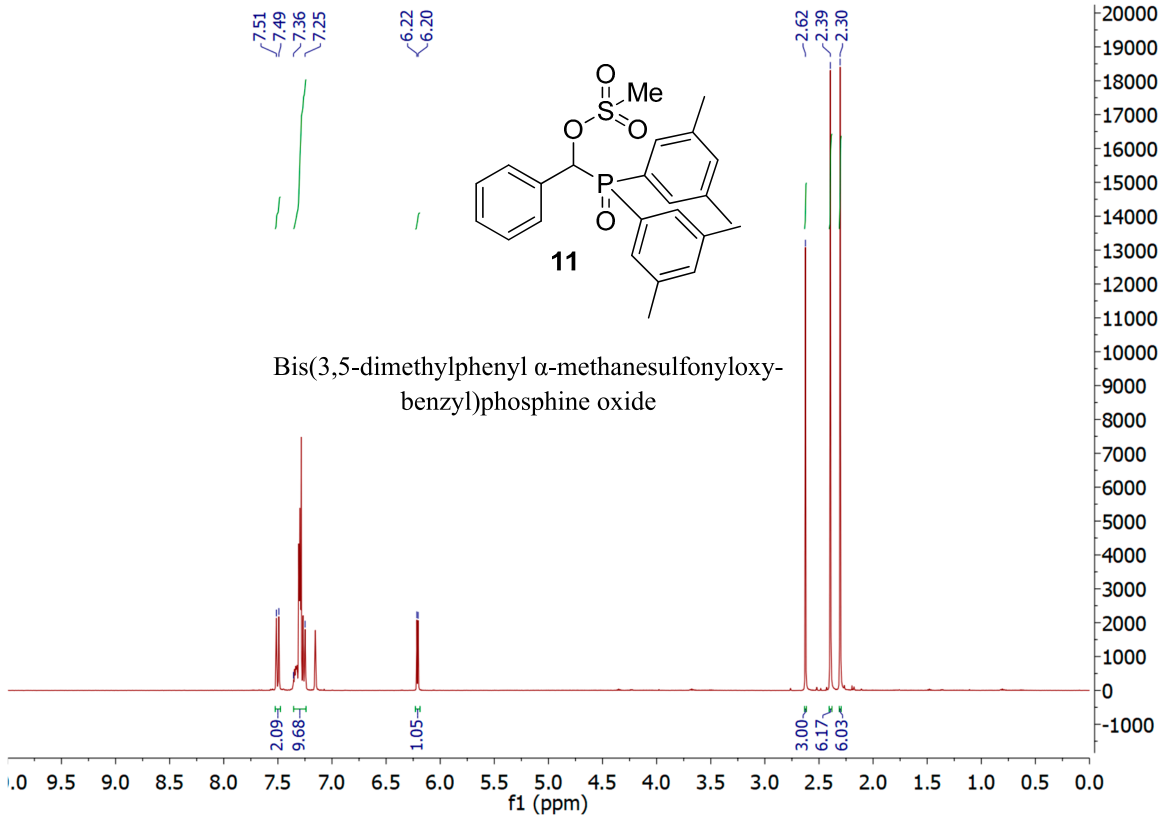

$^{31}P\ \{^1H\}$ NMR (202 MHz, $CDCl_3$) spectra for 14a

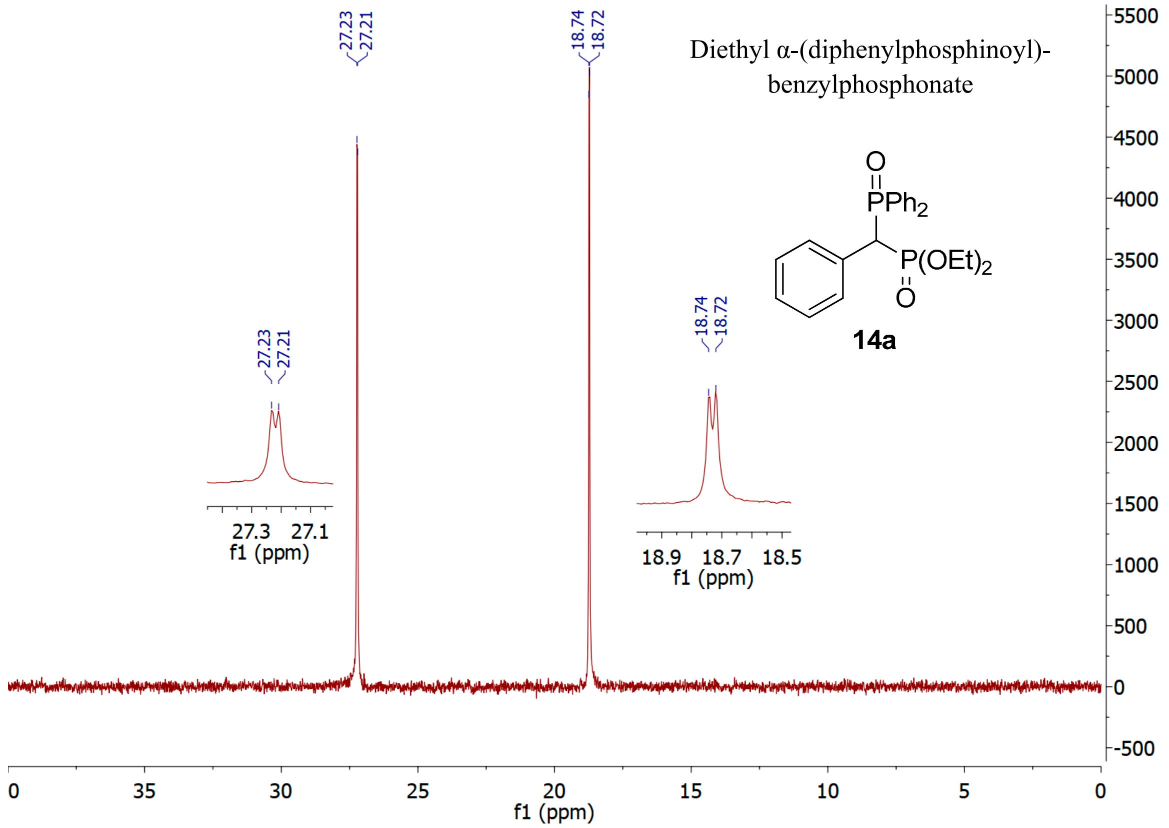


$^{13}C\ \{^1H\}$ NMR (126 MHz, $CDCl_3$) spectra for 14a

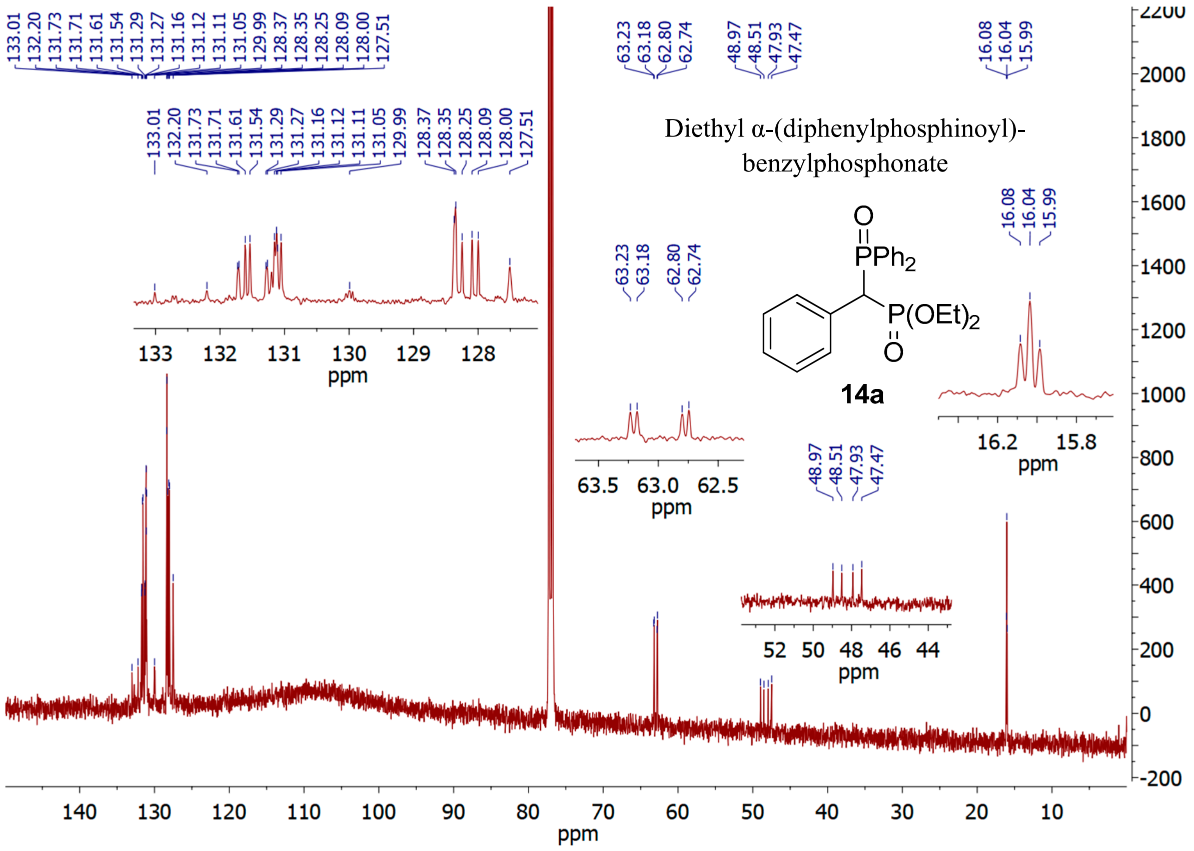

## $^{1}H$ NMR (500 MHz, $CDCl_3$) spectra for 14a

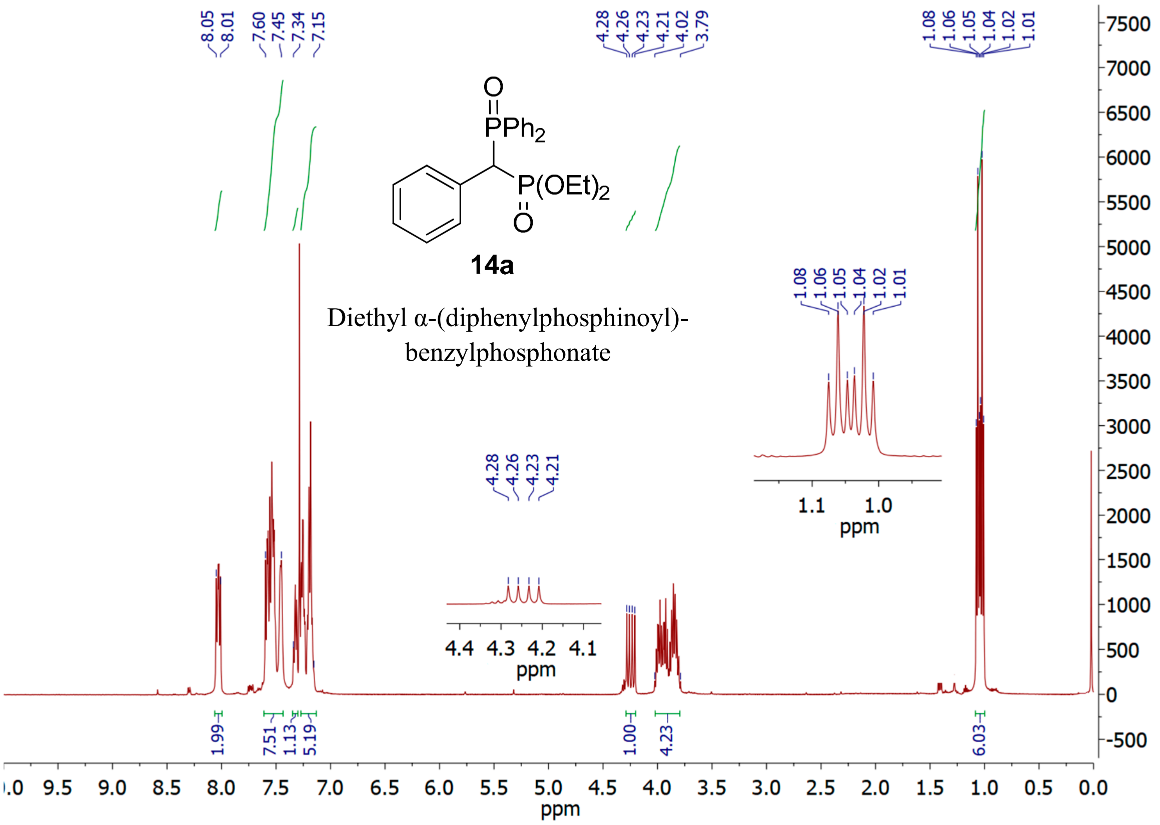


## $^{31}P$ {$^{1}H$} NMR (202 MHz, $CDCl_3$) spectra for 14b

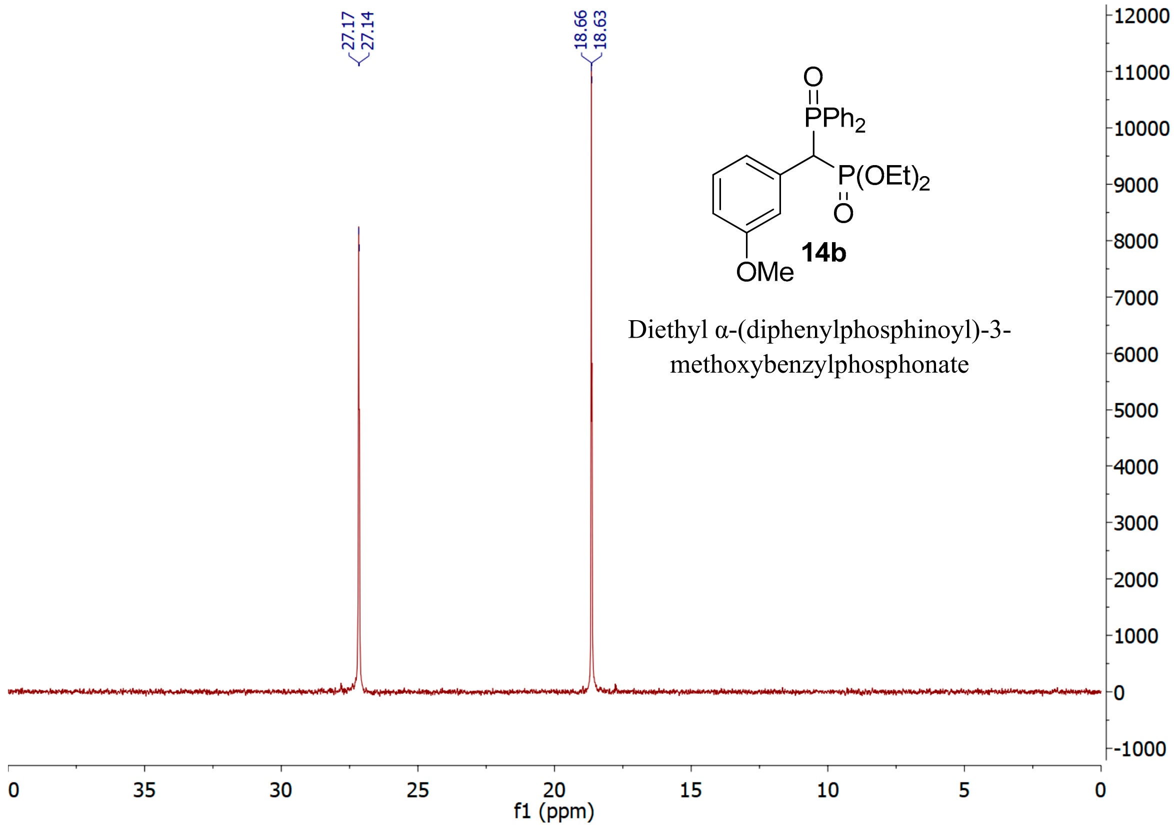

## $^{13}$C {$^{1}$H} NMR (126 MHz, $CDCl_3$) spectra for 14b

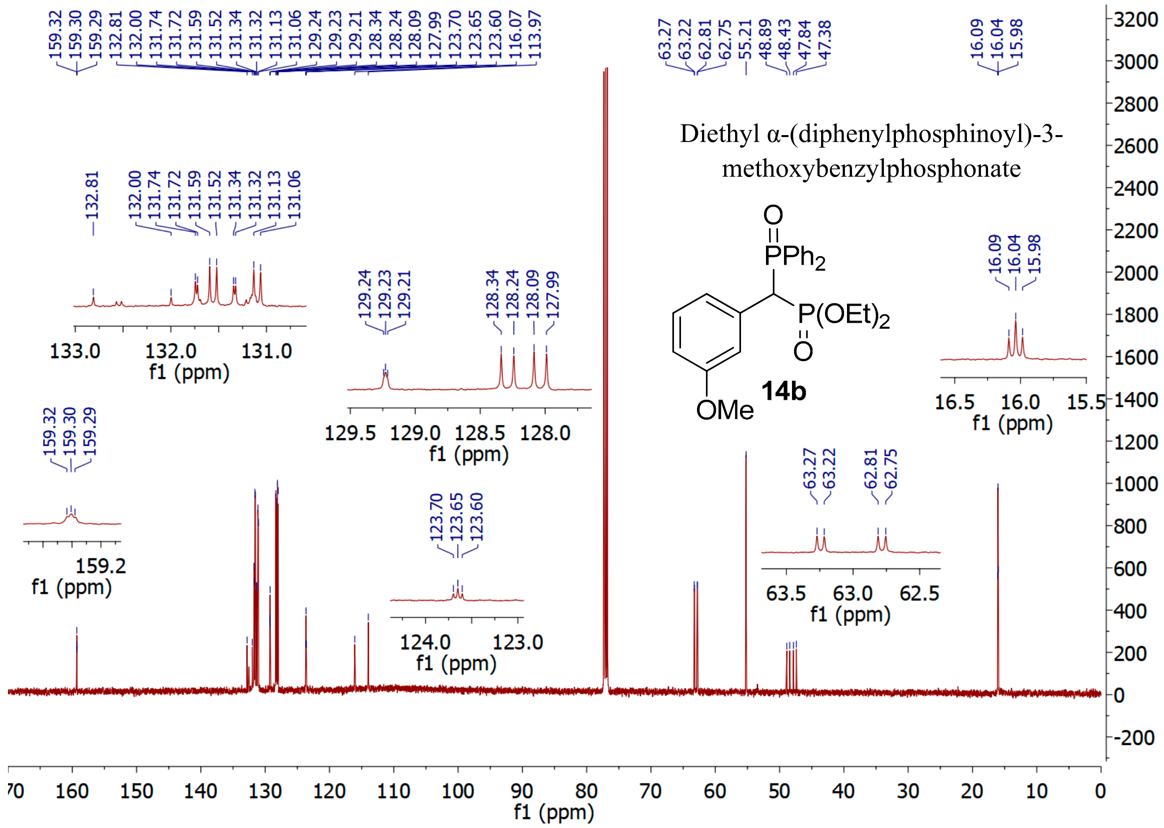


## $^{1}$H NMR (500 MHz, $CDCl_3$) spectra for 14b

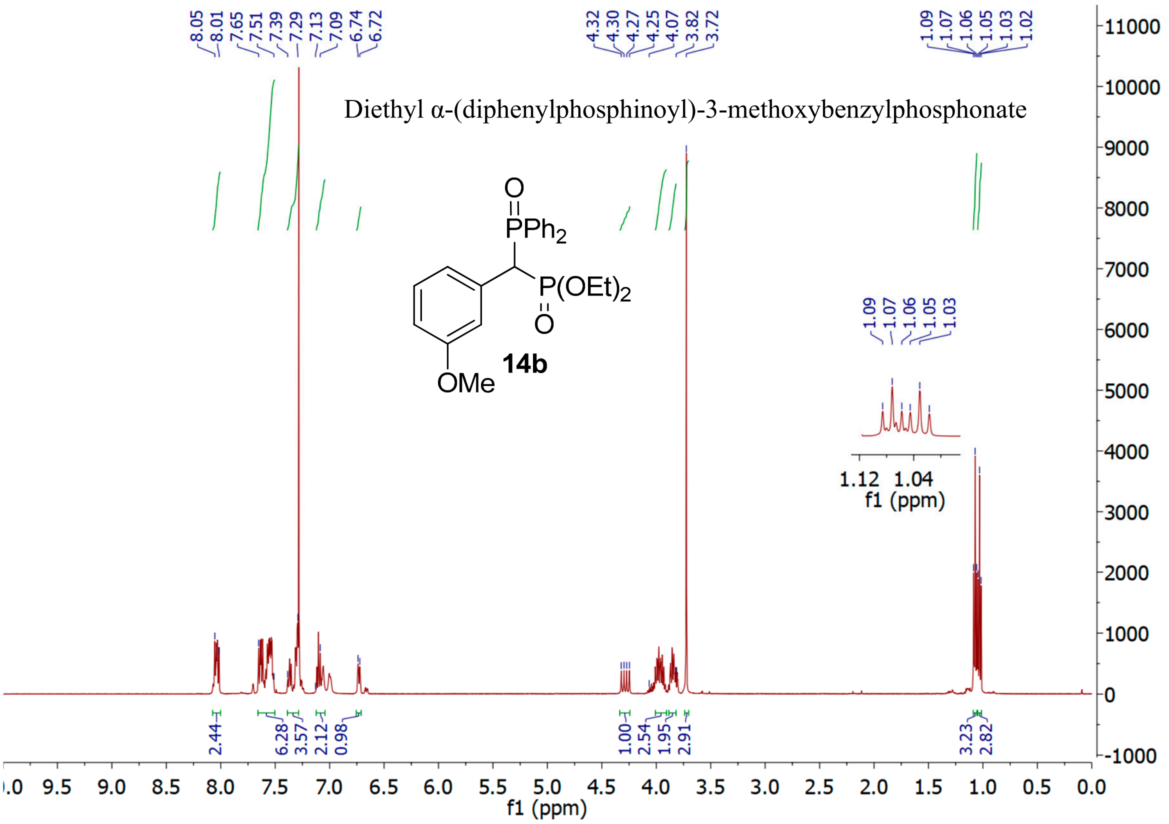

$^{31}P\ \{^1H\}$ NMR (202 MHz, $CDCl_3$) spectra for 14c

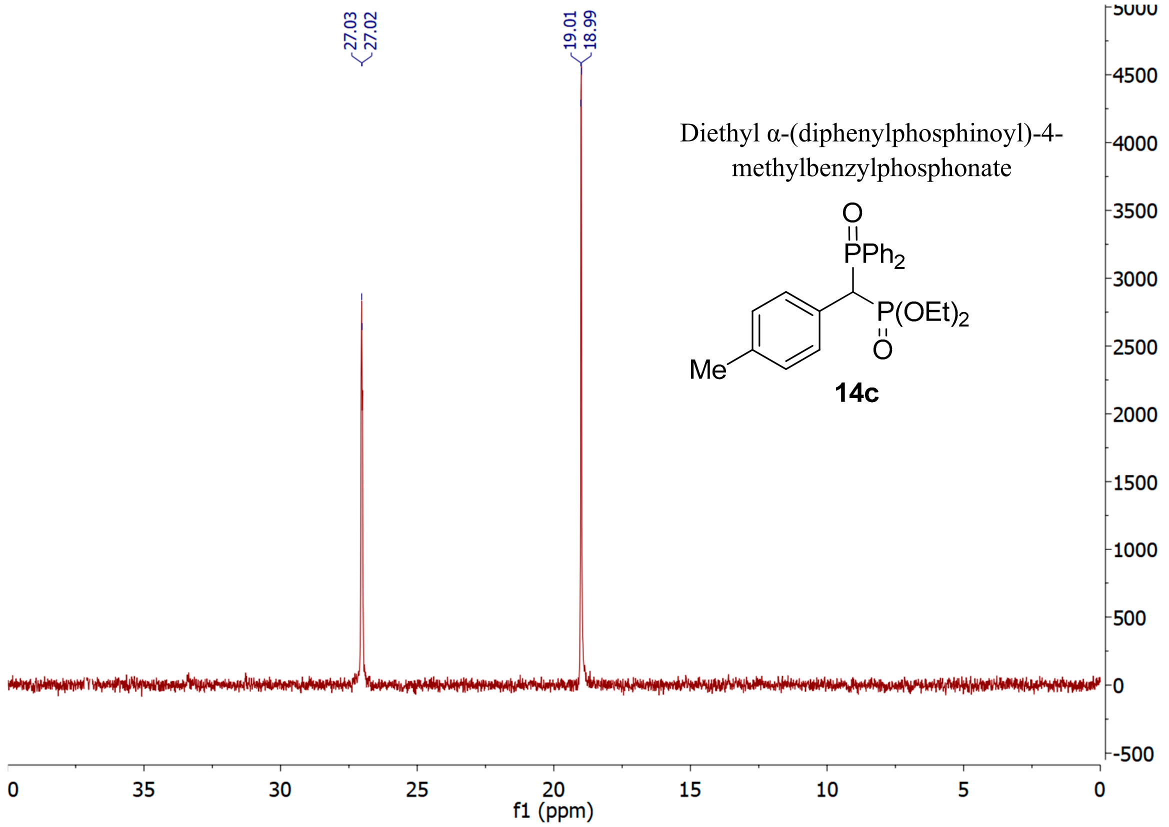


$^{13}C\ \{^1H\}$ NMR (126 MHz, $CDCl_3$) spectra for 14c

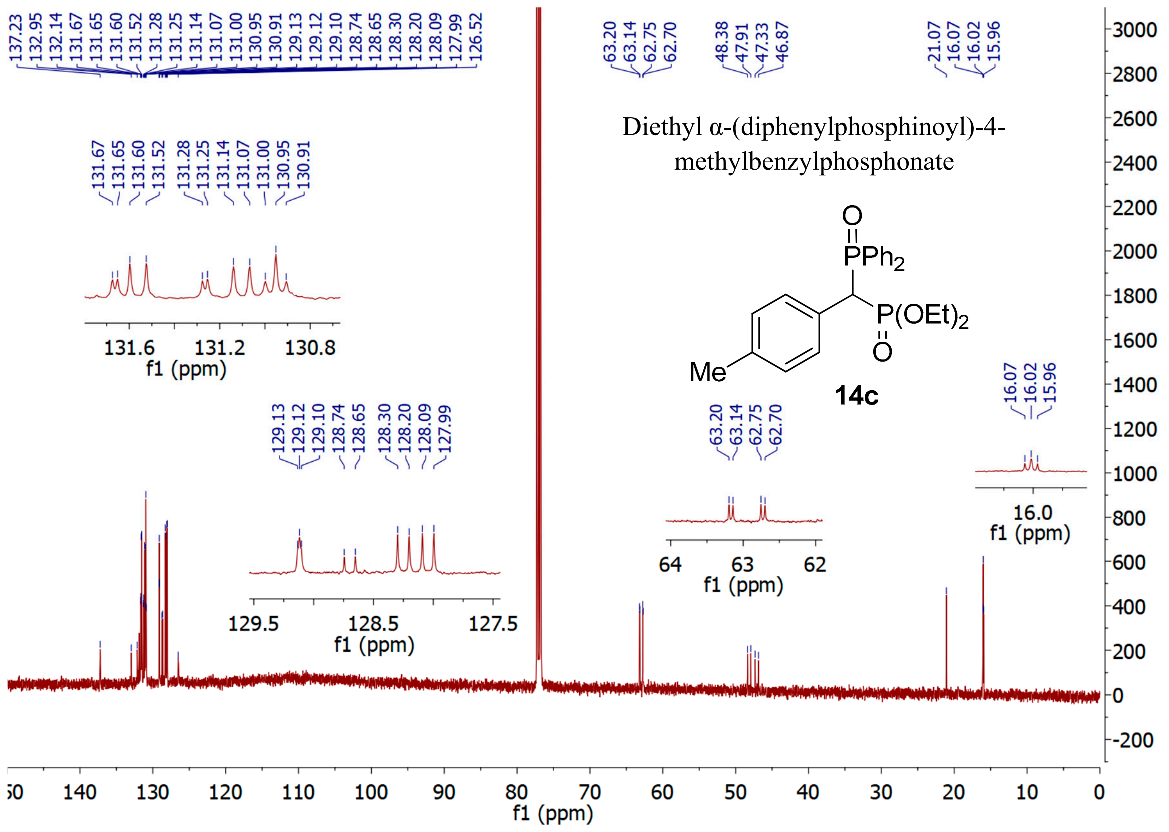

$^{1}H$ NMR (500 MHz, $CDCl_3$) spectra for 14c

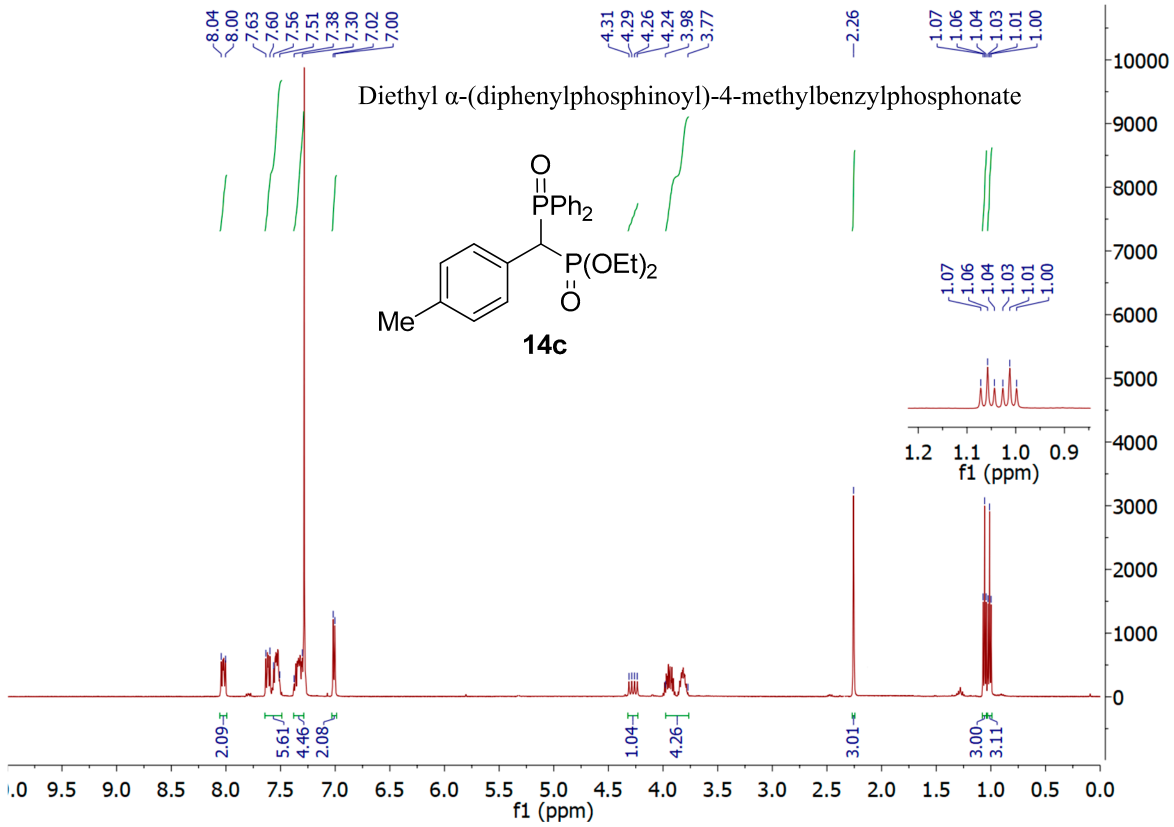


$^{31}P$ {$^{1}H$} NMR (202 MHz, $CDCl_3$) spectra for 14d

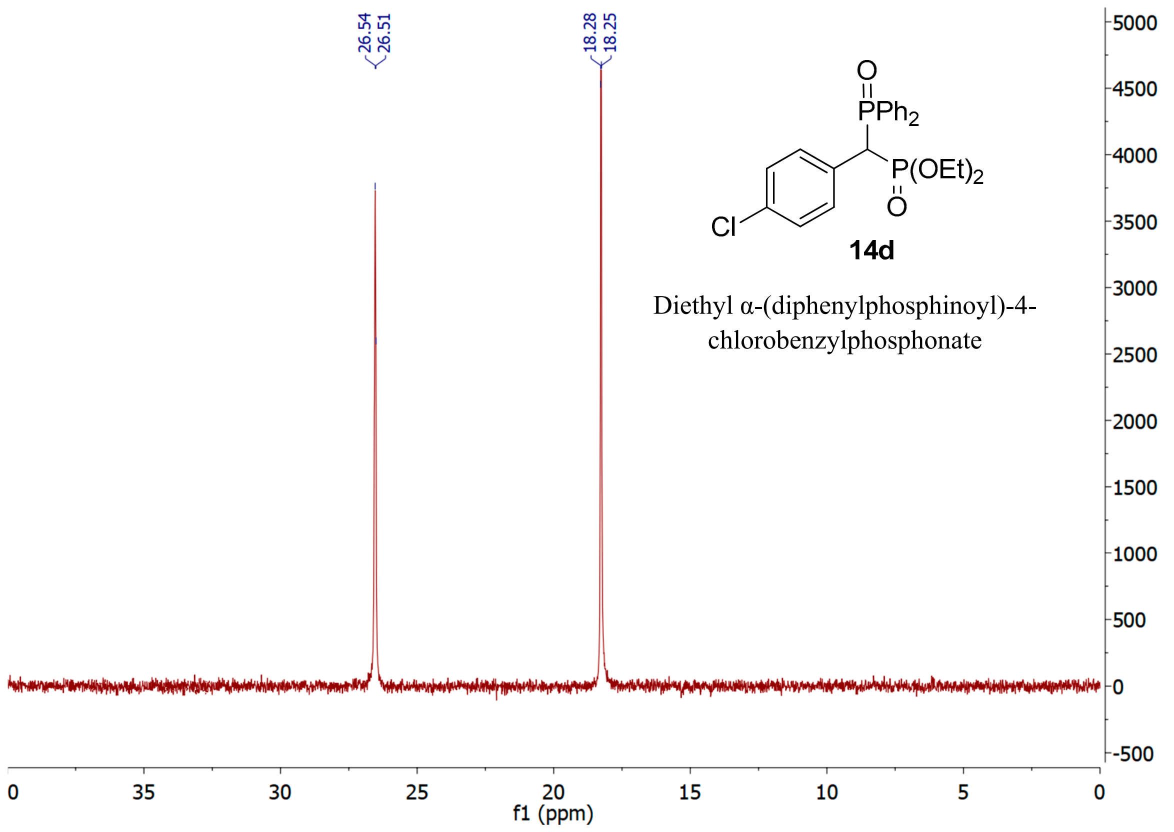

**$^{13}C$ {$^{1}H$} NMR (126 MHz, $CDCl_3$) spectra for 14d**

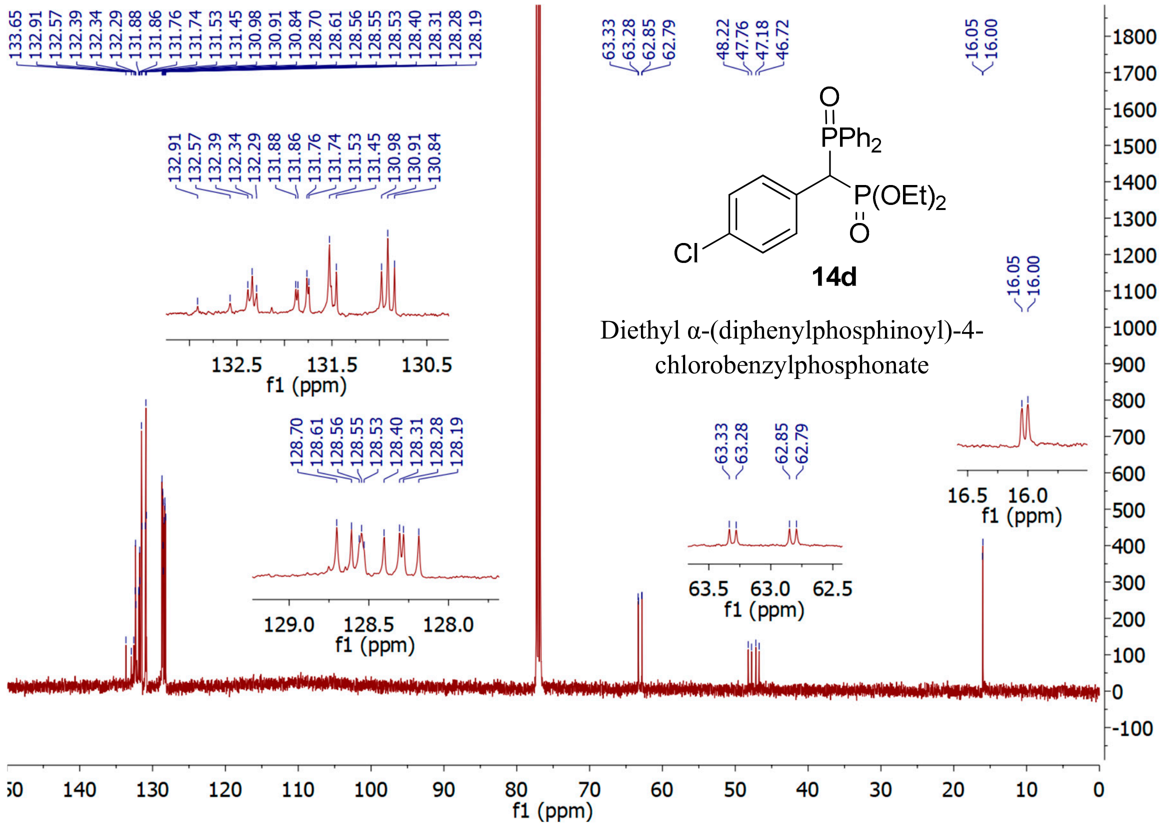


**$^{1}H$ NMR (500 MHz, $CDCl_3$) spectra for 14d**

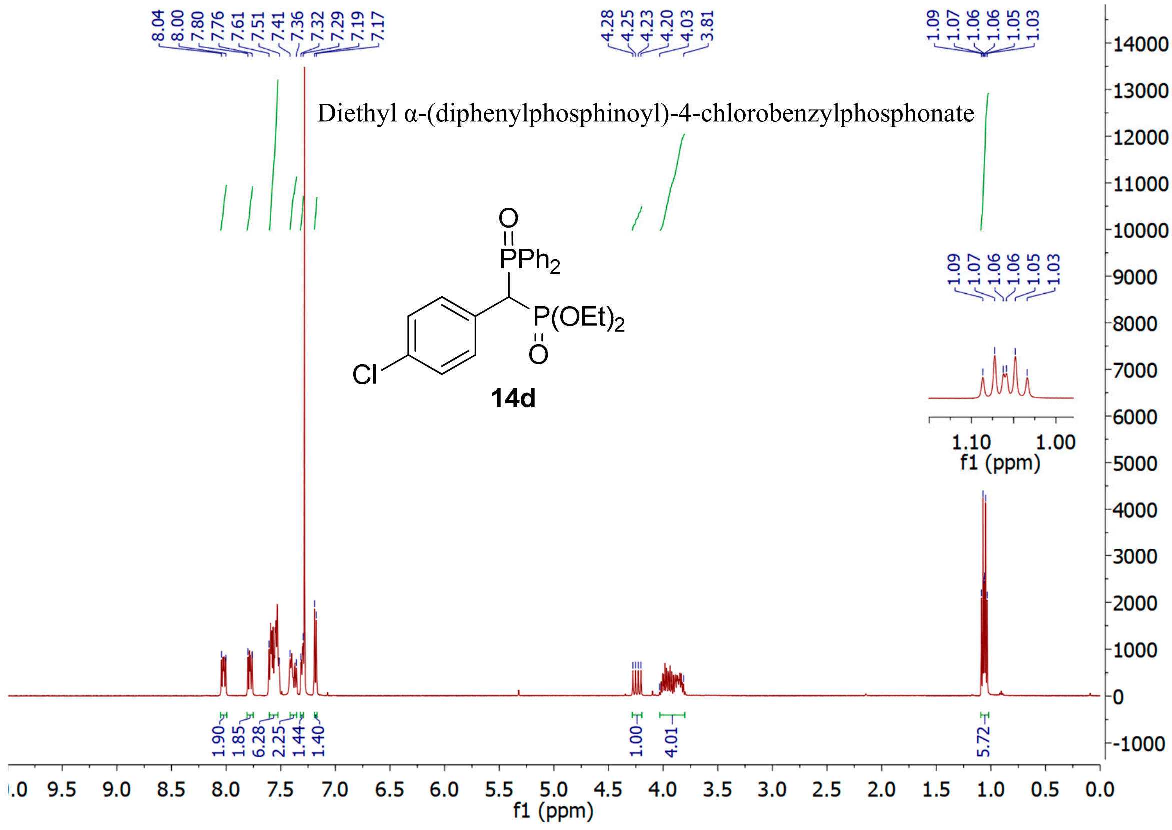

### $^{31}P$ {$^{1}H$} NMR (202 MHz, $CDCl_3$) spectra for 15

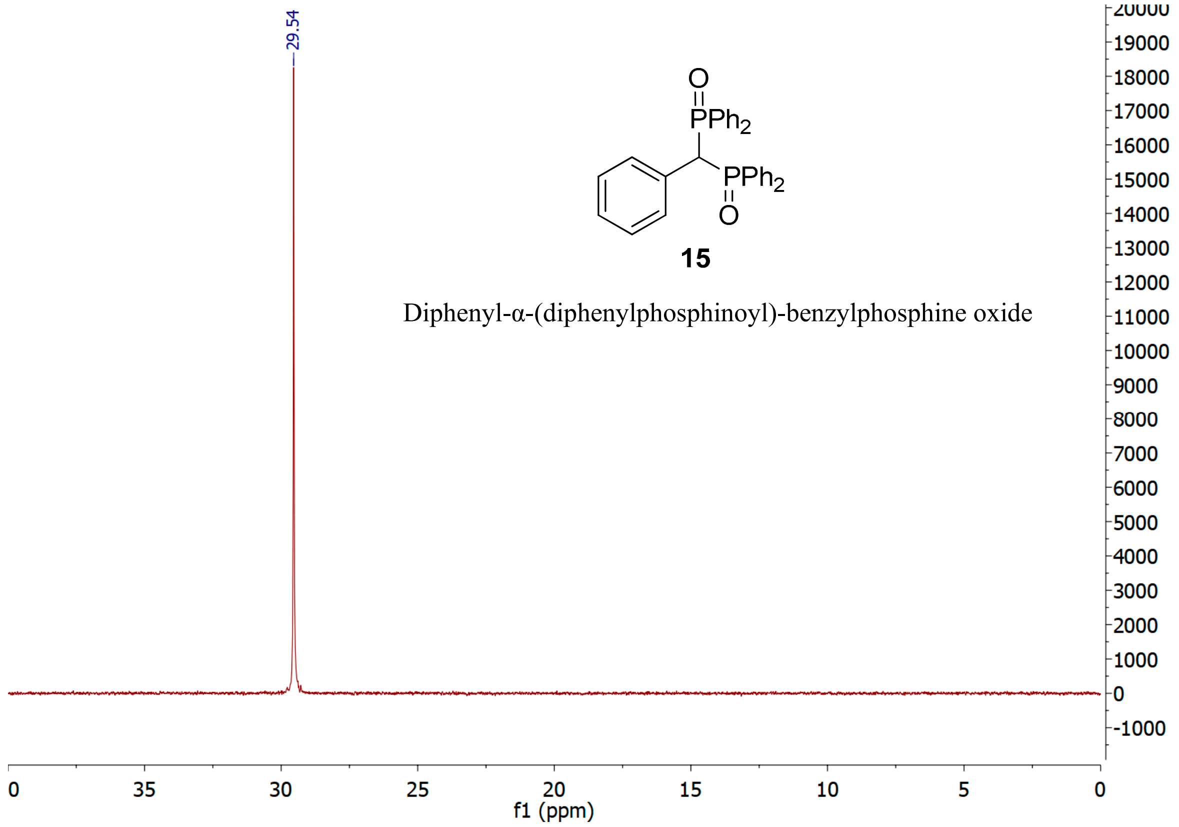


### $^{13}C$ {$^{1}H$} NMR (126 MHz, $CDCl_3$) spectra for 15

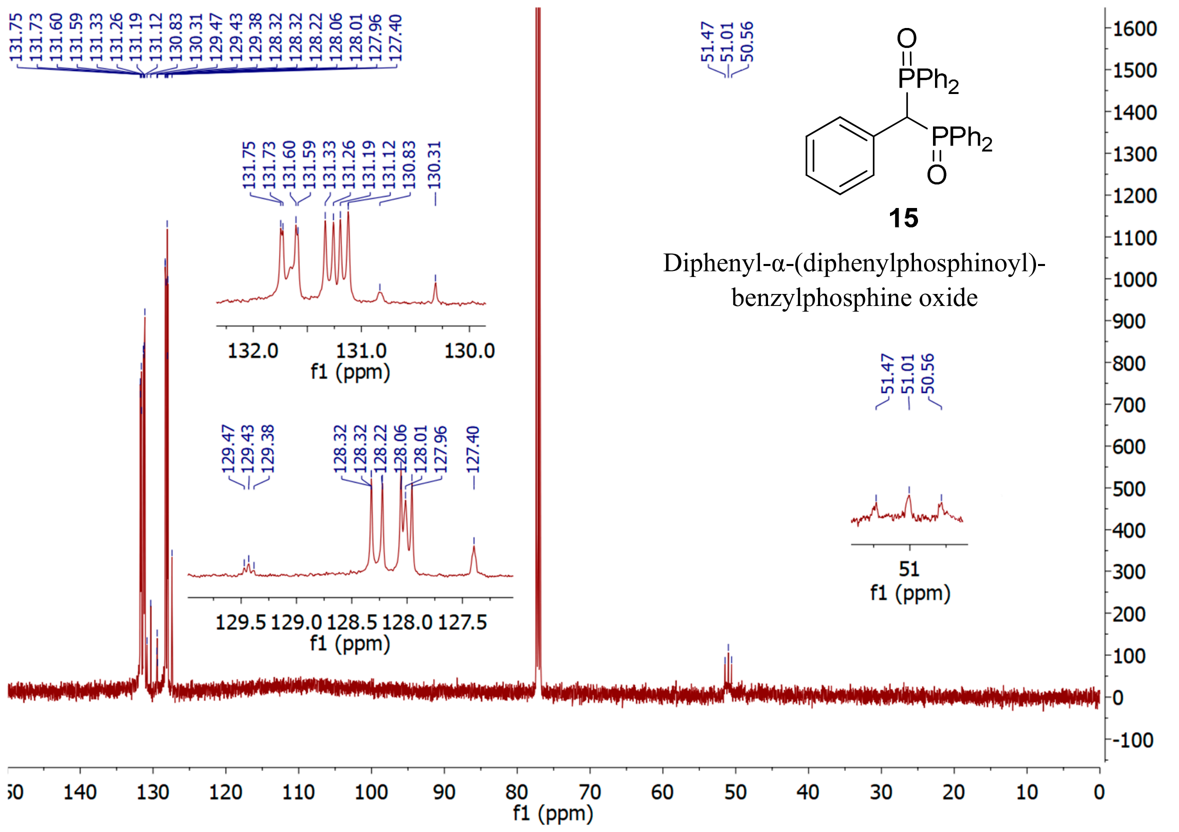

## $^{1}$H NMR (500 MHz, $CDCl_3$) spectra for 15

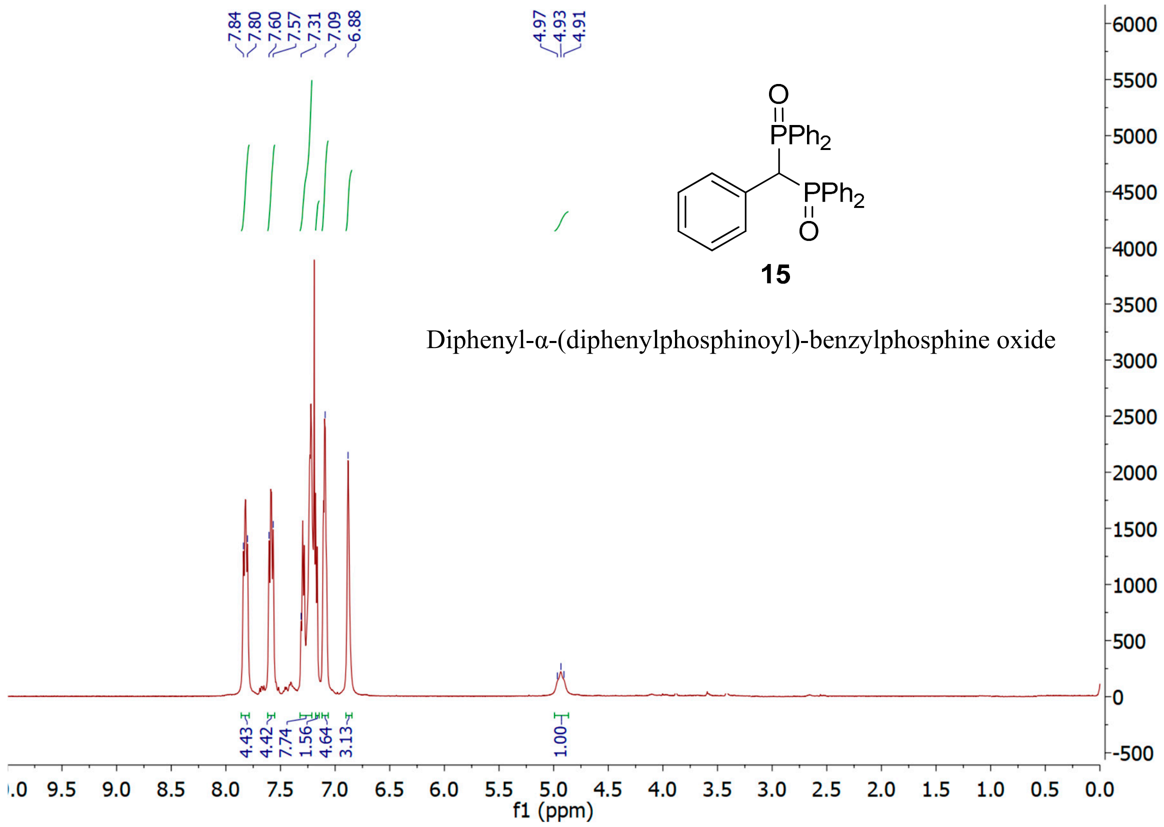


## $^{31}$P {$^{1}$H} NMR (202 MHz, $CDCl_3$) spectra for 16

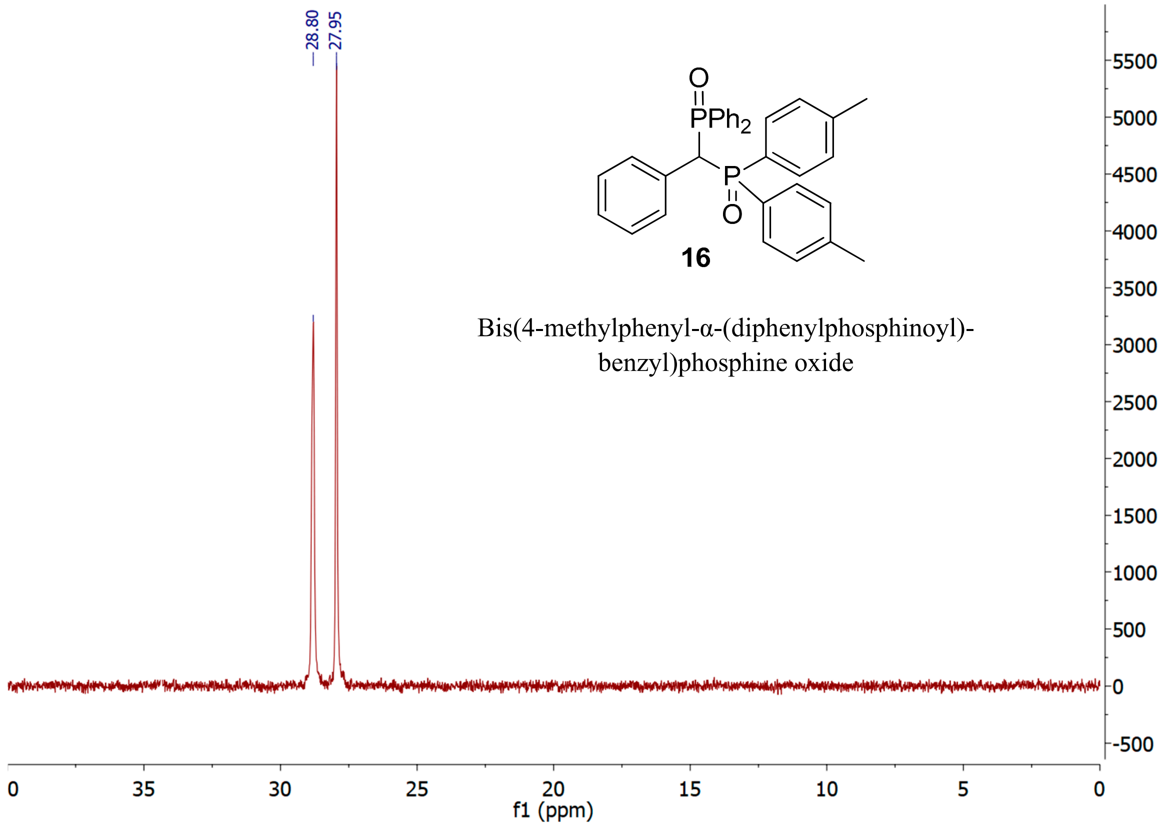

## $^{13}C\ \{^1H\}$ NMR (126 MHz, $CDCl_3$) spectra for 16

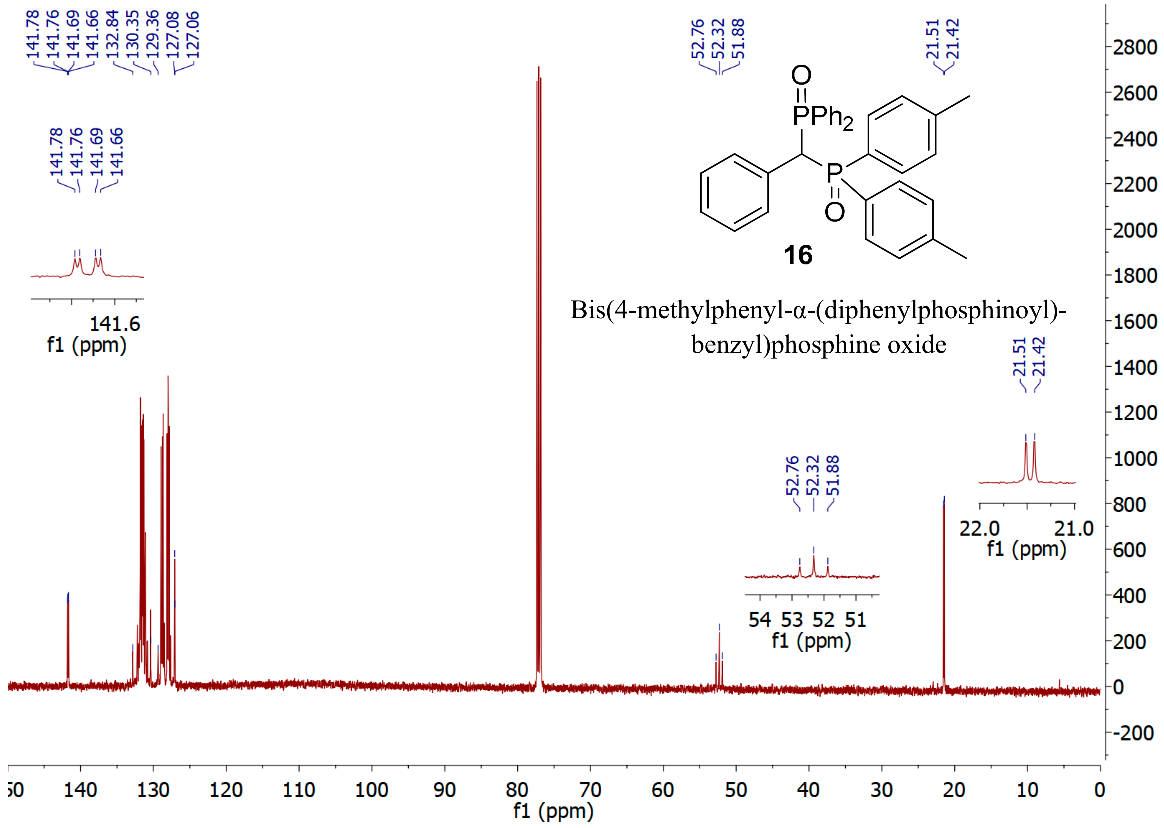


## $^1H$ NMR (500 MHz, $CDCl_3$) spectra for 16

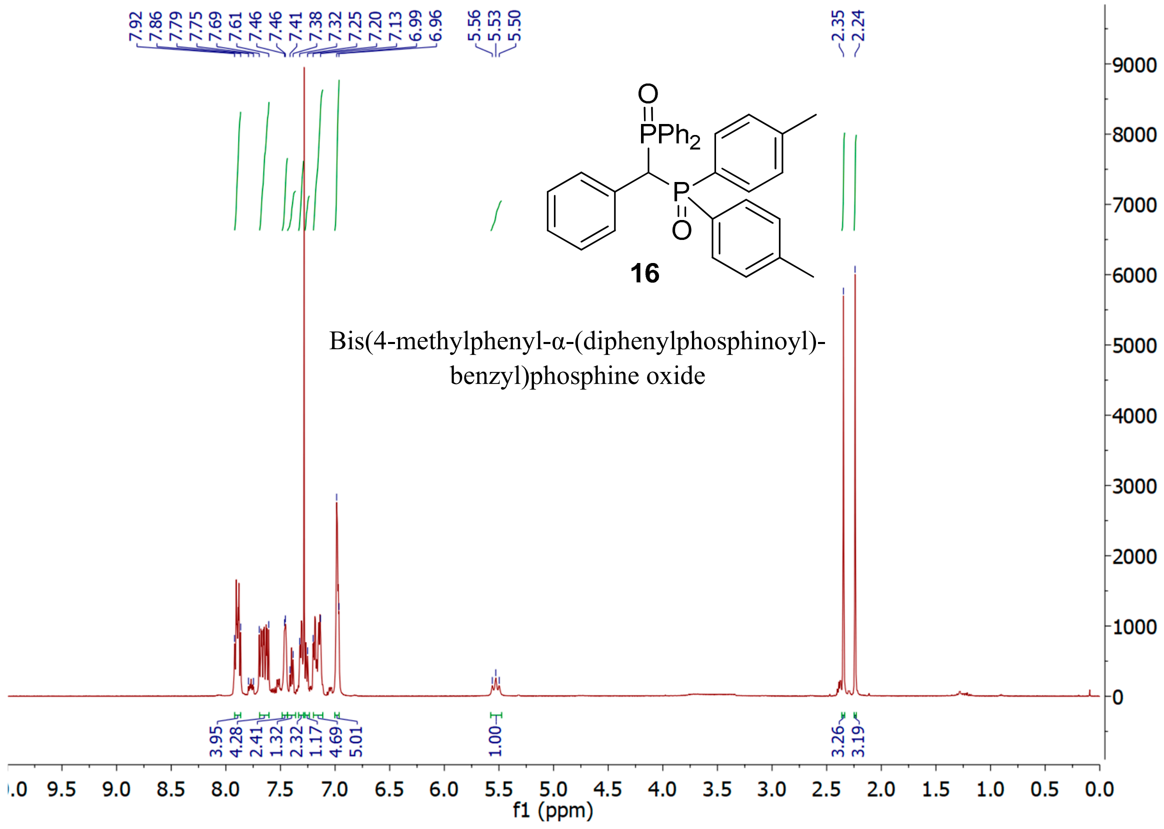

**$^{31}P$ {$^{1}H$} NMR (202 MHz, $CDCl_3$) spectra for 17**

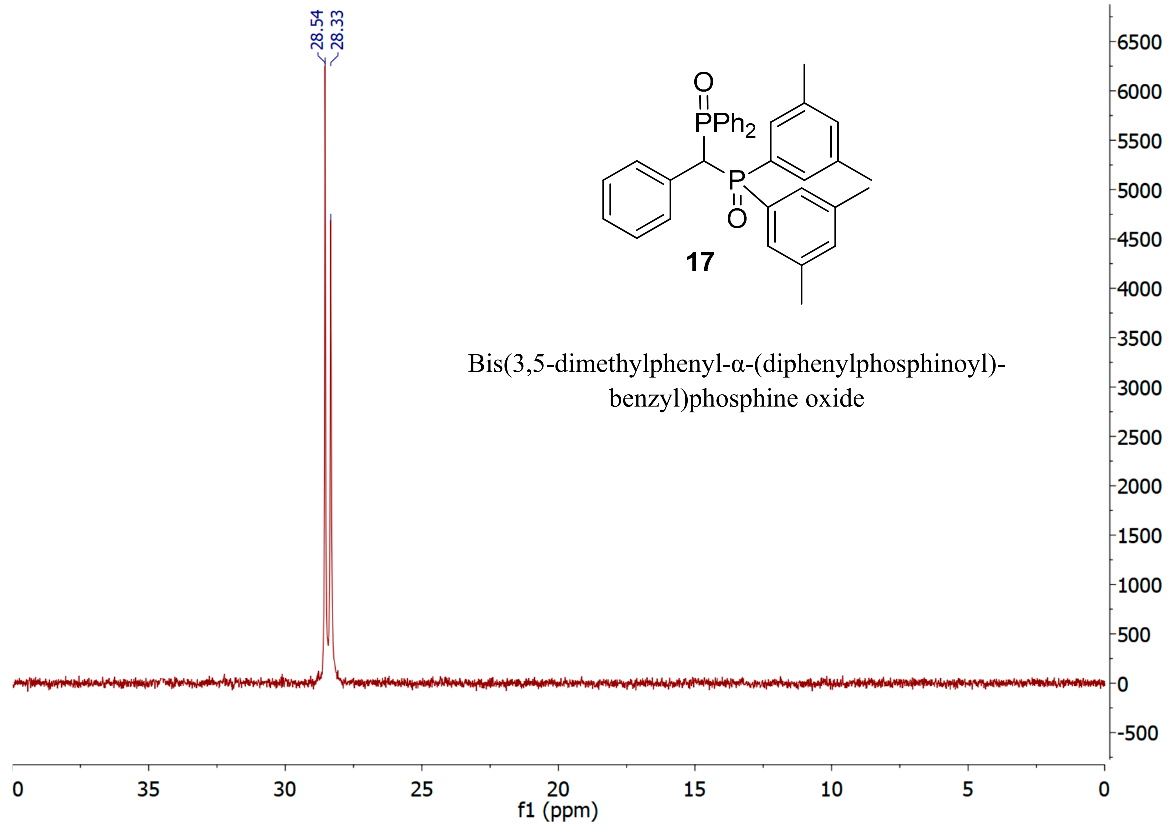


**$^{13}C$ {$^{1}H$} NMR (126 MHz, $CDCl_3$) spectra for 17**

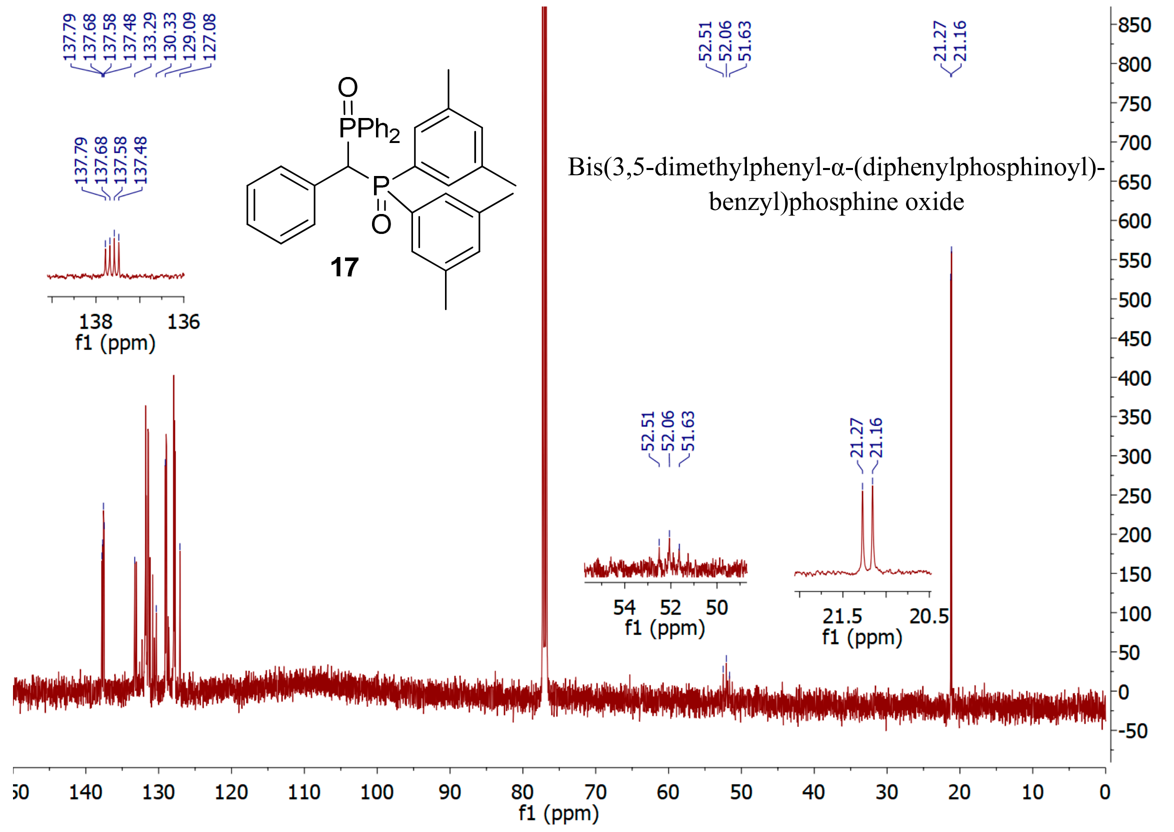

**$^1$H NMR (500 MHz, $CDCl_3$) spectra for 17**

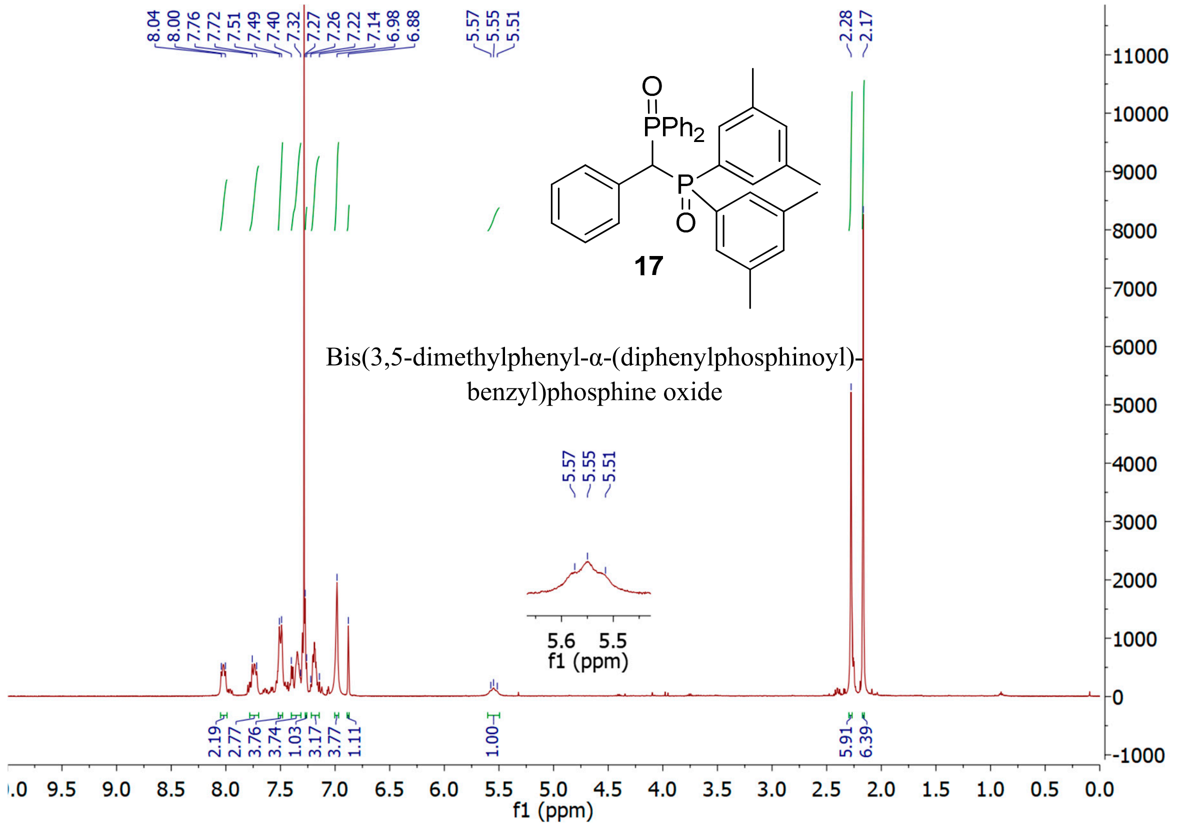


**$^{31}$P {$^1$H} NMR (202 MHz, $CDCl_3$) spectra for 18a**

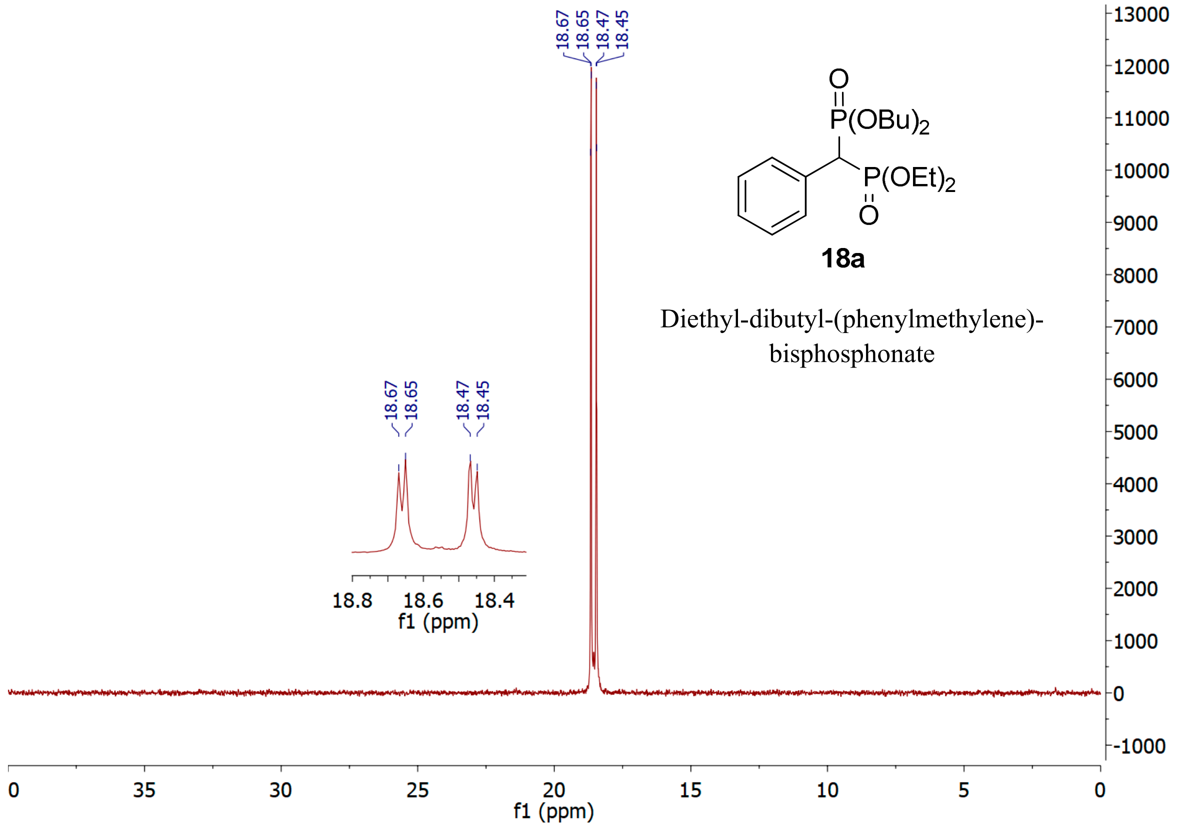

## $^{13}C\ \{^{1}H\}$ NMR (126 MHz, $CDCl_3$) spectra for 18a

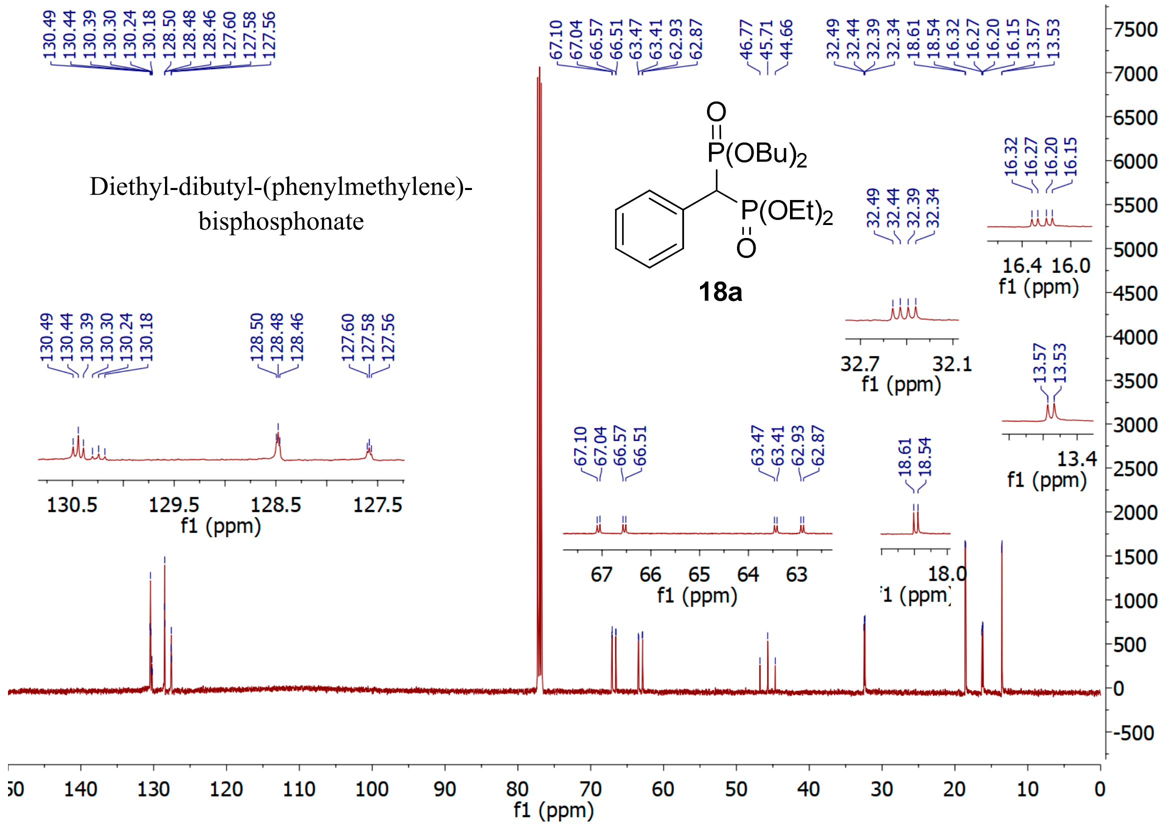


## $^{1}H$ NMR (500 MHz, $CDCl_3$) spectra for 18a

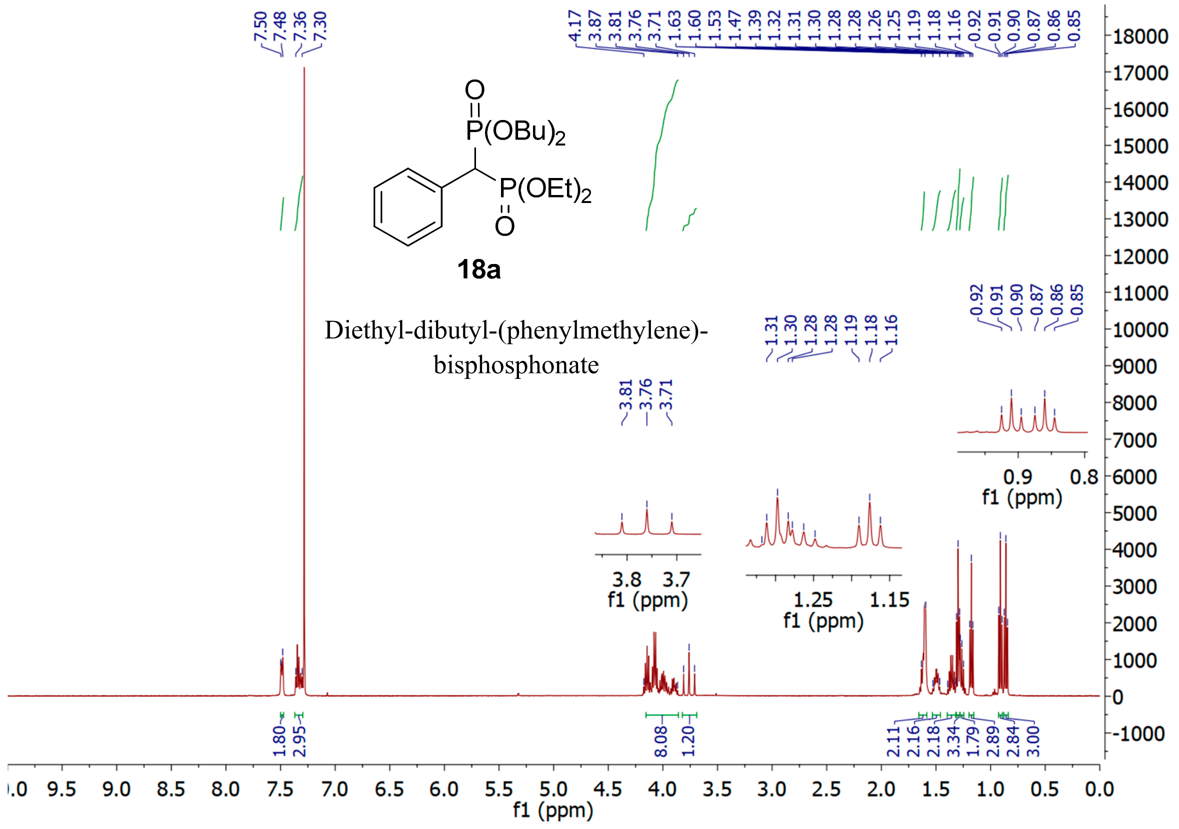

### $^{31}P$ {$^{1}H$} NMR (202 MHz, $CDCl_3$) spectra for 18c

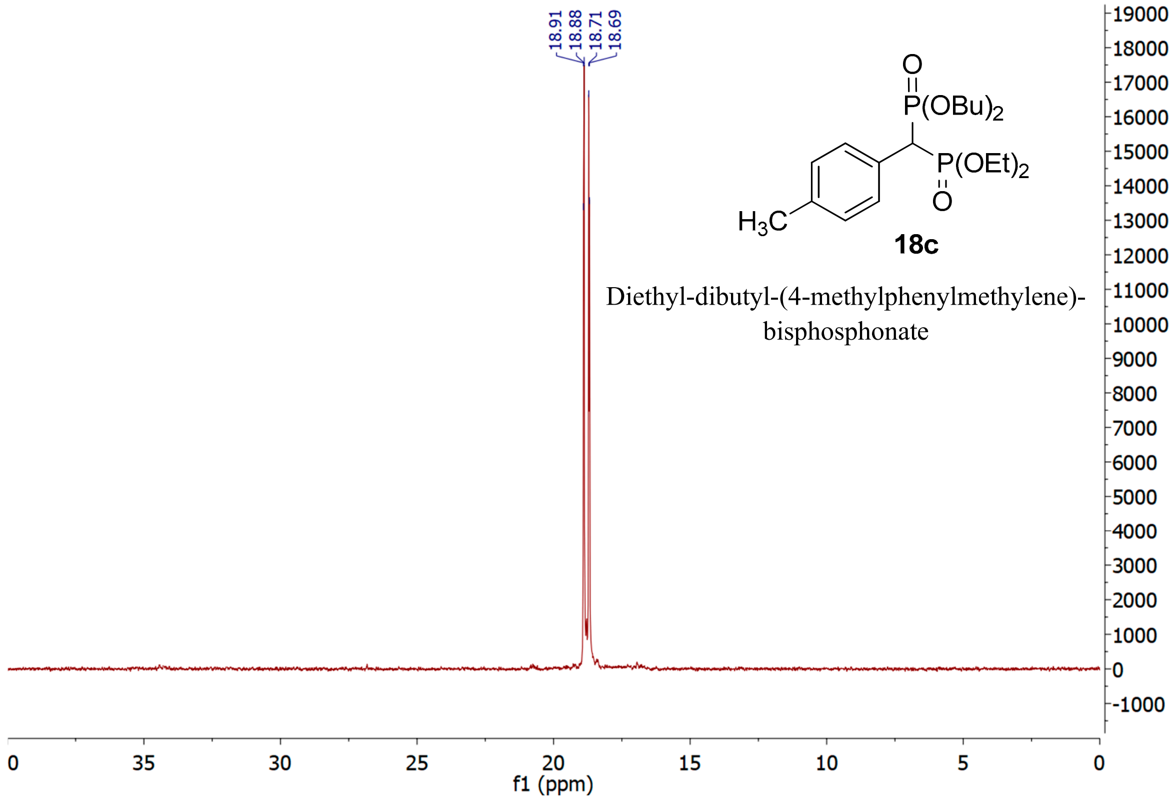


### $^{13}C$ {$^{1}H$} NMR (126 MHz, $CDCl_3$) spectra for 18c

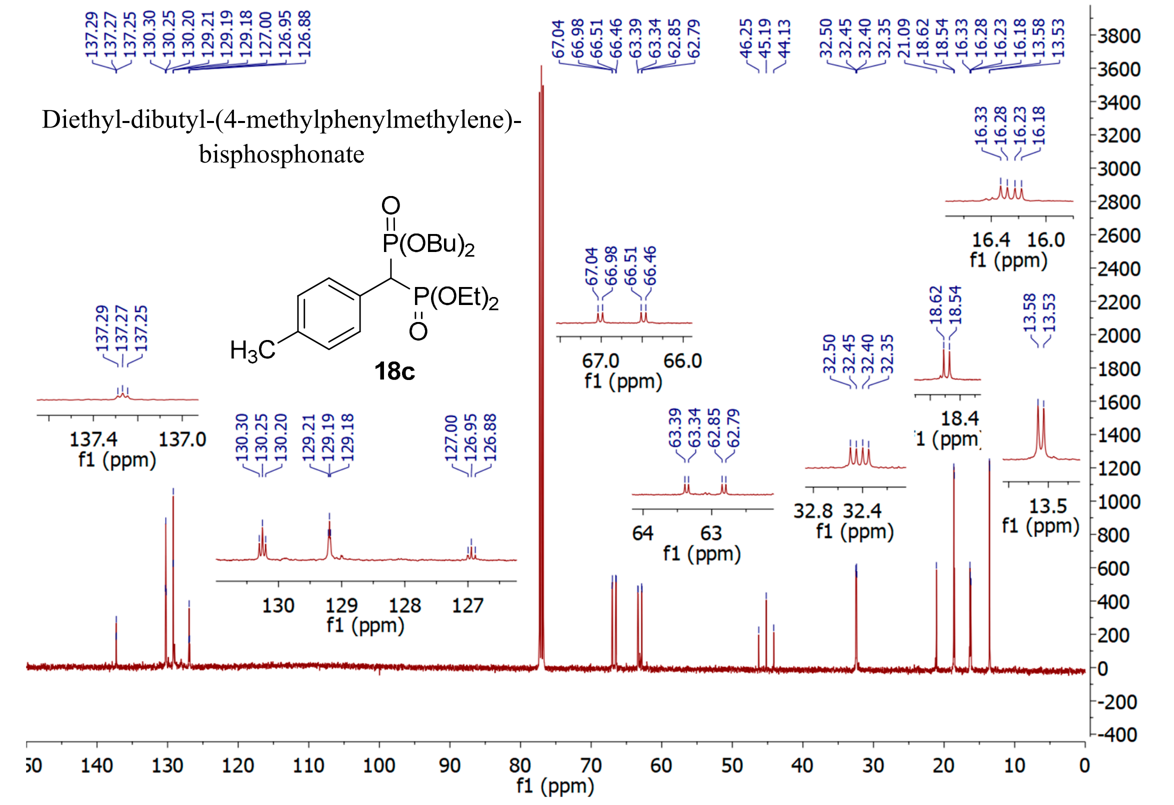

## $^{1}H$ NMR (500 MHz, $CDCl_3$) spectra for 18c

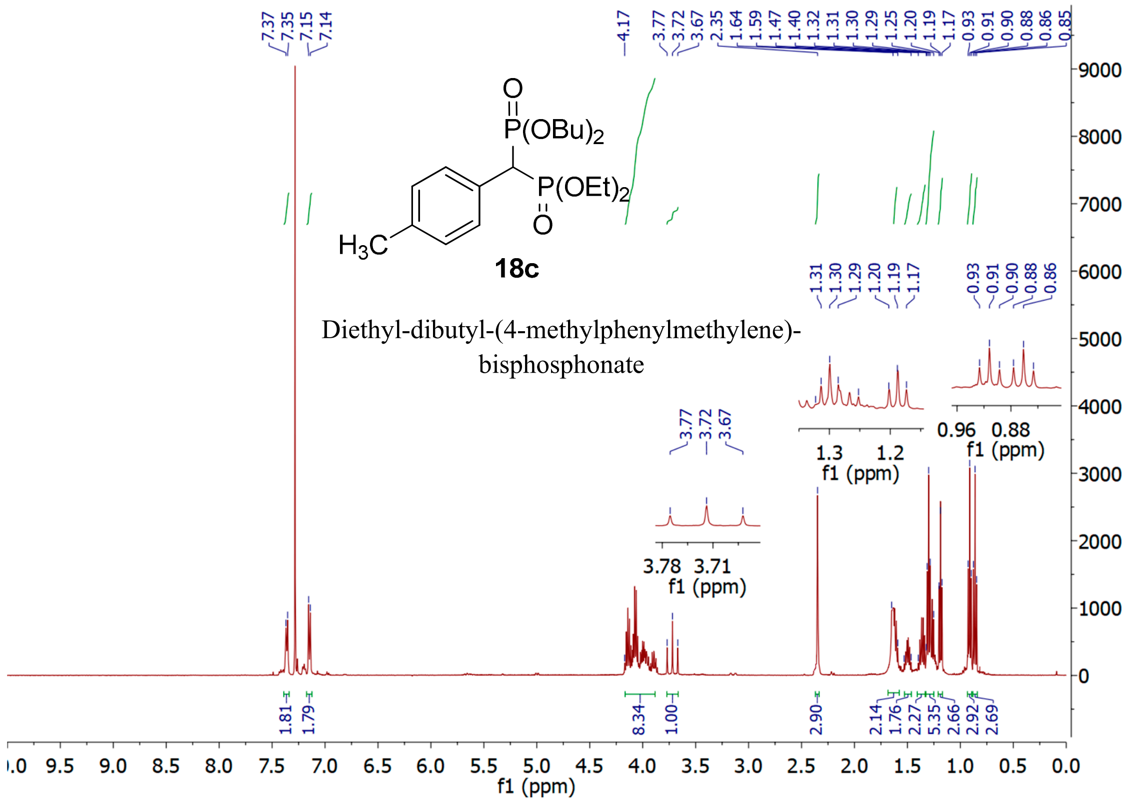


## $^{31}P$ {$^{1}H$} NMR (202 MHz, $CDCl_3$) spectra for 18e

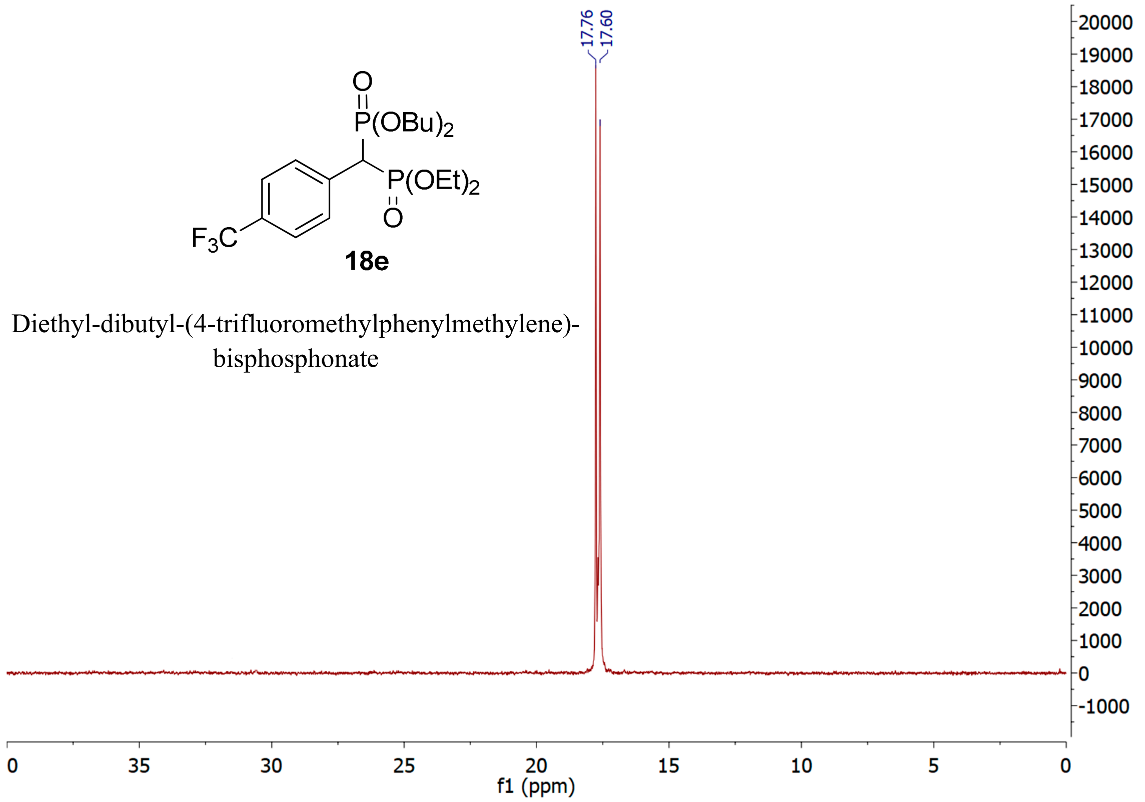

**$^{13}C\ \{^1H\}$ NMR (126 MHz, $CDCl_3$) spectra for 18e**

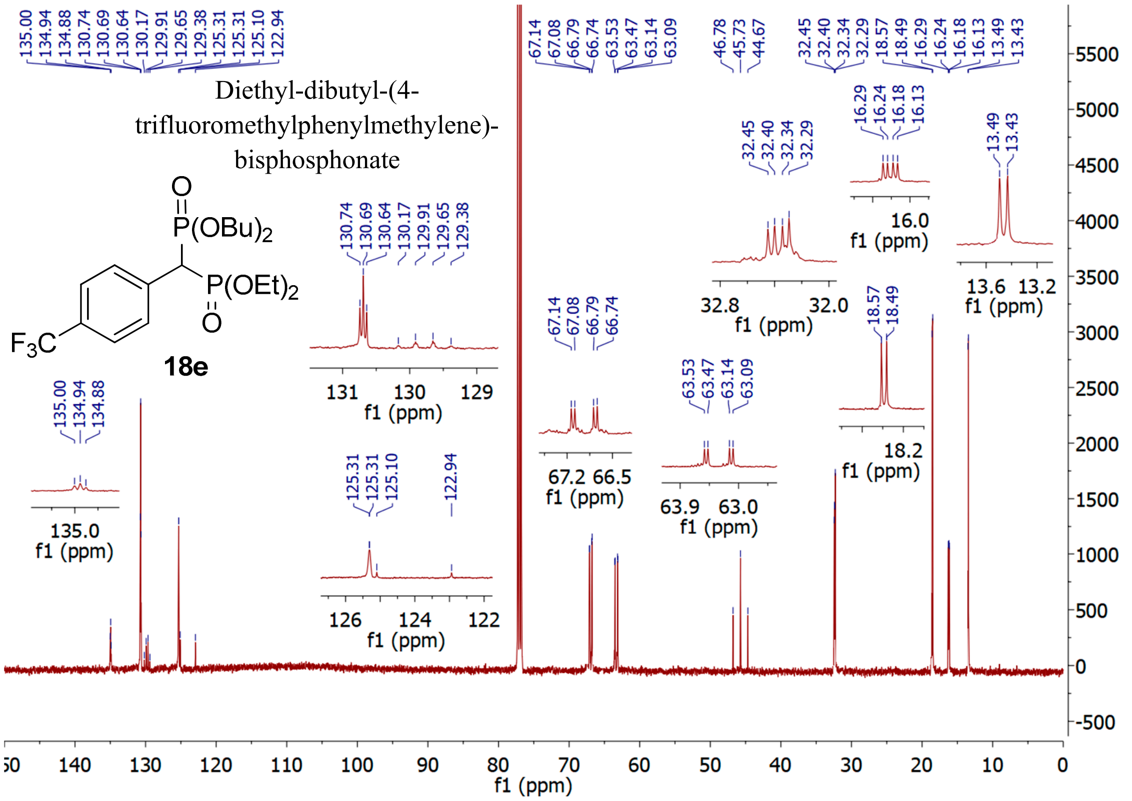


**$^1H$ NMR (500 MHz, $CDCl_3$) spectra for 18e**

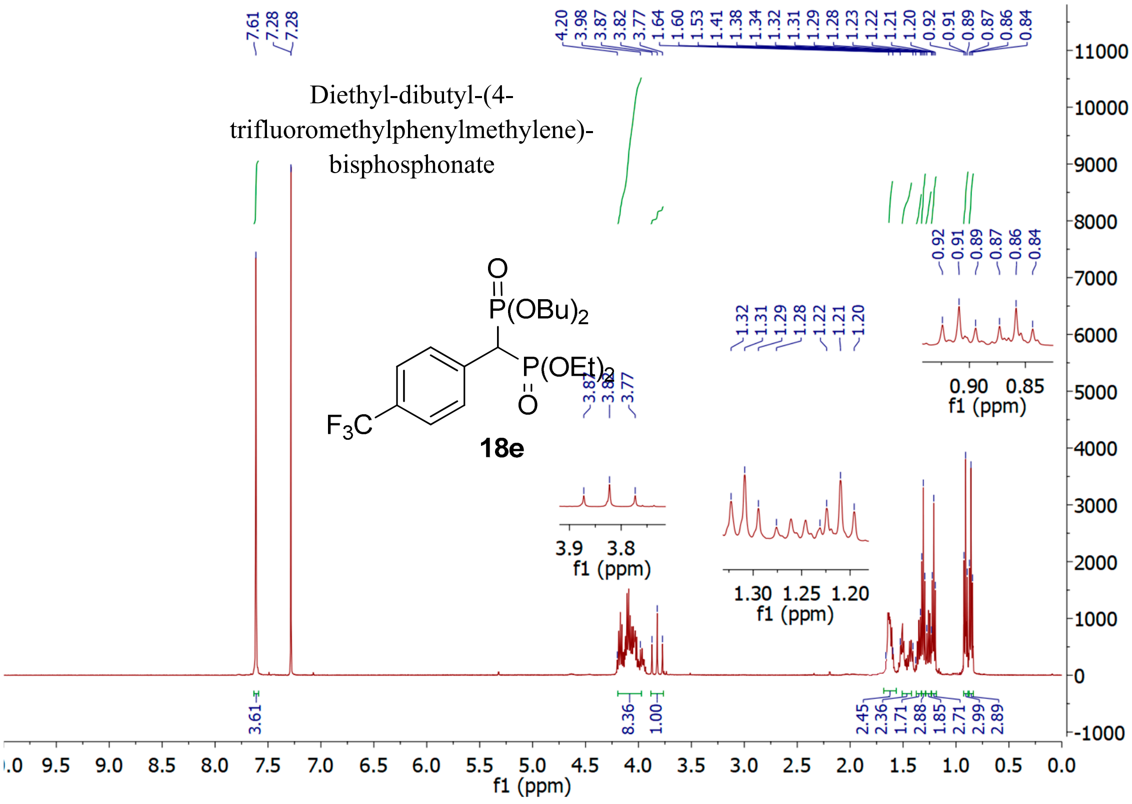

## $^{31}P\ \{^{1}H\}$ NMR (202 MHz, $CDCl_3$) spectra for 19

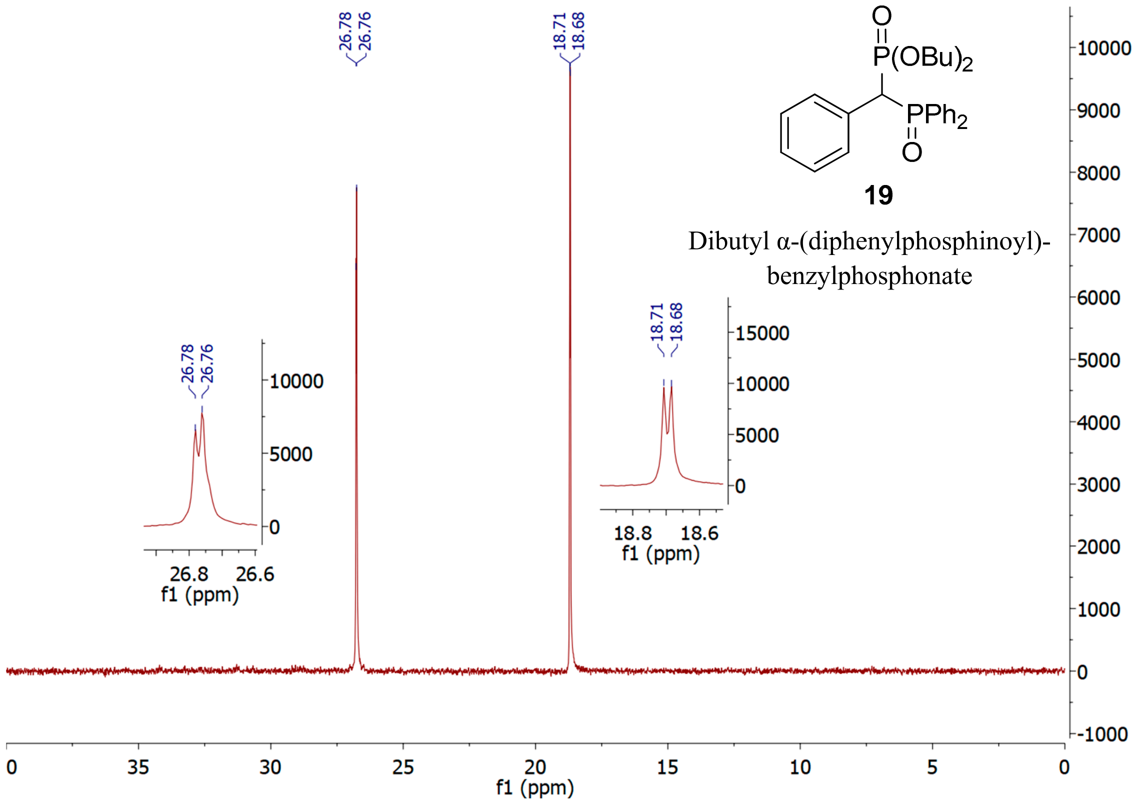


## $^{13}C\ \{^{1}H\}$ NMR (126 MHz, $CDCl_3$) spectra for 19

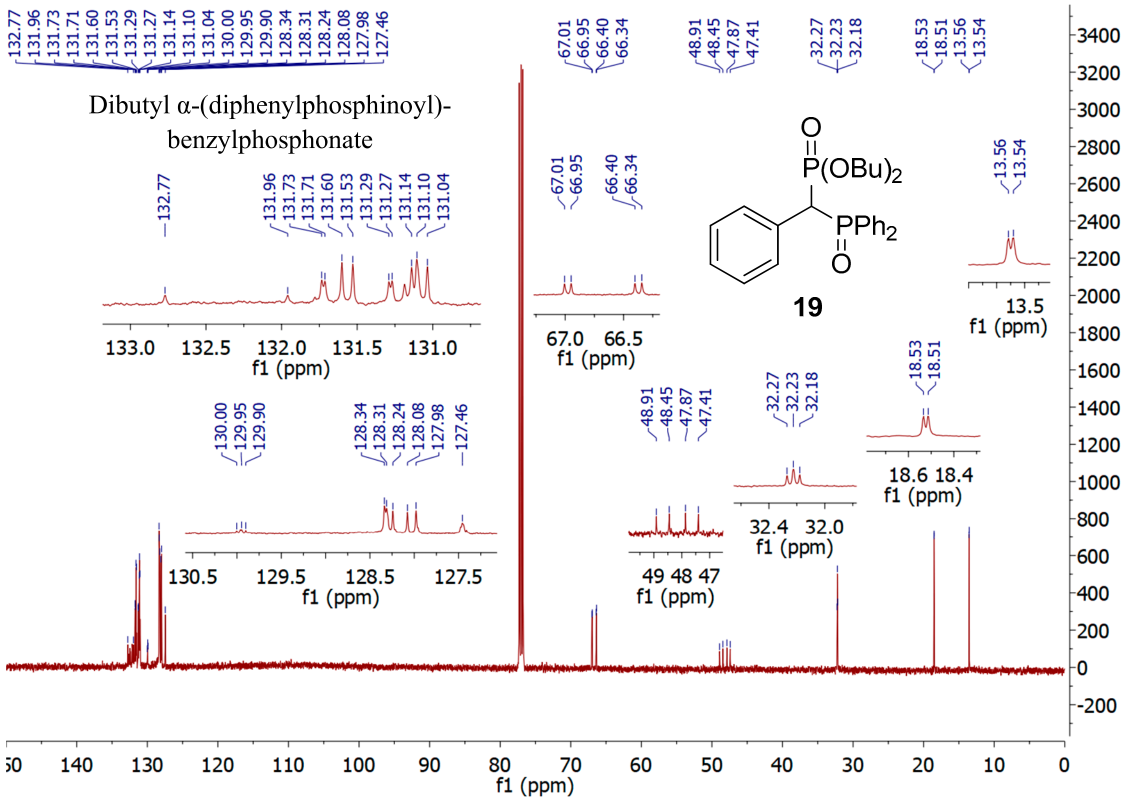

## $^{1}H$ NMR (500 MHz, $CDCl_3$) spectra for 19

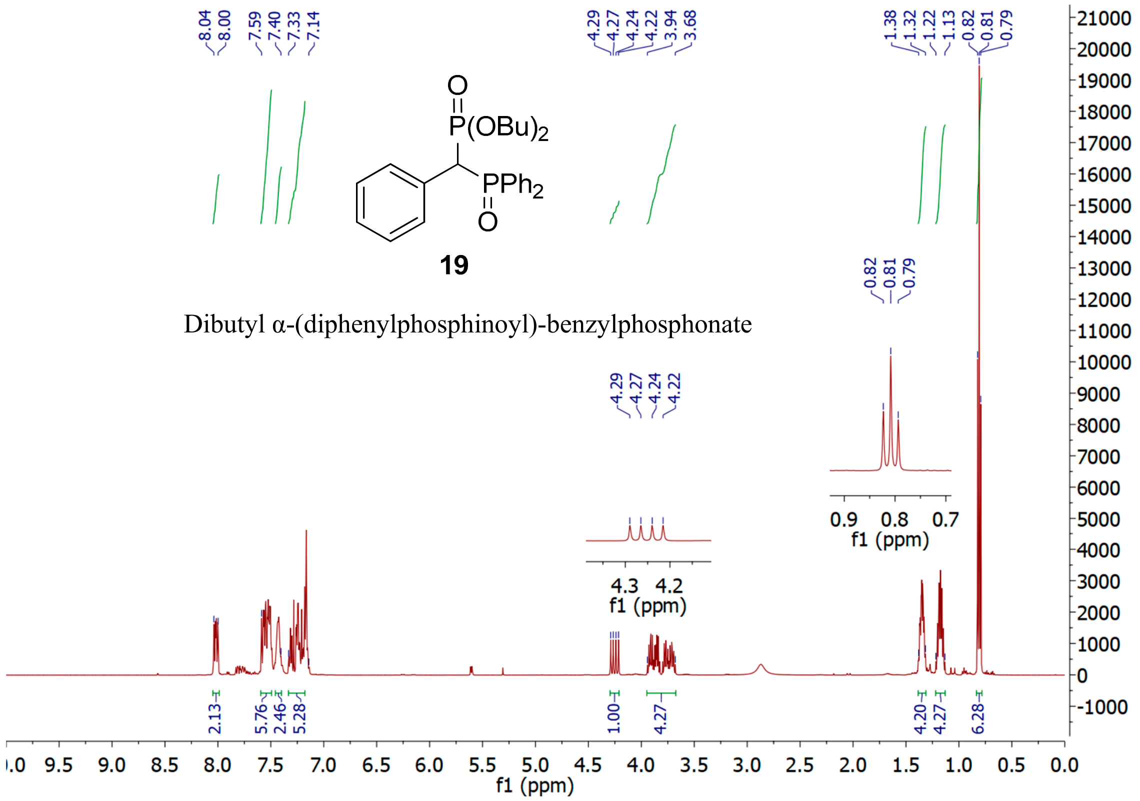


## $^{31}P$ {$^{1}H$} NMR (202 MHz, $CDCl_3$) spectra for 23

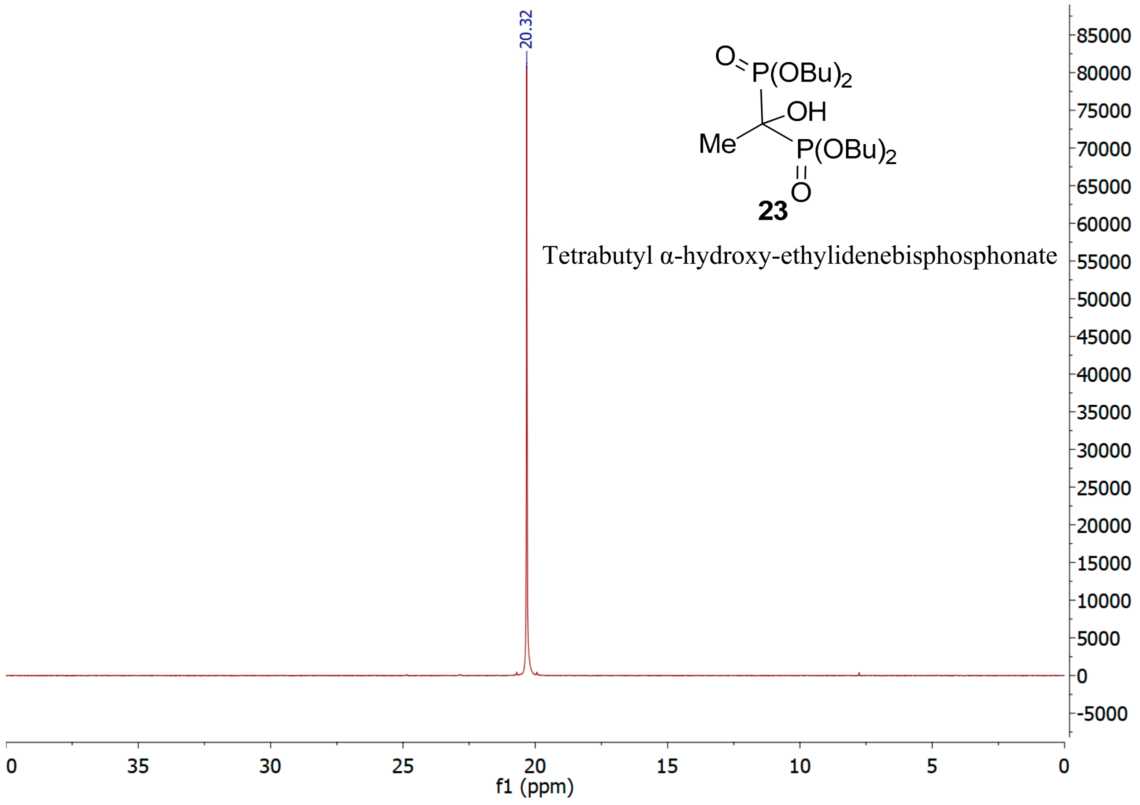

$^{13}C\ \{^{1}H\}$ NMR (126 MHz, $CDCl_3$) spectra for 23

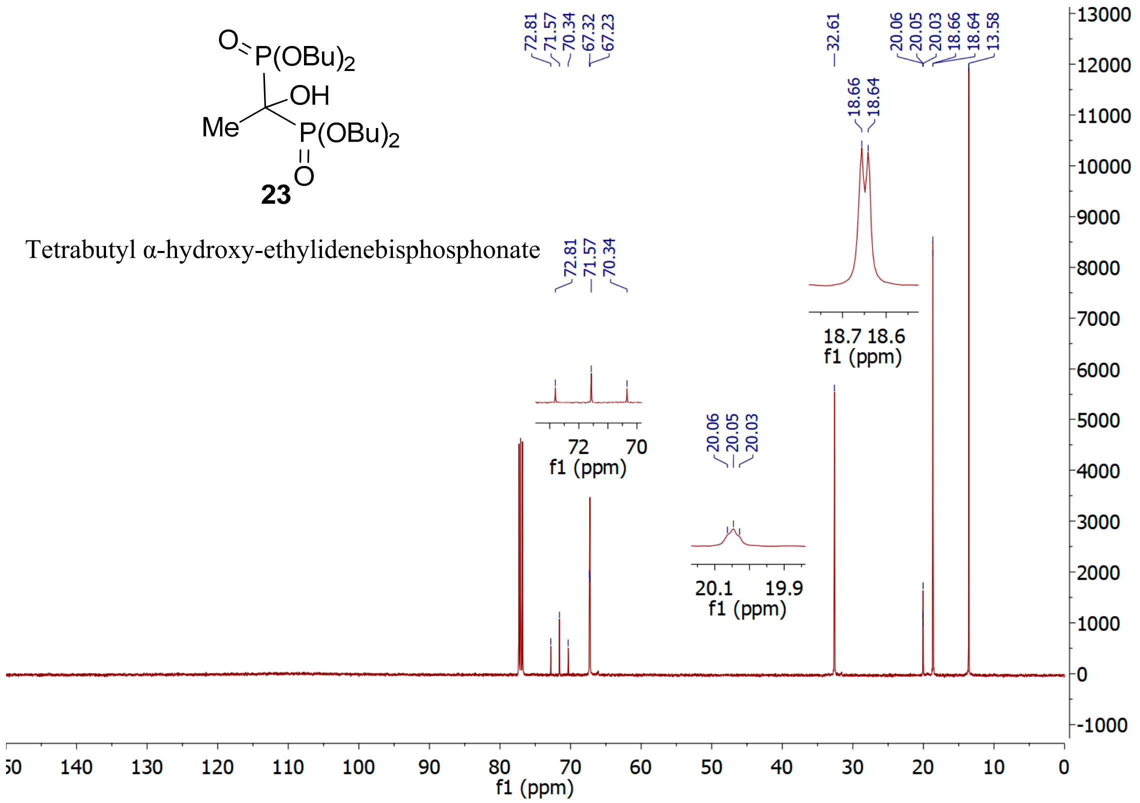


$^{1}H$ NMR (500 MHz, $CDCl_3$) spectra for 23

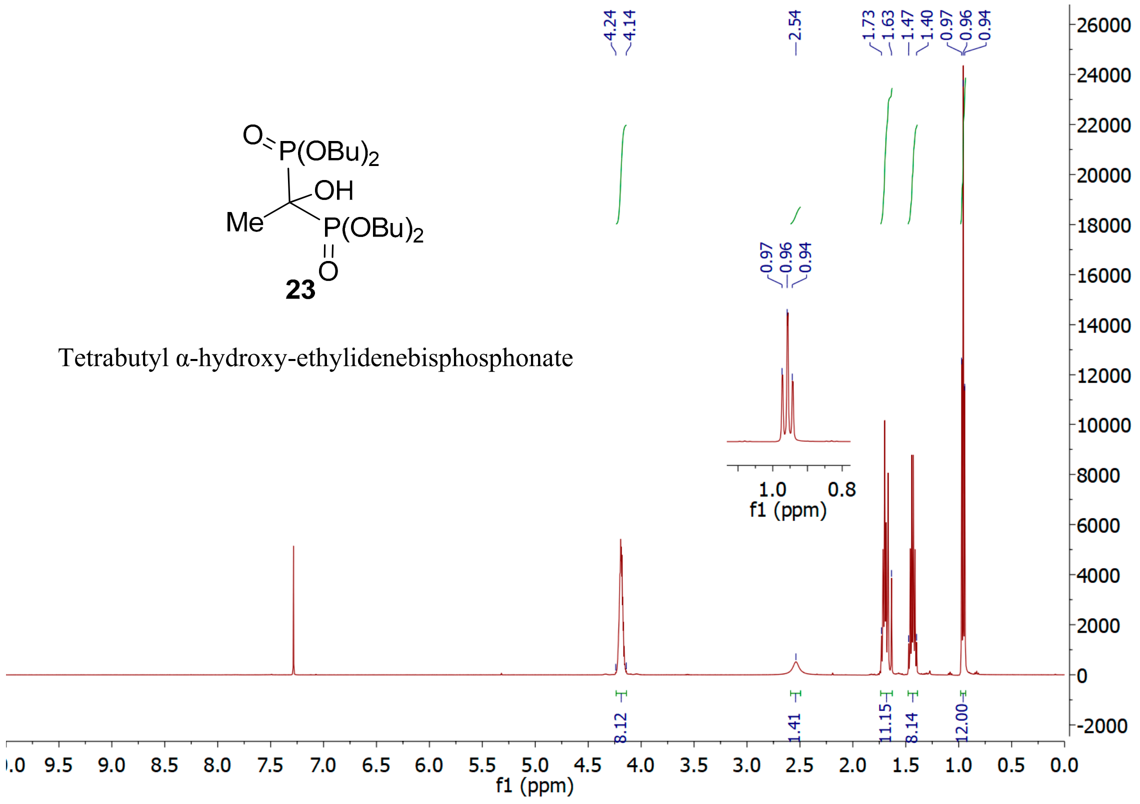

## $^{31}P$ {$^{1}H$} NMR (202 MHz, $CDCl_3$) spectra for 27

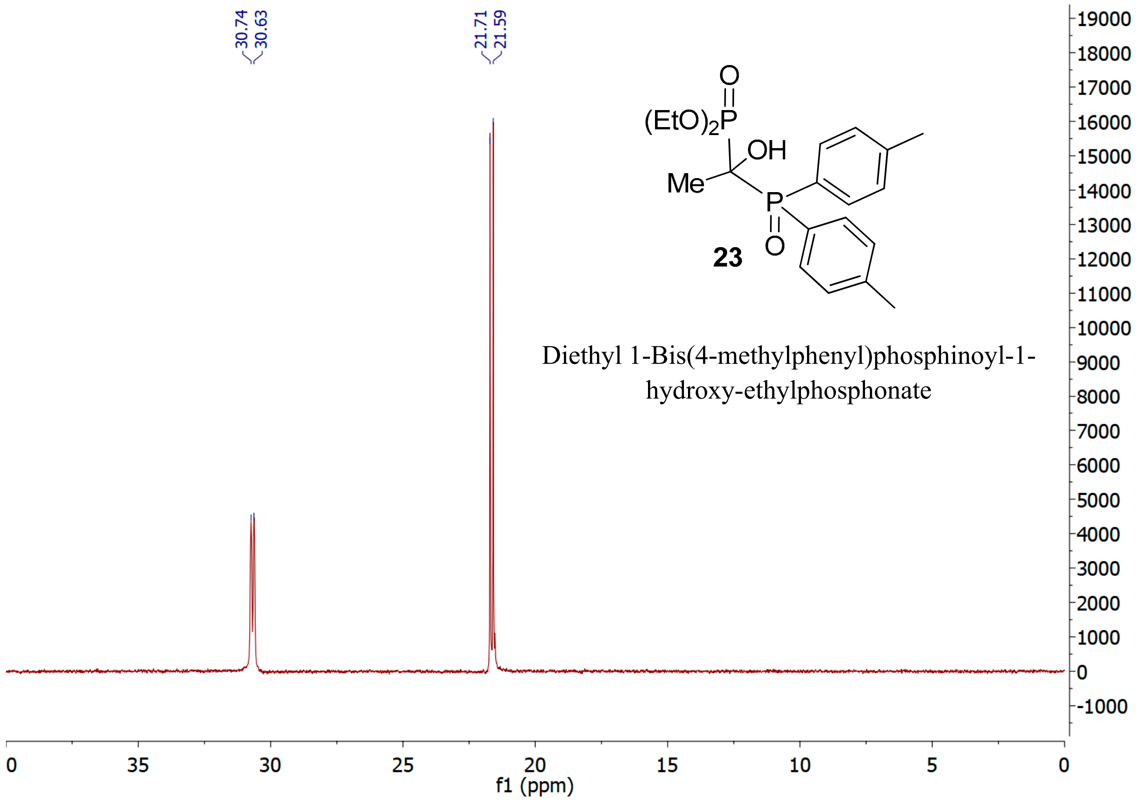


## $^{13}C$ {$^{1}H$} NMR (126 MHz, $CDCl_3$) spectra for 27

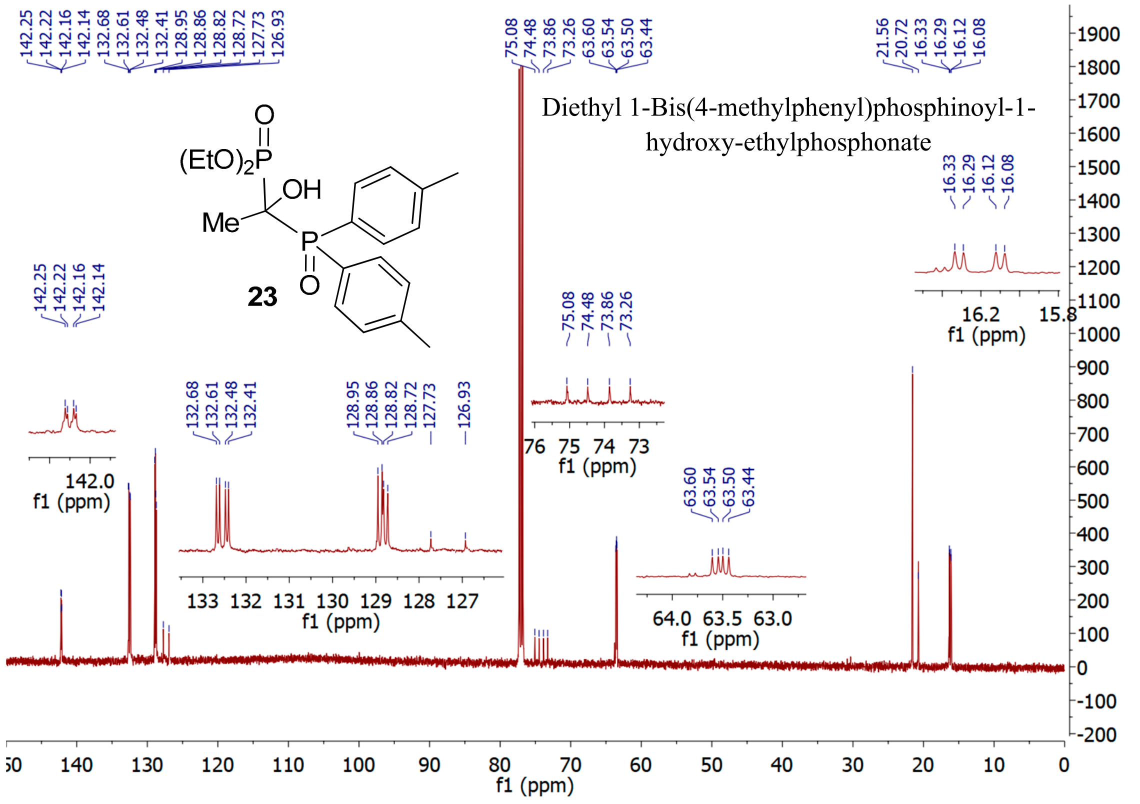

## $^{1}$H NMR (500 MHz, CDCl₃) spectra for 27

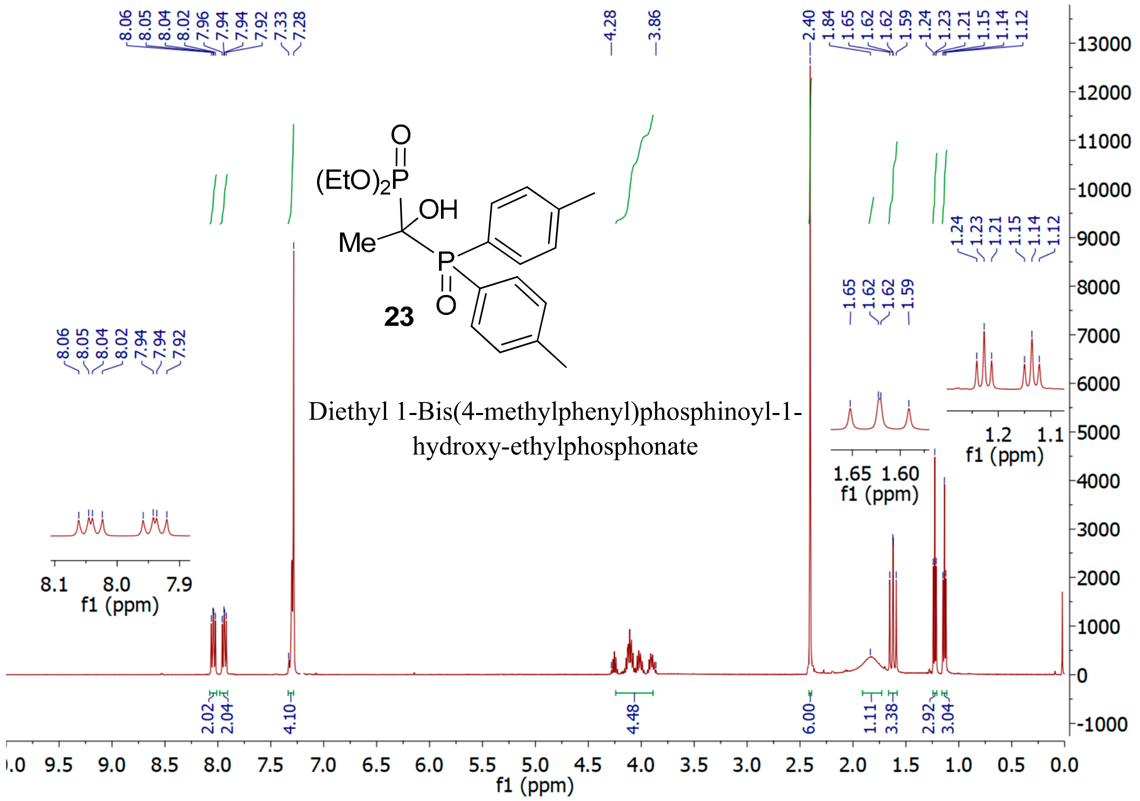

Supplement: Supplementary file 1 [file pharmaceuticals-18-00091-s001.zip › pharmaceuticals-3409248-supplementary.pdf]
